# Well-Defined and Robust Rhodium Catalysts for the Hydroacylation of Terminal and Internal Alkenes**

**DOI:** 10.1002/anie.201503208

**Published:** 2015-06-09

**Authors:** Amparo Prades, Maitane Fernández, Sebastian D Pike, Michael C Willis, Andrew S Weller

**Affiliations:** Department of Chemistry, University of OxfordMansfield Road, Oxford, OX1 3TA (UK)

**Keywords:** catalysis, hydroacylation, phosphine, rhodium

## Abstract

A Rh-catalyst system based on the asymmetric ligand ^*t*^Bu_2_PCH_2_P(*o*-C_6_H_4_OMe)_2_ is reported that allows for the hydroacylation of challenging internal alkenes with β-substituted aldehydes. Mechanistic studies point to the stabilizing role of both excess alkene and the OMe-group.

The catalytic hydroacylation reaction between an aldehyde and an olefin (or carbonyl group such as a ketone) is an attractive atom-efficient route to ketones (or esters).[1] Preeminent are transition metal catalysts based on cationic [Rh(bidentate phosphine)]^+^ although other catalyst systems are known.[2]

Irreversible reductive decarbonylation that arises from a coordinatively unsaturated acyl-hydride intermediate (Scheme 1),[3] removes catalyst from the system competitively with productive turnover, with the result that early studies used high catalyst loadings (5–10 mol %) and relatively activated substrates.[4] Control of decarbonylation can come from substrate chelation, such as β-substituted aldehydes or alkenes,[1a, 5] whereas the use of the hemilabile ligand bis-[2-(diphenylphosphino)phenyl]ether (DPEphos) that reversibly binds to the vacant site through a Rh⋅⋅⋅O linkage (Scheme 2), affords a long-lived catalyst, but activity is not significantly enhanced resulting in a rather limited scope of substrates.[6] An alternative approach is to increase the rate of reductive elimination of ketone product, a step that is often (although not exclusively[7]) the turnover-limiting process in hydroacylation.[3a, 8] Wide-bite-angle, or sterically bulky, ligands have this effect, but also change the ratio of alkene versus aldehyde hydroacylation[9] or the linear/branched selectivity.[10] Additionally, these systems in general still require high loadings, and activated alkenes or terminal alkynes as substrates. Small-bite-angle phosphine ligands, R_2_PCH_2_PR_2_ (e.g., R=^*t*^Bu, Cy), initially developed by Hofmann et al.,[11] have been shown to favor the products of reductive elimination[12] and we recently demonstrated that catalyst systems exemplified by [Rh(R_2_PCH_2_PR_2_)(η^6^-C_6_H_5_F)][BAr^F^_4_] [R=^*t*^Bu, **1**; Ar^F^=3,5-C_6_H_3_(CF_3_)_2_] can be used at low catalyst loadings (e.g., 0.1 mol %) to couple terminal and activated internal alkenes with β-substituted aldehydes.[13] However, challenging internal alkenes are still out of reach with this system, as decarbonylation now outruns productive turnover. Others have since used similar ligands for intermolecular hydroacylation.[5b, 7c]

**Scheme 1:**
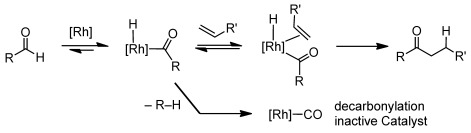
Alkene hydroacylation and decarbonylation.

**Scheme 2:**
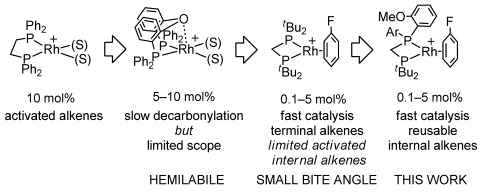
Development of chelating ligands in hydroacylation. (S)=solvent.

Small-bite-angle diphosphine ligands have also been used in alkene oligomerization reactions, in which reductive elimination is proposed to play a key role in determining selectivity.[14] Those with a “P(*o*-C_6_H_4_OMe)_2_” motif are particularly active, and solid-state structures indicate that the OMe group can interact with the metal center, although whether this leads to enhanced selectivity is yet to be delineated.[15] We thus speculated whether such a motif might offer improved catalyst turnover and/or stability for hydroacylation. We report here that systems based on asymmetric ligands such as ^*t*^Bu_2_PCH_2_P(*o*-C_6_H_4_OMe)_2_ (Scheme 2) are indeed particularly stable and active catalysts, allowing for the hydroacylation of a wide range of functionalized internal alkenes with β-substituted aldehydes.

The new asymmetric ligands **2 a**–**d**, R_2_PCH_2_PR'_2_, [R=^*t*^Bu, R'=*o*-C_6_H_4_OMe **2 a**, *o*-C_6_H_4_Et **2 b**, *p*-C_6_H_4_OMe **2 c**; R=^*i*^Pr, R'=*o*-C_6_H_4_OMe **2 d**] were synthesized[16] from the appropriate chlorophosphine ClPR'_2_ with Li[CH_2_PR_2_].[17] The corresponding precatalysts[13] were prepared from sequential addition of ligand and H_2_ to [Rh(cod)_2_][BAr^F^_4_] in C_6_H_5_F solution, to afford [Rh(**2**)(η^6^-C_6_H_5_F)][BAr^F^_4_], **3 a**–**d** (Scheme 3). The solid-state structures of **3 a** and **3 d**[16] (Figure 1 A shows **3 a**) show a Rh^I^ complex and no close Rh⋅⋅⋅⋅O contact [for example, Rh1–O2 3.381(3) Å]. The ^1^H NMR data (CD_2_Cl_2_) for **3 a** show the OMe groups at *δ* 3.54 (6 H), only slightly shifted upfield compared to free ligand (*δ* 3.67).

**Figure 1:**
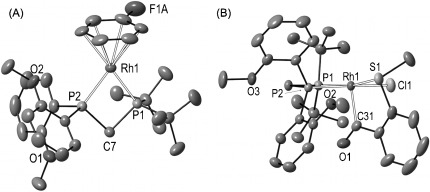
Solid-state structures of the cationic portion of complexes 3 a and 11. Thermal ellipsoids are shown at the 50 % probability level. Hydrogen atoms are not shown.[16]

**Scheme 3:**
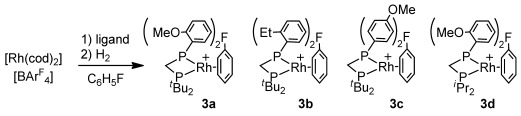
New catalysts used in this study. [BAr^F^_4_]^−^ anions omitted.

The new catalysts were screened in the hydroacylation reaction between 2-(methylthio)benzaldehyde (**4 a**) and 1-octene or cyclohexene to give ketones **5** and **6**, respectively, using conditions of high substrate concentration reported previously[13b] (Table 1), i.e., an aldehyde/alkene ratio of 1.5:4 (i.e., 1:2.7) and 1.3 mol % catalyst loading. The 1-octene catalyst **3 a** is particularly efficient, even at 0.13 mol % (entries 1 and 2). Replacement of the *o*-OMe substituent with isosteric ethyl (**3 b**, entry 3), electronically equivalent *p*-OMe (**3 c**, entry 4), ^*i*^Pr for ^*t*^Bu (entry 5), or use of the acetonitrile adduct [Rh{^*t*^Bu_2_PCH_2_P(*o*-C_6_H_4_OMe)_2_}(NCMe)_2_][BAr^F^_4_], **7** (entry 6), resulted in slower reactions. In all cases only linear product was observed. Although **1** operates with 1-octene comparably to **3 a** (entry 7) under these conditions, **3 a** is superior for a challenging internal alkene as shown when cyclohexene is used (compare entries 8 and 9).

**Table 1:** Benchmarking the catalysts^[a]^ 
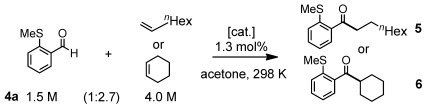

Entry	Catalyst	Olefin	*t*	Conversion [%]
1	**3 a**	1-octene	5 min	>95
2	**3 a^[c]^**	1-octene	15 h	>95
3	**3 b**	1-octene	1 h	>95
4	**3 c**	1-octene	6 h	>95
5	**3 d**	1-octene	3 h	>95
6	**7**	1-octene	1 h	>95
7	**1**	1-octene	5 min	>95
8	**3 a**	cyclohexene	3 h	61^[d]^
9	**1**	cyclohexene	3 h	30^[d]^

[a] Conditions: 1.5 m aldehyde, 4.0 m alkene (1:2.7), 0.02 m catalyst (1.3 mol %), acetone solvent, 298 K. [b] Conversion determined by HPLC. [c] 0.002 m catalyst. [d] After 5 h no significant increase in conversion was observed.

Comparing **3 a**–**c** with **1**, using 1-octene/**4 a**, all followed a first order profile for the production of **5** under these conditions; for example, **3 a:**­ *k*_obs_=(14.1±0.3)×10^−3^ s^−1^, **1:**­ *k*_obs_=(14.4±1.0)×10^−3^ s^−1^.[16] **3 a**, **3 b**, and **1** could be recharged with **4 a**/1-octene twice under these conditions, for example, **3 a**: *k*_obs_=(3.8±0.3)×10^−3^, (1.32±0.3)×10^−3^ s^−1^. **3 c** was inactive on recharging. The slower reaction on each recharge is accounted for by product inhibition, as shown by the reaction of a **5**/**4 a**/alkene mix of 1:1:2.7 which proceeds at a rate similar to that measured for the reuse of **3 a** [(2.45±0.05)×10^−3^ s^−1^]. Reducing the aldehyde/alkene ratio to a more equitable 1:1.5 ratio shows that catalyst **3 a** can still be reused to effect 100 % conversion, whereas **3 b** or **1** are essentially inactive. Below ratios of 1:1.3, catalysis did not reach completion suggesting catalyst decomposition. Variation of the loading of catalyst **3 a** between 0.01 m and 0.04 m,[16] showed a first order dependence, in contrast to reports in which bimetallic cooperativity is proposed.[18] Overall, these data demonstrate the positive effect that *o*-C_6_H_4_OMe has on the ability of the catalyst to operate, and be reused, at low relative alkene loadings or with an internal alkene, when compared to ^*t*^Bu (i.e., **1**).

In [D_6_]acetone the C_6_H_5_F ligand in **3 a** is replaced by acetone to form [Rh(**2 a**)(acetone)_2_][BAr^F^_4_].[13, 16] Subsequent addition of **4 a** afforded a complex mixture of products, in which the intermediate acyl hydride [Rh(**2 a**)(H)(κ,σ-1,2-SMe(CO)C_6_H_4_)(acetone)][BAr^F^_4_] (**8**) was identified [*δ*(H) −20.4, br].[10, 13, 16] Over 2.5 h this reaction proceeded to give the corresponding decarbonylation product [Rh(**2 a**)(SMePh)(CO)][BAr^F^_4_] (**9**). This timescale for decarbonylation is similar to that for **1**,[13b] suggesting that the OMe group does not offer strong stabilization to the acyl hydride in the absence of alkene. Use of the aldehyde 2-(diphenylphosphino)benzaldehyde (**4 b**) led to a more stable complex,[13b, 19] which did not decarbonylate, and formed as a single isomer [δ(H) −19.59, app. doublet of quartets]: [Rh(**2 a**)(H)(κ,σ-1,2-PPh_2_(CO)C_6_H_4_)(acetone)][BAr^F^_4_] (**10**, Scheme 4). The OMe groups are observed in the ^1^H NMR spectrum at *δ* 3.05 and *δ* 3.18 as sharp singlets, shifted upfield from the free ligand, suggesting that they do not interact strongly with the metal center, as OMe signals have been shown to shift downfield on coordination.[20] Addition of the corresponding acyl chloride **4 c** to **3 a** resulted in a structurally characterized complex [Rh(**2 a**)(Cl)(κ,σ-1,2-SMe(CO)C_6_H_4_)][BAr^F^_4_] (**11**; Figure 1 B). Although this shows a different relative orientation of ligands compared with that determined spectroscopically for **10** (and by inference **8**), in which, most likely due to the π-donor chloride, the acyl group is *trans* to the vacant site, it also shows that the OMe group does not approach the metal center [Rh1–O2 3.682 Å].

**Scheme 4:**
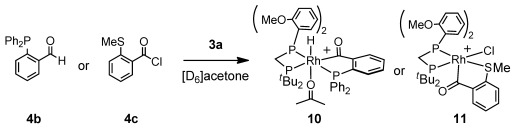
Complexes 10 and 11. [BAr^F^_4_]^−^ anions omitted.

Isotope labeling experiments during catalysis using **3 a**/[D]-**4 a**/1-octene showed a small, but significant, kinetic isotope effect (KIE) of 1.4±0.1. These experiments also reveal a relative 40:60 distribution of the [D]-label between α- and β-positions in the product **5**. Use of 2-[D]-1-octene (≈50 % D) gave the same relative isotopic distribution, Scheme 5. Hydride (deuteride) insertion into 1-octene is thus fast and reversible, giving both branched (**B**) and linear (**C**) intermediates. That no branched final product is observed suggests a higher barrier to reductive elimination from the corresponding intermediate, as noted previously.[8, 13b] H/D exchange was observed into free **4 a** during catalysis, suggesting reversible aldehyde and alkene binding. The measured KIE is similar to other systems in which reductive elimination is proposed to be turnover-limiting,[8b, 13] rather than irreversible C–H activation (KIE ≈2.5[7c, 18]), and is likely an equilibrium isotope effect.[21] Addition of just 1-octene to **3 a** results in isomerization to give a mixture of internal alkenes, that are not seen during catalysis when **4 a** is also present.

**Scheme 5:**
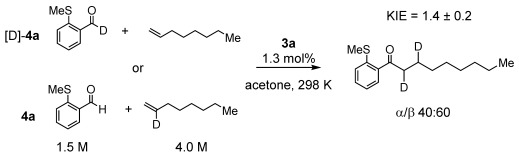
Labeling experiments.

A mechanism shown in Scheme 6 is suggested, in which the turnover-limiting step is the reductive C–C bond formation to generate the product complex **12** (rapid and reversible hydride insertion, KIE of 1.4), with alkene binding preceded by oxidative addition of aldehyde. At low relative [alkene] reductive decarbonylation and the formation of **9** becomes competitive. Based on the recharging experiments, binding of the product reversibly removes catalyst from the system. Consistent with this, complex **12** is observed as the resting state, and has been independently synthesized, by addition of **5** to **3 a**.[16] Addition of **4 a** to **12** in the absence of alkene results in the formation of **9**.

**Scheme 6:**
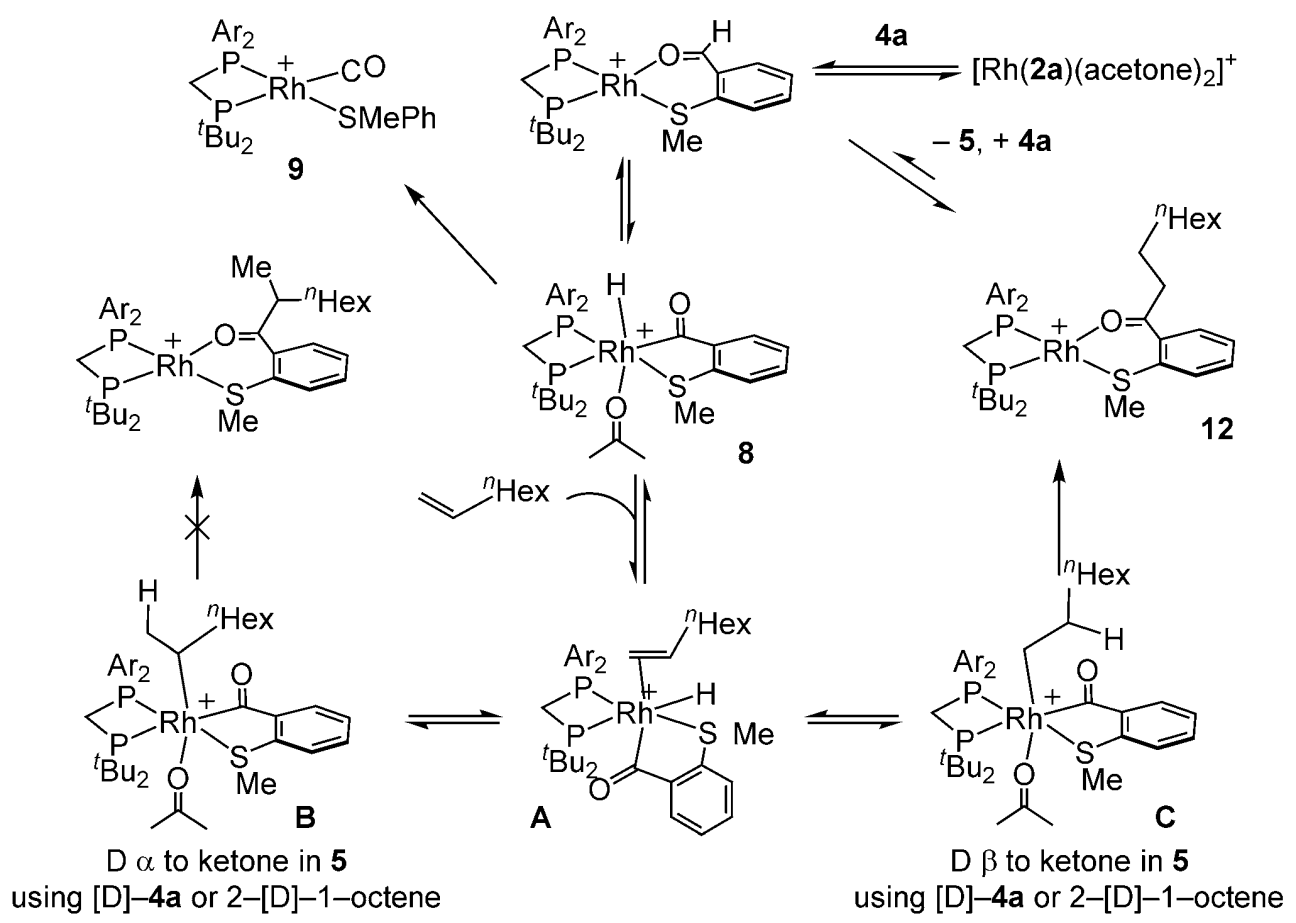
Proposed mechanism using 1-octene. [BAr^F^_4_]^−^ anions omitted.

With this stable catalyst in hand, we next explored the scope of its utility in intermolecular alkene hydroacylation reactions. In particular, we focused on the use of disubstituted alkenes; substrates that have previously proved difficult, or completely inactive in this reaction (Table 2). We first demonstrated that employing this new catalyst allowed reduced catalyst loadings for substrates previously reported.[13b] For example, using complex **1** a catalyst loading of 5 mol % and 80 °C were required for the hydroacylation of methyl methacrylate with a yield of 68 %; however, when catalyst **3 a** was employed, we were able to not only decrease the loading and the temperature but also significantly increase the yield (entry 1). A similar improvement was observed in the case of *N*-methyl maleimide (entry 2). More importantly, catalyst **3 a** allowed the use of internal alkenes that were previously only poorly reactive in this transformation. For example, using aldehyde **4 a**, cycloalkenes could be readily hydroacylated in 3 h (entries 3 and 4). Two examples of potentially isomerizable alkenes were then explored, with methylenecyclohexane and 3-methylcyclohexene both delivering the expected adducts in good yields (entries 5 and 6). The latter example, an unsymmetrical internal alkene, also gave high regiocontrol (>20:1). Cyclic enol ethers also performed with excellent efficiency and high regioselectivity when employing even lower catalyst loadings (entries 7 and 8). Remarkably, d-glucal, containing three unprotected hydroxy groups, could also be readily incorporated to deliver a *C*-gylcoside-type product (entry 9). Hydroacylation of a noncyclic enol ether was also efficient and displayed the same sense of regiocontrol as the cyclic counterparts (entry 10). Hydroacylation of α,β-unsaturated esters could be achieved in an efficient, regioselective fashion, although the cinnamate example was less reactive (entries 11–14). The regiocontrol observed for the ester series of substrates is in contrast (electronically) to that observed with the enol ether substrates, and is the subject of further investigation. Finally, we established that non-aryl aldehydes could also be employed, with a dihydropyran-derived aldehyde and two alkyl aldehydes proving to be competent substrates (entries 15–17). This scope study clearly demonstrates the exceptional reactivity and consequent utility of catalyst **3 a** for the hydroacylation of both 1,1- and 1,2-disubstituted alkenes.

**Table 2:** Scope of disubstituted alkene hydroacylation using catalyst 3 a^[a]^

Entry	Aldehyde	Alkene	Loading [mol %]	Product^[b]^	Yield [%]
1	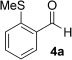	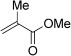	3	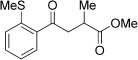	95
2	**4 a**	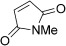	1	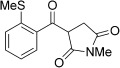	98^[c]^
3	**4 a**	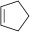	5	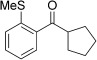	85
4	**4 a**	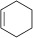	5	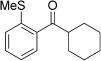	60
5	**4 a**	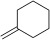	5	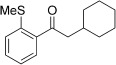	96
6	**4 a**	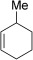	5	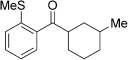	65^[d]^
7	**4 a**	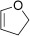	1	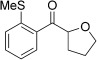	94
8	**4 a**	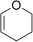	3	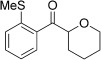	94
9	**4 a**	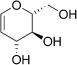	5	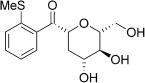	85^[e]^
10	**4 a**	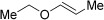	5	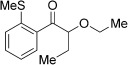	91^[f]^
11	**4 a**	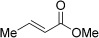	5	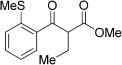	90^[c]^
12	**4 a**	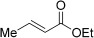	5	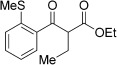	84^[c]^
13	**4 a**	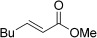	5	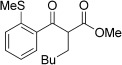	67^[c]^
14	**4 a**	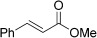	5	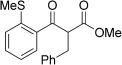	33^[c]^
15	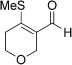	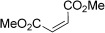	5	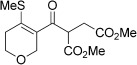	72^[g]^
16	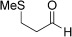	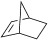	5	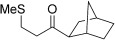	53
17	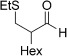	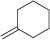	5	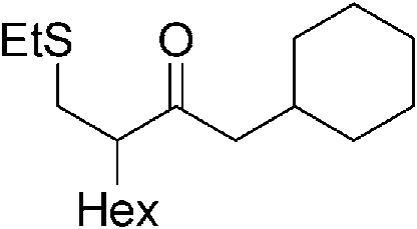	61

[a] Reaction conditions: Aldehyde (1.0 equiv, 2.0 m), alkene (1.5 equiv), catalyst **3 a**, acetone, 55 °C, 3 h. [b] Yield of isolated product. [c] Isolated as mixture of tautomers.[16] [d] Isolated as a 6:1 mixture of diastereoisomers, along with a minor product derived from methylenecyclohexane starting material contamination.[16] [e] Isolated as a 4:1 mixture of diastereoisomers. [f] Reaction performed using: Aldehyde (1.0 equiv, 8.0 m), alkene (2.7 equiv). [g] Reaction performed in dichloroethane at 80 °C.

Labeling experiments using [D]-**4 a** and methyl crotonate showed a 95:5 ratio of D-incorporation into the β/α positions, respectively, whereas for 2,3-dihydrofuran D-incorporation occurred exclusively at the 3-position; for both reactions significant [H]-**4 a** was observed after 60 % conversion (Scheme 7). This is consistent with a selective (in contrast to with 1-octene) but reversible insertion step. The dihydrofuran reaction represents a formal *trans* H-elimination from the insertion intermediate.[22] Interestingly, when [D]-**4 a** and cyclooctene were subjected to catalytic conditions, no turnover was detected, but selective H/D exchange at the alkene, alongside [H]-**4 a**, was observed. This unexpected selectivity hints at a richer mechanistic landscape with these substrates that warrants further detailed investigation.

**Scheme 7:**
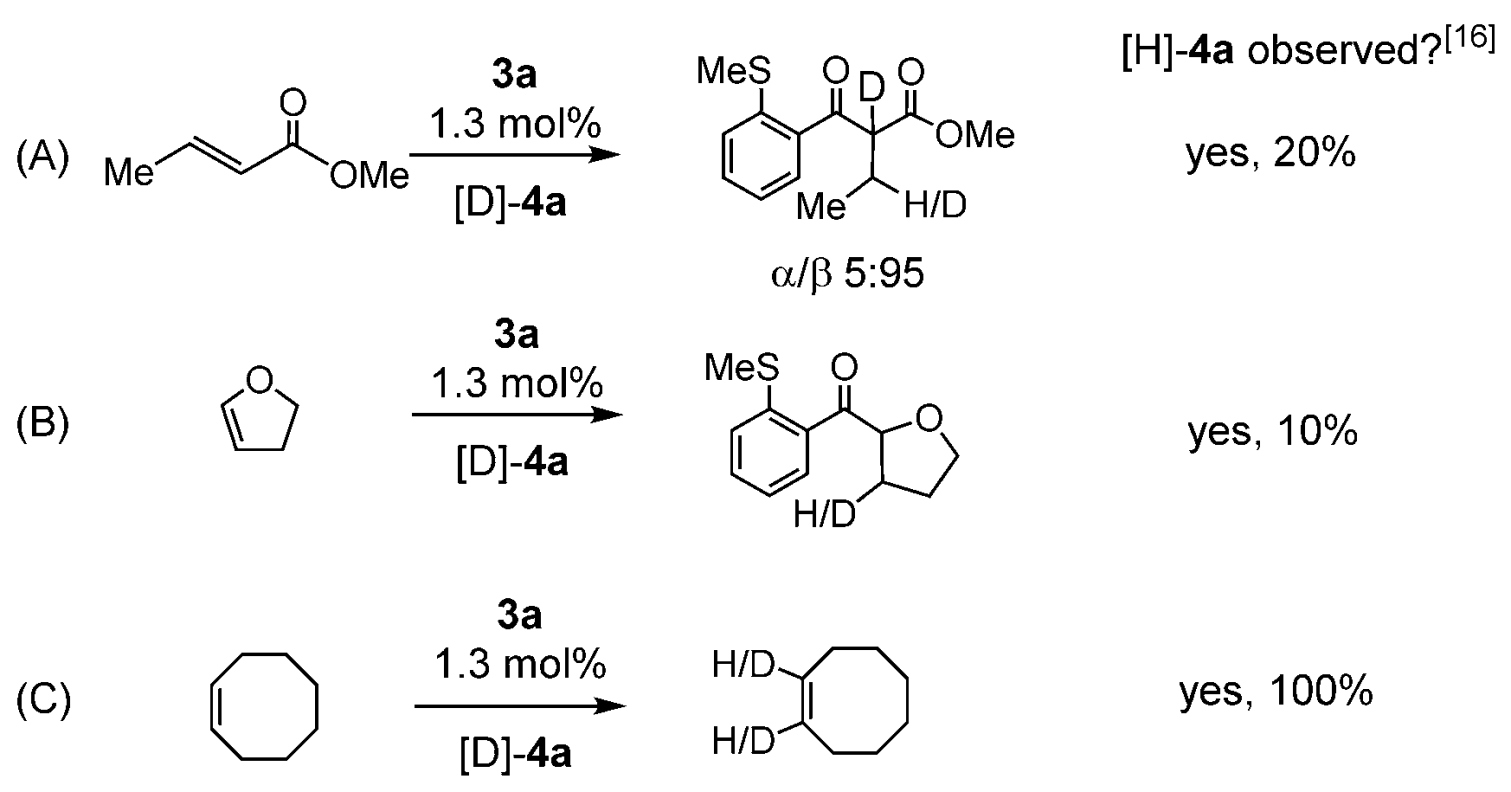
H/D exchange with internal alkenes. Reactions stopped after 60 % conversion.[16] Conditions [D_6_]acetone, 298 K, 1.5:4.0 m [D]-4 a/alkene.

Taken together, these data suggest that **3 a** works well at low relative alkene concentrations, or when the alkene binds less strongly, as the *o*-OMe groups in **3 a** are particularly good at promoting hydride insertion to form the branched or linear intermediates (**B** or **C** Scheme 6). The preceding acyl hydride (i.e., **8**) is thus disfavored, and decarbonylation is attenuated. We suggest that this is primarily a steric effect, as **3 c** (*p*-OMe) does not show the same activity. The promoting effect of *o*-Me groups in small-bite-angle ligands has previously been noted in CO/ethene copolymerization and ethene polymerization catalysis.[23]

In conclusion, we present a robust hydroacylation catalyst that allows for the coupling of challenging internal, previously unusable, functionalized alkenes, with β-substituted aldehydes. This also allows for a thorough mechanistic study and the realization of a broad alkene scope for this transformation.

## Supporting Information

As a service to our authors and readers, this journal provides supporting information supplied by the authors. Such materials are peer reviewed and may be re-organized for online delivery, but are not copy-edited or typeset. Technical support issues arising from supporting information (other than missing files) should be addressed to the authors.

miscellaneous_information
