# Supplementary material for: Well-Defined and Robust Rhodium Catalysts for the Hydroacylation of Terminal and Internal Alkenes
Source: Angew Chem Int Ed Engl. 2015 Jun 9;54(29):8520–4. doi: 10.1002/anie.201503208 (PMC4531818; doi:10.1002/anie.201503208)
Supplement: Supplementary file 1 — miscellaneous_information [file anie0054-8520-sd1.pdf]

Angewandte Chemie
Eine Zeitschrift der Gesellschaft Deutscher Chemiker

Supporting Information

Well-Defined and Robust Rhodium Catalysts for the Hydroacylation of Terminal and Internal Alkenes**

Amparo Prades, Maitane Fernández, Sebastian D. Pike, Michael C. Willis, and Andrew S. Weller**

anie_201503208_sm_miscellaneous_information.pdf

General experimental methods S-1
Synthesis of ligands S-2
Synthesis of Rhodium compounds S-8
Screening of catalysts S-12
Recycling S-12
Kinetics S-17
NMR scale reactions S-20
Labelling studies S-22
Crystallography S-29
Details of the synthesis of the new organic compounds S-32
References S-50

General experimental methods

All manipulations were performed under an atmosphere of argon, using Schlenk and glove box techniques. Glassware was oven dried at 130°C overnight and flamed under vacuum prior to use. CH_2Cl_2, MeCN, THF, Et_2O, hexane and pentane were dried using a Grubbs type solvent purification system (MBraun SPS-800) and degassed by successive freeze-pump-thaw cycles.[1] Acetone (<0.0075% H_2O) was purchased from VWR and degassed by successive freeze-pump-thaw cycles. CD_2Cl_2 and C_6H_5F were dried over CaH_2, vacuum distilled and stored over 3 Å molecular sieves. D_6–Acetone was dried over B_2O_6 and vacuum distilled, twice. 2-(methylthio)benzaldehyde (**4a**) was purchased from Sigma-Aldrich and purified by flash chromatography (5% Et_2O/petrol) and distilled (145°C, 13 mmHg) prior to use. $[Rh(COD)_2][BArF_4]$ (COD = 1,5-Cyclooctadiene),[2] bisethylphenylchlorophosphine,[3] bisisopropylchlorophosphine,[3] $[Rh(C_6H_5F)(t\mathrm{Bu}_2PCH_2Pt\mathrm{Bu}_2)][BAr^F_4]$ (**1**)[4] and acyl chloride (**4c**)[5] were prepared using literature methods.

2-(diphenylphosphino)-benzaldehyde (**4b**) and bismethoxyphenylchlorophospines are commercial products and were used without further purification. NMR spectra were recorded on Bruker DPX 400 MHz, Bruker DRX 500 MHz and Bruker AVC 500 MHz spectrometers. Chemical shifts are quoted in ppm and coupling constants in Hz. Microanalyses were performed at Elemental Microanalysis Ltd. ESI-MS were recorded on a Bruker MicroOTOF-Q instrument.

Synthesis of Ligands

The new diphosphinomethane ligands were prepared using the procedure reported in the literature by Hofmann and co-workers for similar diphosphines.[6]

($R_2CH_2R'_2$) (**2a**, R = ^{t}Bu, R' = *o*-C_6H_4OMe; **2b** R = ^{t}Bu, R' = *o*-C_6H_4Et; **2c** R = ^{t}Bu, R' = *o*-C_6H_4OMe and **2d** R = ^{i}Pr, R' = *p*-C_6H_4OMe.

To a Schlenk flask charged with lithium methanide (R_2PCH_2Li) in dry THF was added a solution of R'_2PCl in THF at -78C and the mixture was stirred allowed to warm gradually to room temperature overnight. The THF is removed on vacuum and the product was extracted into pentane. Hydrolysis with NH_4Cl (4% solution in argon-saturated water) caused the solution to warm. The organic phase was extracted with more pentane, cannulated over $MgSO_4$ and filtered. The pentane was distilled off at room pressure resulting in a white solid for **2a** (yield: 61%), **2c** (yield: 65%) and **2d** (yield: 45%) and colourless oil for **2b** (yield: 60%).

2a:

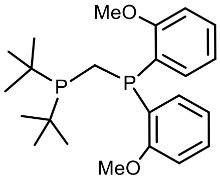

^{1}H NMR (CD_2Cl_2, 400MHz, 298K): δ 7.22-7.19 (m, 2H, CH_{PhOMe}), 7.19-7.15 (m, 2H, CH_{PhOMe}), 6.83-6.77 (m, 4H, CH_{PhOMe}), 3.67 (s, 6H, $CH_{3\ PhOMe}$), 2.20 (d, $^2J_{PH}$ = 3.2 Hz, 2H, PCH_2P) and 1.07 ppm (d, $^3J_{PH}$ = 10.8 Hz, 18H, $CH_{3\ tBu}$).

^{13}C NMR (CD_2Cl_2, 100.62 MHz, 298K): δ 161.6 (d, $^2J_{PC}$ = 12 Hz, Ph{C}OMe), 133.1 (dd, $^2J_{PC}$ = 12 Hz, $^4J_{PC}$ = 2 Hz, Ph{CH}OMe), 130.0 (s, Ph{CH}OMe), 126.6 (dd, $^1J_{PC}$ = 20 Hz, $^3J_{PC}$ = 6 Hz, OMePh{C}P), 120.6 (d, $^3J_{PC}$ = 3 Hz, Ph{CH}OMe), 110.5 (d, $^3J_{PC}$ = 1 Hz, Ph{CH}OMe), 55.6 (s, $CH_{3\ PhOMe}$), 31.8 (dd, $^1J_{PC}$ = 24 Hz, $^3J_{PC}$ = 6 Hz C_{tBu}), 29.7 (dd, $^2J_{PC}$ = 14 Hz, $^4J_{PC}$ = 2.5 Hz,$CH_{3\ tBu}$) and 19.2 ppm (dd, $^1J_{PC}$ = 21 Hz, $^1J_{PC}$ = 21 Hz, PCH_2P).

^{31}P NMR (CD_2Cl_2, 162MHz, 298K): δ 19.4 (d, $^2J_{PP}$ = 132 Hz, P(^{t}Bu)$_2$) and -28.4 ppm (d, $^2J_{PP}$ = 131 Hz, P(PhOMe)$_2$).

Anal. Calcd for $C_{23}H_{34}O_2P_2$ (404.27 gmol^{-1}): C, 68.30; H, 8.47; N, 0.00. Found: C, 68.14; H, 8.46; N, 0.00.

2b:

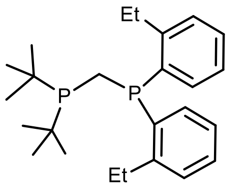

^{1}H NMR (CD_2Cl_2, 400MHz, 298K): δ 7.41-7.38 (m, 2H, CH_{PhEt}), 7.27-7.21 (m, 4H, CH_{PhEt}), 7.19-7.15 (m, 2H, CH_{PhEt}), 2.97 (q, $^3J_{HH}$ = 8 Hz, 4H, $CH_{2\ PhEt}$), 2.05 (d, $^2J_{PH}$ = 4 Hz, 2H, PCH_2P), 1.17 (t, $^2J_{HH}$ = 6 Hz, 6H, $CH_{3\ PhEt}$) and 1.11 ppm (d, $^3J_{PH}$ = 12 Hz, 18H, $CH_{3\ tBu}$).

^{13}C NMR (CD_2Cl_2, 100.62 MHz, 298K): δ 148.6 (d, $^2J_{PC}$ = 25 Hz, Ph{C}Et), 138.2 (dd, $^2J_{PC}$ = 17 Hz, $^4J_{PC}$ = 7 Hz, EtPh{C}P), 132.0 (s, Ph{CH}Et), 128.5 (s, Ph{CH}Et), 128.1 (d, $^3J_{PC}$ = 5 Hz, Ph{CH}Et), 125.7 (s, Ph{CH}Et), 31.7 (dd, $^1J_{PC}$ = 24 Hz, $^3J_{PC}$ = 6 Hz C_{tBu}), 29.5 (dd, $^2J_{PC}$ = 14 Hz, $^4J_{PC}$ = 2 Hz,CH_{3tBu}), 28.8 (d, $^3J_{PC}$ = 13 Hz, $CH_{2\ PhEt}$), 20.5 (dd, $^1J_{PC}$ = 22 Hz, $^1J_{PC}$ = 22 Hz, PCH_2P) and 15.0 ppm (s, $CH_{3\ PhEt}$).

^{31}P NMR (CD_2Cl_2, 162MHz, 298K): δ 14.2 (d, $^2J_{PP}$ = 130 Hz, $P(^tBu)_2$) and -42.5 (d, $^2J_{PP}$ = 131 Hz, $P(PhEt)_2$).

Microanalyses were not performed. The ligand was obtained with 90% purity (NMR characterization is showed below); even so the metal complex **3b** was obtained completely pure.

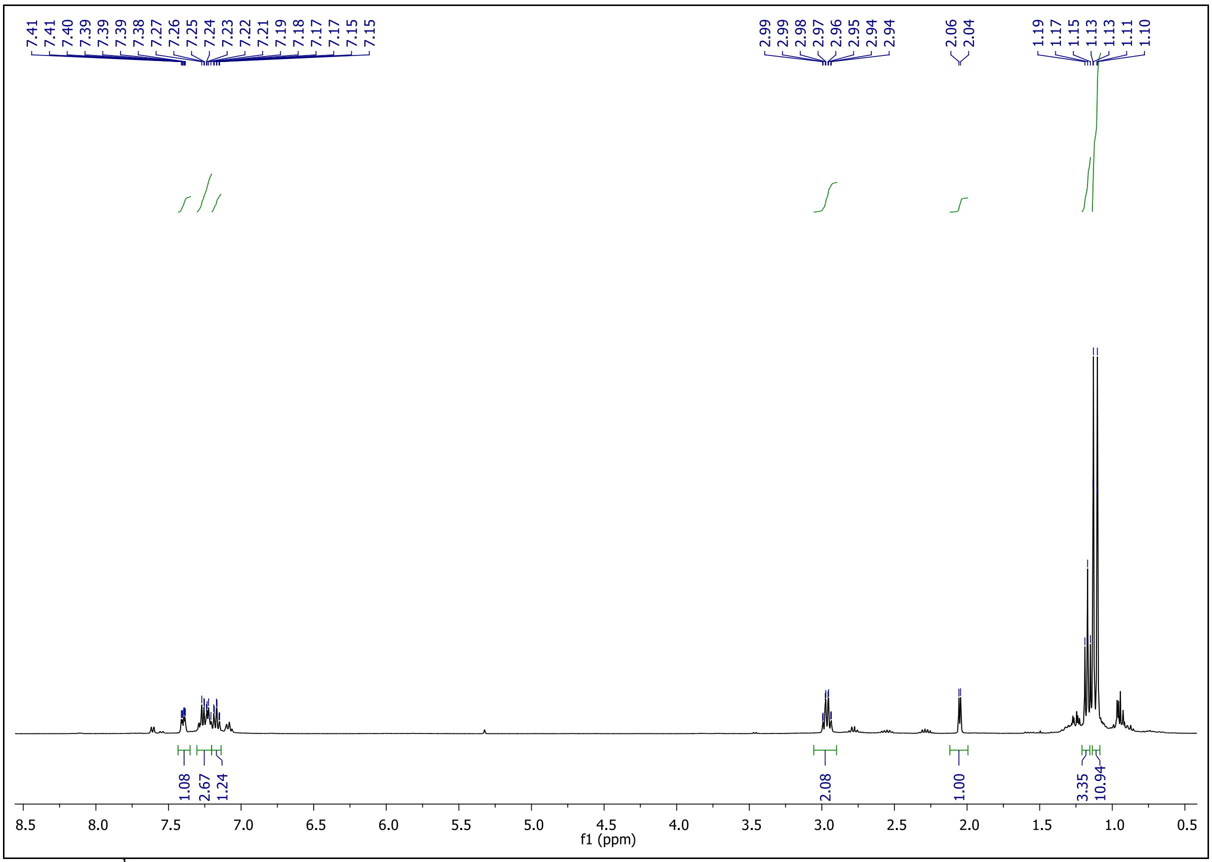

Figure S 1. ^{1}H NMR (400 MHz, CD_2Cl_2) of **2b.**

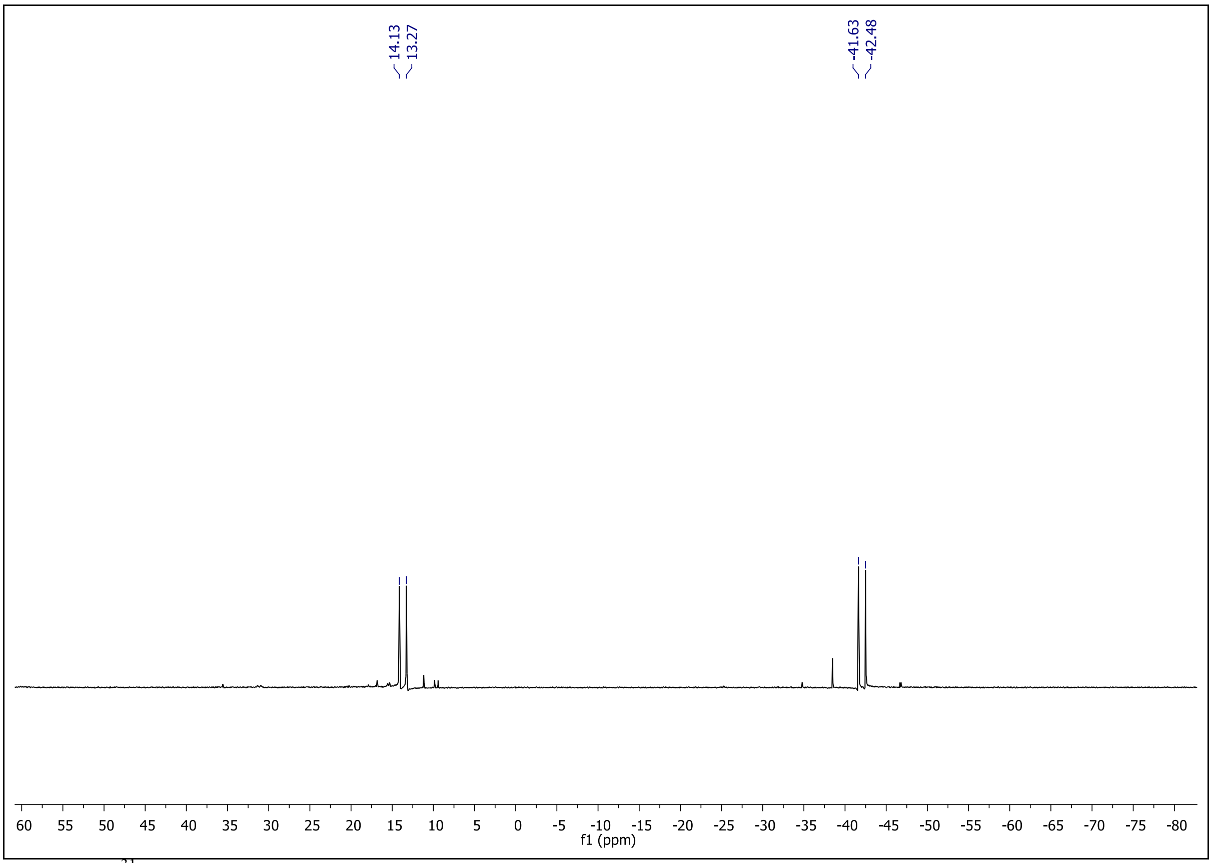

Figure S 2. ^{31}P NMR (162 MHz, CD_2Cl_2) of **2b.**

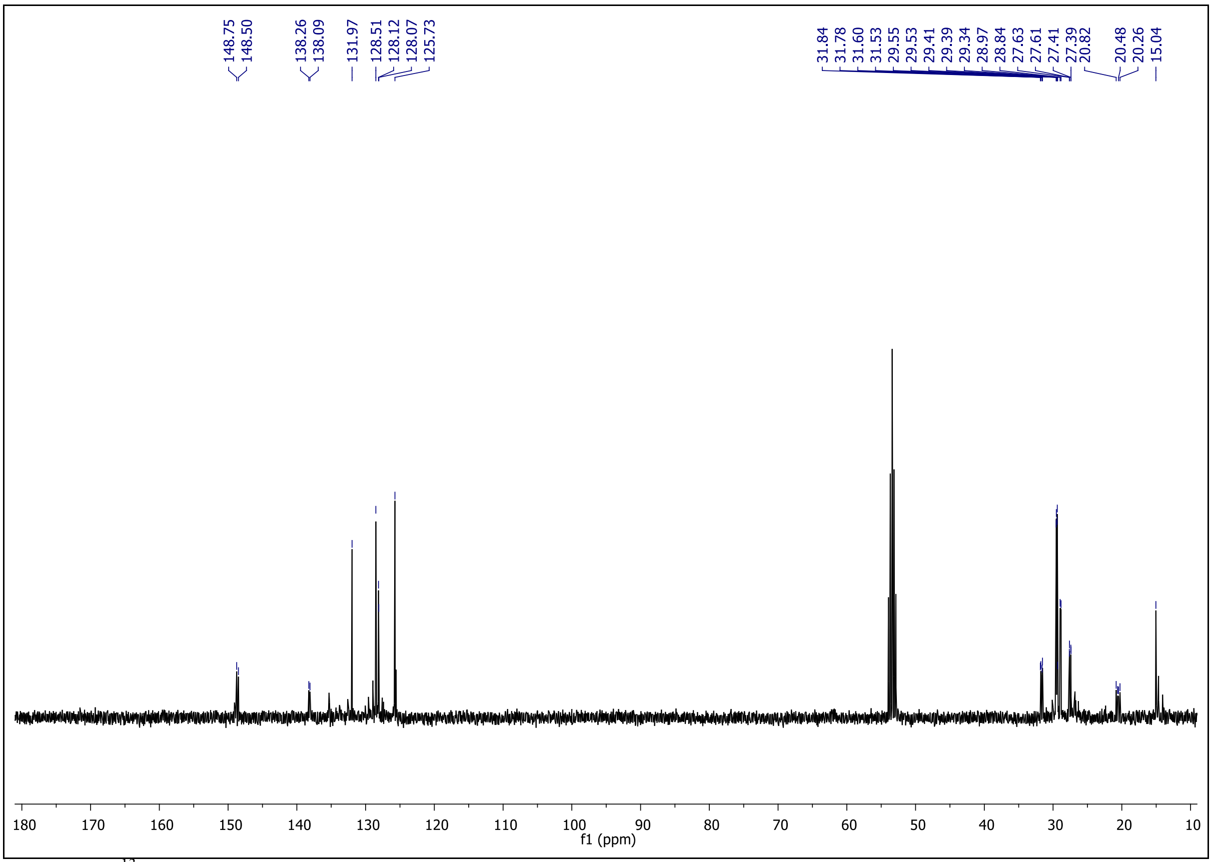

Figure S 3. ^{13}C NMR (100.62 MHz, CD_2Cl_2) of **2b.**

2c:

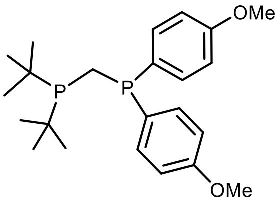

^{1}**H NMR** (CD_2Cl_2, 400MHz, 298K): δ 7.34-7.30 (m, 4H, CH_{PhOMe}), 6.78 (d, $^{2}J_{PH}$ = 8 Hz, 4H, 3.69 (s, 6H, $CH_{3\ PhOMe}$), 1.95 (d, $^{2}J_{PH}$ = 2.8 Hz, 2H, PCH_2P) and 1.01 ppm (d, $^{3}J_{PH}$ = 8 Hz, 18H, $CH_{3\ tBu}$).

^{13}**C NMR** (CD_2Cl_2, 100.62 MHz, 298K): δ 160.1 (s, Ph{C}OMe), 134.3 (d, $^{2}J_{PC}$ = 20 Hz, Ph{CH}OMe), 131.0 (dd, $^{1}J_{PC}$ = 14 Hz, $^{3}J_{PC}$ = 7 Hz, OMePh{C}P), 113.8 (d, $^{3}J_{PC}$ = 7 Hz, Ph{CH}OMe), 55.1 (s, $CH_{3\ PhOMe}$), 31.6 (dd, $^{1}J_{PC}$ = 24 Hz, $^{3}J_{PC}$ = 7 Hz C_{tBu}), 29.5 (dd, $^{2}J_{PC}$ = 14 Hz, $^{4}J_{PC}$ = 2.2 Hz,$CH_{3\ tBu}$) and 20.9 ppm (dd, $^{1}J_{PC}$ = 20 Hz, $^{1}J_{PC}$ = 20 Hz, PCH_2P).

^{31}**P NMR** (CD_2Cl_2, 162MHz, 298K): δ 16.4 (d, $^{2}J_{PP}$ = 136 Hz, $P(^{t}Bu)_2$) and -19.5 ppm (d, $^{2}J_{PP}$ = 136 Hz, $P(PhOMe)_2$).

Anal. Calcd for $C_{23}H_{34}O_2P_2$ (404.27 $gmol^{-1}$): C, 68.30; H, 8.47; N, 0.00. Found: C, 68.22; H, 8.52; N, 0.00.

2d:

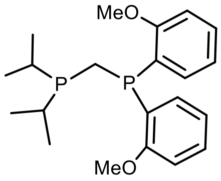

^{1}H NMR (CD_2Cl_2, 400MHz, 298K): δ 7.21-7.19 (m, 2H, CH_{PhOMe}), 7.10-7.07 (m, 2H, CH_{PhOMe}), 6.83-6.76 (m, 4H, CH_{PhOMe}), 3.67 (s, 6H, $CH_{3\ PhOMe}$), 2.06 (d, $^2J_{PH}$ = 3.2 Hz, 2H, PCH_2P), 1.72 (m, 2H, CH_{iPr}) 1.05 (dd, $^2J_{HH}$ = 11.5 Hz, $^3J_{PH}$ = 7.2 Hz, 6H, CH_{3iPr}) and 1.00 ppm (dd, $^2J_{HH}$ = 13.5 Hz, $^3J_{PH}$ = 7.2 Hz, 6H, $CH_{3\ iPr}$).

^{13}C NMR (CD_2Cl_2, 100.62 MHz, 298K): δ 161.4 (d, $^2J_{PC}$ = 12 Hz, Ph{C}OMe), 133.1 (dd, $^2J_{PC}$ = 8 Hz, $^4J_{PC}$ = 2 Hz, Ph{CH}OMe), 129.8 (s, Ph{CH}OMe), 126.6 (dd, $^1J_{PC}$ = 20 Hz, $^3J_{PC}$ = 5 Hz, OMePh{C}P), 120.6 (d, $^3J_{PC}$ = 3 Hz, Ph{CH}OMe), 110.3 (d, $^3J_{PC}$ = 3 Hz, Ph{CH}OMe), 55.3 (s, $CH_{3\ PhOMe}$), 24.3 (d, $^1J_{PC}$ = 15 Hz CH_{iPr}), 24.2 (d, $^1J_{PC}$ = 15 Hz CH_{iPr}), 19.6 (dd, $^2J_{PC}$ = 15 Hz, $^4J_{PC}$ = 3 Hz, $CH_{3\ iPr}$), 18.8 (dd, $^2J_{PC}$ = 15 Hz, $^4J_{PC}$ = 3 Hz, CH_{3iPr}) and 17.1 ppm (dd, $^1J_{PC}$ = 22 Hz, $^1J_{PC}$ = 22 Hz, PCH_2P).

^{31}P NMR (CD_2Cl_2, 162MHz, 298K): δ -2.7 (d, $^2J_{PP}$ = 120 Hz, $P(^iPr)_2$) and -36.6 ppm (d, $^2J_{PP}$ = 120 Hz, $P(PhOMe)_2$).

Microanalyses were not performed, NMR characterization is showed below.

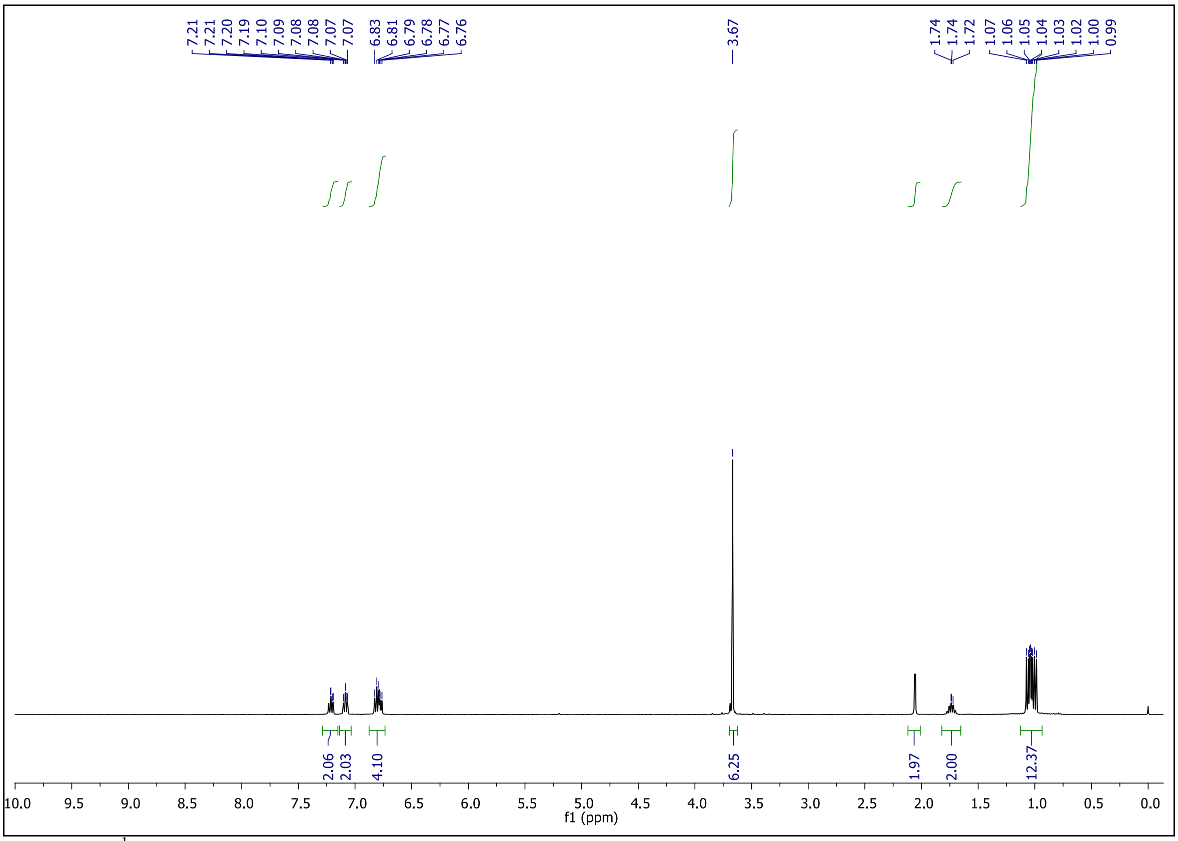

Figure S 4. ^{1}H NMR (400 MHz, CD_2Cl_2) of **2d.**

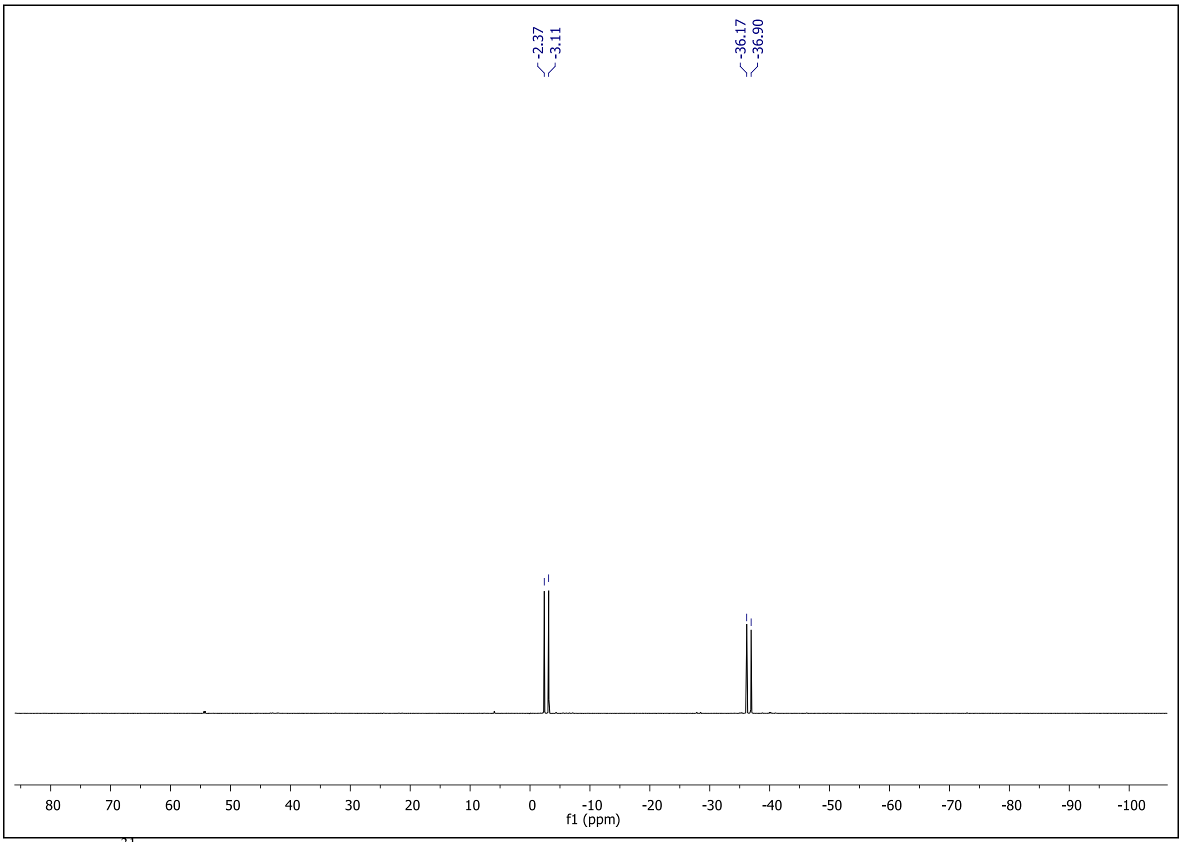

Figure S 5. ^{31}P NMR (162 MHz, CD_2Cl_2) of **2d.**

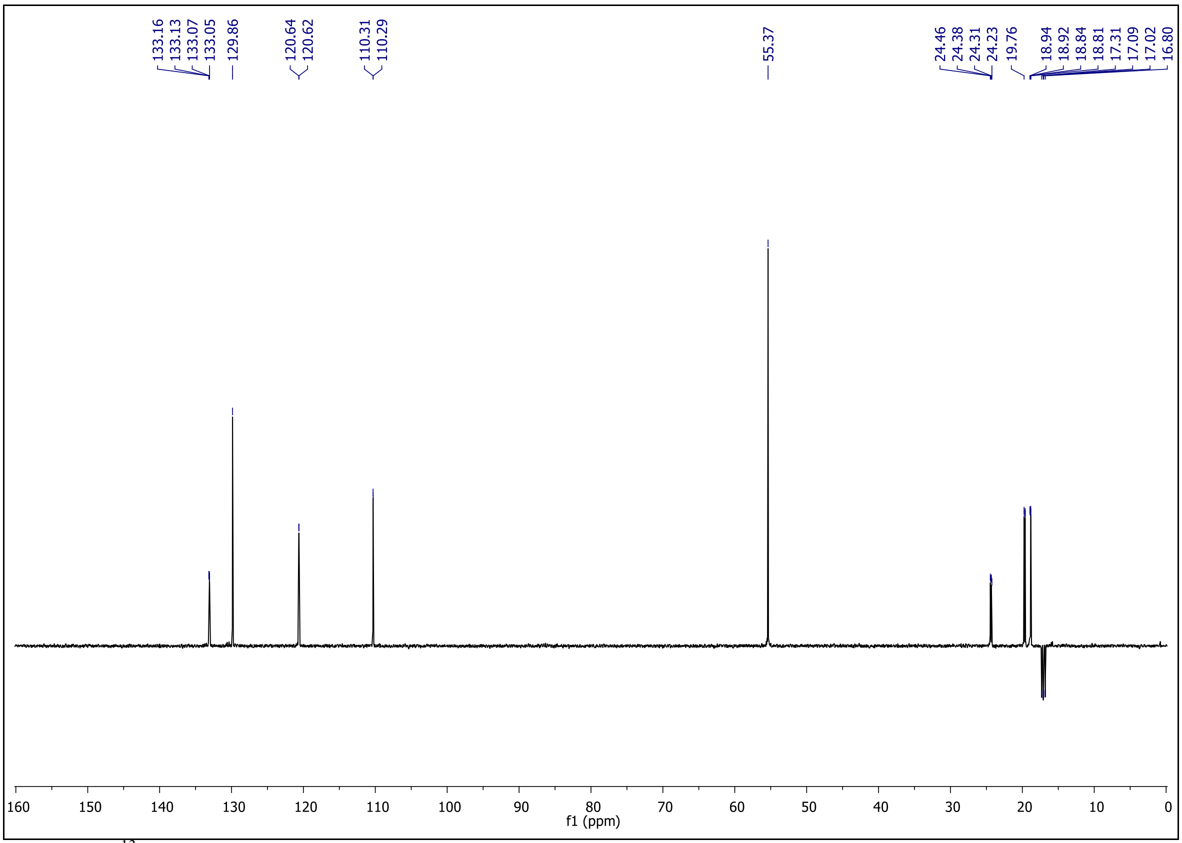

Figure S 6. ^{13}C NMR (100.62 MHz, CD_2Cl_2) of **2d**

Synthesis of Rhodium Complexes

Representative procedure for **3a**: To a Schlenk flask charged with $[Rh(COD)_2][BAr^F{}_4]$ (438 mg, 0.37 mmol) and $(^tBu)_2PCH_2P(PhOMe)_2$ (150 mg, 0.37 mmol) was added C_6H_5F (5mL) and the resulting solution placed under H_2 (4 atm) and stirred at room temperature overnight. The solvent was removed in vacuum and the product was precipitated by addition of pentane. The resulting yellow solid was filtered by cannula, washed with more pentane and dried in vacuum. Yield: 445 mg (82 %).

3a: Yield: 82 %.

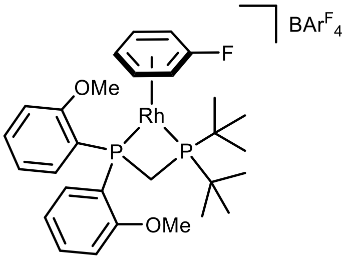

^{1}H NMR (CD_2Cl_2, 500MHz, 298K): δ 7.77 (br, 8H, CH_{ArF}), 7.70-7.65 (m, 2H, CH_{PhOMe}), 7.60 (br, 8H, CH_{ArF}), 7.60-7.48 (m, 2H, CH_{PhOMe}), 7.15-6.12 (m, 2H, CH_{PhOMe}), 6.68 (m, 2H, CH_{PhOMe}), 6.77 (m, 2H, $CH_{o\text{-}PhF}$), 6.67 (m, 2H, $CH_{m\text{-}PhF}$), 6.21 (m, 1H, $CH_{p\text{-}PhF}$), 3.54 (s, 6H, $CH_{3\ PhOMe}$), 3.54 (m, overlapped with $CH_{3\ PhOMe}$, 2H, PCH_2P) and 1.23 ppm (d, $^3J_{PH}$ = 15.5 Hz, 18H, $CH_{3\ tBu}$).

^{13}C NMR (CD_2Cl_2, 126 MHz, 298K): δ 161.7 (q, $^1J_{BC}$ = 50 Hz, CB_{BArF4}), 159.9 (s, Ph{C}OMe), 143.5 (d, $^1J_{FC}$ = 205 Hz, *i*-C_6H_5F), 134.8 (s, CH_{BArF4}), 133.2 (d, $^2J_{PC}$ = 14 Hz, Ph{CH}OMe), 132.9 (d, $^3J_{PC}$ = 3 Hz, Ph{CH}OMe), 128.8 (qq, $^2J_{FC}$ = 32 Hz, $^3J_{BC}$ = 4 Hz, ArF), 124.6 (q, $^1J_{FC}$ = 272 Hz, ArF), 120.7 (d, $^3J_{PC}$ = 14 Hz, Ph{CH}OMe), 120.4 (d, $^1J_{PC}$ = 40 Hz, PPh{C}OMe), 117.4 (sept, $^3J_{FC}$ = 4 Hz, ArF), 112.2 (d, $^3J_{PC}$ = 5 Hz, Ph{CH}OMe),

98.7 (d, $^3J_{FC}$ = 8 Hz, m-C_6H_5F), 94.5 (s, p-C_6H_5F), 90.8 (d, $^2J_{FC}$ = 20 Hz, o-C_6H_5F), 55.5 (s, $CH_{3\ PhOMe}$), 35.8 (d, $^1J_{PC}$ = 14 Hz, C_{tBu}), 29.9 (dd, $^1J_{PC}$ = 20 Hz, $^1J_{PC}$ = 20 Hz, PCH_2P) and 28.7 ppm (d, $^2J_{PC}$ = 5 Hz,$CH_{3\ tBu}$).

^{31}P NMR (CD_2Cl_2, 202MHz, 298K): δ 16.3 (dd, $^1J_{RhP}$ = 170 Hz, $^2J_{PP}$ = 99 Hz, $P(^tBu)_2$) and -41.7 ppm (dd, $^1J_{RhP}$ = 185 Hz, $^2J_{PP}$ = 99 Hz, $P(PhOMe)_2$).

^{19}F NMR (CD_2Cl_2, 282 MHz, 293 K): δ **-** 62.8 (s, 24F, ArF) and **-**123.0 ppm (s, 1F, C_6H_5F).

Anal. Calcd for $C_{61}H_{51}BO_2F_{25}P_2Rh$ (1466.21 $gmol^{-1}$): C, 49.95; H, 3.51; N, 0.00. Found: C, 49.96; H, 3.57; N, 0.00.

ESI-MS (Fluorobenzene, 60°C, 4.5 kV) positive ion: m/z, 603.147 $[M]^+$ (calc. 603.146).

3b: Yield: 75 %.

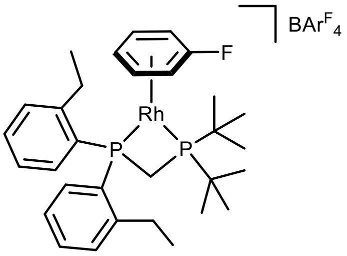

^{1}H NMR (CD_2Cl_2, 500MHz, 298K): δ 7.97-7.92 (m, 2H, CH_{PhEt}), 7.76 (br, 8H, CH_{ArF}), 7.60 (br, 8H, CH_{ArF}), 7.53-7.50 (m, 2H, CH_{PhEt}), 7.44-7.41 (m, 2H, CH_{PhEt}), 7.26 (m, 2H, CH_{PhOMe}), 6.80 (m, 2H, $CH_{o\text{-}PhF}$), 6.74 (m, 2H, $CH_{m\text{-}PhF}$), 6.22 (m, 1H, $CH_{p\text{-}PhF}$), 3.58 (m, 2H, PCH_2P), 2.38 (q, $^3J_{HH}$ = 5 Hz, 4H, $CH_{2\ PhEt}$), 1.26 (d, $^3J_{PH}$ = 20 Hz, 18H, $CH_{3\ tBu}$) and 0.48 ppm (t, $^2J_{HH}$ = 7.5 Hz, 6H, $CH_{3\ PhEt}$).

^{13}C NMR (CD_2Cl_2, 126 MHz, 298K): δ 161.7 (q, $^1J_{BC}$ = 50 Hz, CB_{BArF4}), 146.5 (d, , $^2J_{PC}$ = 8 Hz, Ph{C}Et), 134.8 (s, CH_{BArF4}), 132.3 (br, Ph{CH}Et), 131.9 (d, $^3J_{PC}$ = 3 Hz, Ph{CH}Et), 130.8 (d, $^2J_{PC}$ = 8 Hz, Ph{CH}Et), 130.6 (d, $^1J_{PC}$ = 44 Hz, EtPh{C}P), 128.8 (qq, $^2J_{FC}$ = 31.5 Hz, $^3J_{BC}$ = 2.5 Hz, ArF), 126.4 (d, $^3J_{PC}$ = 14 Hz, Ph{CH}Et), 124.6 (q, $^1J_{FC}$ = 273 Hz, ArF), 117.5 (sept, $^3J_{FC}$ = 3 Hz, ArF), 99.5 (d, $^3J_{FC}$ = 6 Hz, m-C_6H_5F), 94.7 (s, p-C_6H_5F), 91.2 (d, $^2J_{FC}$ = 20 Hz, o-C_6H_5F), 36.3 (d, $^1J_{PC}$ = 14 Hz, C_{tBu}), 32.0 (dd, $^1J_{PC}$ = 19 Hz, $^1J_{PC}$ = 19 Hz, PCH_2P), 28.8 (d, $^2J_{PC}$ = 5 Hz, CH_{3tBu}), 27.5 (d, $^3J_{PC}$ = 5 Hz, $CH_{2\ PhEt}$) and 13.8 ppm (s, $CH_{3\ PhEt}$).

^{31}P NMR (CD_2Cl_2, 202MHz, 298K): δ 15.7 (dd, $^1J_{RhP}$ = 168 Hz, $^2J_{PP}$ = 90 Hz, $P(^tBu)_2$) and -35.8 ppm (dd, $^1J_{RhP}$ = 184 Hz, $^2J_{PP}$ = 90 Hz, $P(PhEt)_2$).

^{19}F NMR (CD_2Cl_2, 282 MHz, 293 K): δ **-** 62.8 (s, 24F, ArF) and **-**122.0 ppm (s, 1F, C_6H_5F).

Anal. Calcd for $C_{63}H_{55}BF_{25}P_2Rh$ (1462.25 gmol^{-1}): C, 51.73; H, 3.79; N, 0.00. Found: C, 51.82; H, 3.71; N, 0.00.

ESI-MS (CH_2Cl_2, 60°C, 4.5 kV) positive ion: m/z, 599.2 $[M]^+$ (calc. 599.19).

3c: Yield: 92 %.

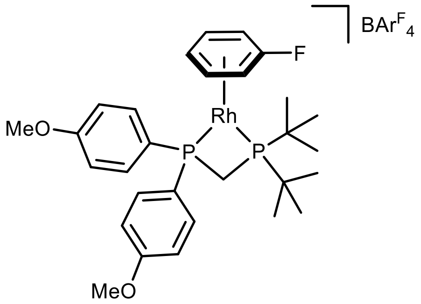

^{1}H NMR (CD_2Cl_2, 500MHz, 298K): δ 7.71 (br, 8H, CH_{ArF}), 7.55 (br, 8H, CH_{ArF}), 7.48-7.42 (m, 4H, CH_{PhOMe}), 7.00-6.95 (m, 4H, CH_{PhOMe}), 6.78 (m, 2H, $CH_{o\text{-}PhF}$), 6.70 (m, 2H, $CH_{m\text{-}PhF}$), 6.14 (m, 1H, $CH_{p\text{-}PhF}$), 3.81 (s, 6H, $CH_{3\ PhOMe}$), 3.27 (m, 2H, PCH_2P) and 1.18 ppm (d, $^3J_{PH}$ = 15.5 Hz, 18H, $CH_{3\ tBu}$).

^{13}C NMR (CD_2Cl_2, 126 MHz, 298K): δ 159.9 (d, $^4J_{PC}$ = 2.5 Hz, Ph{C}OMe), 161.9 (q, $^1J_{BC}$ = 65 Hz, CB_{BArF4}), 143.0 (d, $^1J_{FC}$ = 270 Hz, *i*-C_6H_5F), 134.9 (s, CH_{BArF4}), 133.6 (d, $^2J_{PC}$ = 18 Hz, Ph{CH}OMe), 128.9 (qq, $^2J_{FC}$ = 34 Hz, $^3J_{BC}$ = 4 Hz, ArF), 124.7 (q, $^1J_{FC}$ = 273 Hz, ArF), 123.6 (d, $^1J_{PC}$ = 53 Hz, PPh{C}OMe), 117.6 (sept, $^3J_{FC}$ = 4 Hz, ArF), 114.8 (d, $^3J_{PC}$ = 12.6 Hz, Ph{CH}OMe), 99.4 (d, $^3J_{FC}$ = 8 Hz, m-C_6H_5F), 93.4 (s, p-C_6H_5F), 91.3 (d, $^2J_{FC}$ = 20 Hz, o-C_6H_5F), 55.6 (s, $CH_{3\ PhOMe}$), 36.1 (d, $^1J_{PC}$ = 14 Hz, C_{tBu}), 33.6 (dd, $^1J_{PC}$ = 19 Hz, $^1J_{PC}$ = 19 Hz, PCH_2P) and 28.8 ppm (d, $^2J_{PC}$ = 5 Hz, $CH_{3\ tBu}$).

^{31}P NMR (CD_2Cl_2, 202MHz, 298K): δ 15.7 (dd, $^1J_{RhP}$ = 170 Hz, $^2J_{PP}$ = 101 Hz, $P(^tBu)_2$) and -30.5 ppm (dd, $^1J_{RhP}$ = 178 Hz, $^2J_{PP}$ = 101 Hz, $P(PhOMe)_2$).

^{19}F NMR (CD_2Cl_2, 282 MHz, 293 K): δ - 62.8 (s, 24F, ArF) and -123.0 ppm (s, 1F, C_6H_5F).

Anal. Calcd for $C_{61}H_{51}BO_2F_{25}P_2Rh$ (1466.21 gmol^{-1}): C, 49.95; H, 3.51; N, 0.00. Found: C, 49.89; H, 3.58; N, 0.00.

ESI-MS (Fluorobenzene, 60°C, 4.5 kV) positive ion: m/z, 603.147 $[M]^+$ (calc. 603.146).

3d: Yield: 79 %.

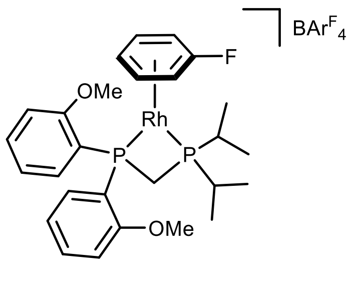

^{1}H NMR (CD_2Cl_2, 500MHz, 298K): δ 7.77 (br, 8H, CH_{ArF}), 7.63-7.59 (m, 2H, CH_{PhOMe}), 7.60 (br, 8H, CH_{ArF}), 7.51-7.48 (m, 2H, CH_{PhOMe}), 7.14-6.11 (m, 2H, CH_{PhOMe}), 6.87 (m, 2H, CH_{PhOMe}), 6.76 (m, 2H, $CH_{o\text{-}PhF}$), 6.66 (m, 1H, $CH_{m\text{-}PhF}$), 6.12 (m, 2H, $CH_{p\text{-}PhF}$), 3.59 (s, 6H, $CH_{3\ PhOMe}$), 3.47 (m, 2H, PCH_2P) 2.00 (m, 2H, CH_{iPr}) 1.06 (dd, $^2J_{HH}$ = 18.7 Hz, $^3J_{PH}$ = 7.0 Hz, 6H, $CH_{3\ iPr}$) and 1.00 ppm (dd, $^2J_{HH}$ = 17.0 Hz, $^3J_{PH}$ = 7.0 Hz, 6H, $CH_{3\ iPr}$).

^{13}C NMR (CD_2Cl_2, 100.62 MHz, 298K): δ 161.7 (q, $^1J_{BC}$ = 40 Hz, CB_{BArF4}), 159.9 (s, Ph{C}OMe), 143.4 (d, $^1J_{FC}$ = 205 Hz, *i*-C_6H_5F), 134.8 (s, CH_{BArF4}), 133.6 (d, $^2J_{PC}$ = 11 Hz, Ph{CH}OMe), 133.6 (d, $^3J_{PC}$ = 1 Hz, Ph{CH}OMe), 128.8 (qq, $^2J_{FC}$ = 26 Hz, $^3J_{BC}$ = 3 Hz, ArF), 124.8 (q, $^1J_{FC}$ = 218 Hz, ArF), 120.8 (d, $^3J_{PC}$ = 11 Hz, Ph{CH}OMe), 120.3 (d, $^1J_{PC}$ = 40 Hz, OMePh{C}P), 117.4 (sept, $^3J_{FC}$ = 3 Hz, ArF), 111.9 (d, $^3J_{PC}$ = 3 Hz, Ph{CH}OMe),
98.4 (d, $^3J_{FC}$ = 6 Hz, m-C_6H_5F), 93.7 (s, p-C_6H_5F), 90.2 (d, $^2J_{FC}$ = 17 Hz, o-C_6H_5F), 55.5 (s, $CH_{3\ PhOMe}$), 30.3 (dd, $^1J_{PC}$ = 18 Hz, $^1J_{PC}$ = 18 Hz, PCH_2P), 25.8 (d, $^1J_{PC}$ = 19 Hz, CH_{iPr}), 18.3 (s, CH_{3iPr}) and 17.6 ppm (s, CH_{3iPr}).

^{31}P NMR (CD_2Cl_2, 202MHz, 298K): δ 4.6 (dd, $^1J_{RhP}$ = 167 Hz, $^2J_{PP}$ = 111 Hz, $P(^iPr)_2$) and -40.3 ppm (dd, $^1J_{RhP}$ = 183 Hz, $^2J_{PP}$ = 111 Hz, $P(PhOMe)_2$).

^{19}F NMR (CD^2Cl^2, 282 MHz, 293 K): δ - 62.8 (s, 24F, ArF) and -124.0 ppm (s, 1F, C_6H_5F).

Anal. Calcd for $C_{59}H_{47}BO_2F_{25}P_2Rh$ (1438.18 $gmol^{-1}$): C, 49.26; H, 3.29; N, 0.00. Found: C, 49.38; H, 3.27; N, 0.00.

ESI-MS (CH_2Cl_2, 60°C, 4.5 kV) positive ion: m/z, 575.12 $[M]^+$ (calc. 575.12)

6:

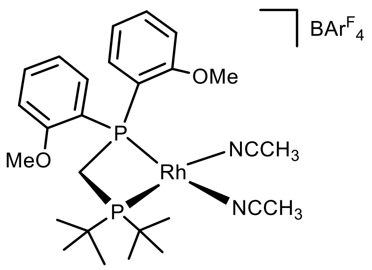

To a Schlenk flask charged with **3a** (100mg, 0.07mmol) was added CH_3CN (5mL) and the resulting solution was stirred during 30 minutes at room temperature. After that the solvent was removed and the yellow oil was precipitate with a mixture of Et_2O-Pentane. Yield 91% (90mg).

^{1}H NMR (CD_2Cl_2, 400MHz, 298K): δ 7.83-7.81 (m, 2H, CH_{PhOMe}), 7.64 (br, 8H, CH_{ArF}), 7.47 (br, 8H, CH_{ArF}), 7.34-7.30 (m, 2H, CH_{PhOMe}), 6.98-6.94 (m, 2H, CH_{PhOMe}), 6.73-6.70 (m, 2H, CH_{PhOMe}), 3.43 (s, 6H, $CH_{3\ PhOMe}$), 3.43 (m, overlapped with $CH_{3\ PhOMe}$, 2H, PCH_2P), 2.15 (br, 6H, $NCCH_3$) and 1.19 ppm (d, $^3J_{PH}$ = 14.4 Hz, 18H, $CH_{3\ tBu}$).

^{13}C NMR (CD_2Cl_2, 126 MHz, 298K): δ 159.6 (q, $^1J_{BC}$ = 50 Hz, CB_{BArF4}), 157.7 (s, Ph{C}OMe), 132.7 (d, $^2J_{PC}$ = 15 Hz, Ph{CH}OMe), 132.3 (s, CH_{BArF4}) 129.9 (d, $^3J_{PC}$ = 3 Hz, Ph{CH}OMe), 126.6

(qq, $^{2}J_{FC}$ = 32 Hz, $^{3}J_{BC}$ = 3 Hz, ArF), 122.4 (q, $^{1}J_{FC}$ = 272 Hz, ArF), 120.1 (br, N*C*CH_3), 118.5 (d, $^{1}J_{PC}$ = 43 Hz, OMePh{C}P), 118.2 (d, $^{3}J_{PC}$ = 13 Hz, Ph{CH}OMe), 115.2 (sept, $^{3}J_{FC}$ = 4 Hz, ArF), 109.6 (d, $^{3}J_{PC}$ = 4 Hz, Ph{CH}OMe), 53.1 (s, PhOMe), 32.6 (d, $^{1}J_{PC}$ = 14 Hz, C_{tBu}), 30.7 (dd, $^{1}J_{PC}$ = 18 Hz, $^{1}J_{PC}$ = 18 Hz, PCH_2P), 26.8 (d, $^{2}J_{PC}$ = 5 Hz,CH_{3tBu}) and 0.9 ppm (s, NC*CH_3*).

^{31}P NMR (CD_2Cl_2, 202MHz, 298K): δ 15.1 (dd, $^{1}J_{RhP}$ = 91 Hz, $^{2}J_{PP}$ = 64 Hz, P(^{t}Bu)$_2$) and -37.8 ppm (dd, $^{1}J_{RhP}$ = 97 Hz, $^{2}J_{PP}$ = 64 Hz, P(PhOMe)$_2$).

^{19}F NMR (CD_2Cl_2, 282 MHz, 293 K): δ **-** 62.8 ppm (s, 24F, ArF).

Anal. Calcd for $C_{59}H_{52}BN_2F_{24}P_2O_2Rh$ (1452.23 gmol^{-1}): C, 48.78; H, 3.61; N, 1.93. Found: C, 48.69; H, 3.56 ; N, 1.91.

ESI-MS (CH_2Cl_2, 60°C, 4.5 kV) positive ion: m/z, 589.17 $[M]^+$ (calc. 589.16).

Screening of catalysts

The catalytic runs were carried out under an Ar atmosphere. The mixture of the reagents (1.5 M of aldehyde and 4M of alkene, 1:2.7) in acetone was transferred to the metal catalyst (0.02M, 1.3 mol%) to give a homogeneous solution. Catalysis was followed by HPLC, the samples of the reaction mixture were quenched by dilution with 1.5 mL of acetonitrile. Reverse phase HPLC analysis was performed on Agligent Zorbax SB-C18 5µm column (4.6 x 150mm), eluted in 85% MeCN/H2O, 1 mL/min. Alkyne hydroacylation: t_1 (2-(metylthio)acetophenone) = 2.0 min, t_2 (linear 1-octyne product) = 5.0 min, t_3 (branched 1-octyne product) = 5.5 min. Alkene hydroacylation: t_1 (2-(metylthio)acetophenone) = 2.0 min, t_2 (1-octane product) = 6.3 min.

Recycling

The recycling of **3a**, **3b**, **3c** and **1** was tested under the previously conditions described above. The mixture of the reagents (1.5 M aldehyde and 4 M alkene, aldehyde:alkene 1:2.7) in acetone was transferred to the metal catalyst (1.3 mol%). Catalysis was followed by HPLC, and once the aldehyde was consumed the mixture of reagents in acetone was added again.

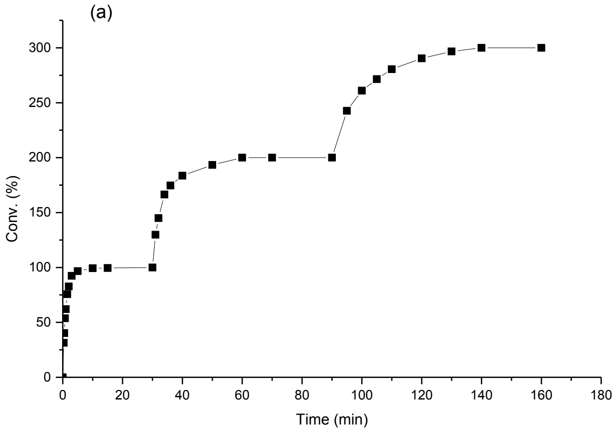

Figure S 7. Reaction profile for the hydroacylation reaction recycling **3a**. First cycle: 1.5 M aldehyde, 4.0 M alkene, 0.02 M of catalyst (1.3 mol %) and acetone as solvent.

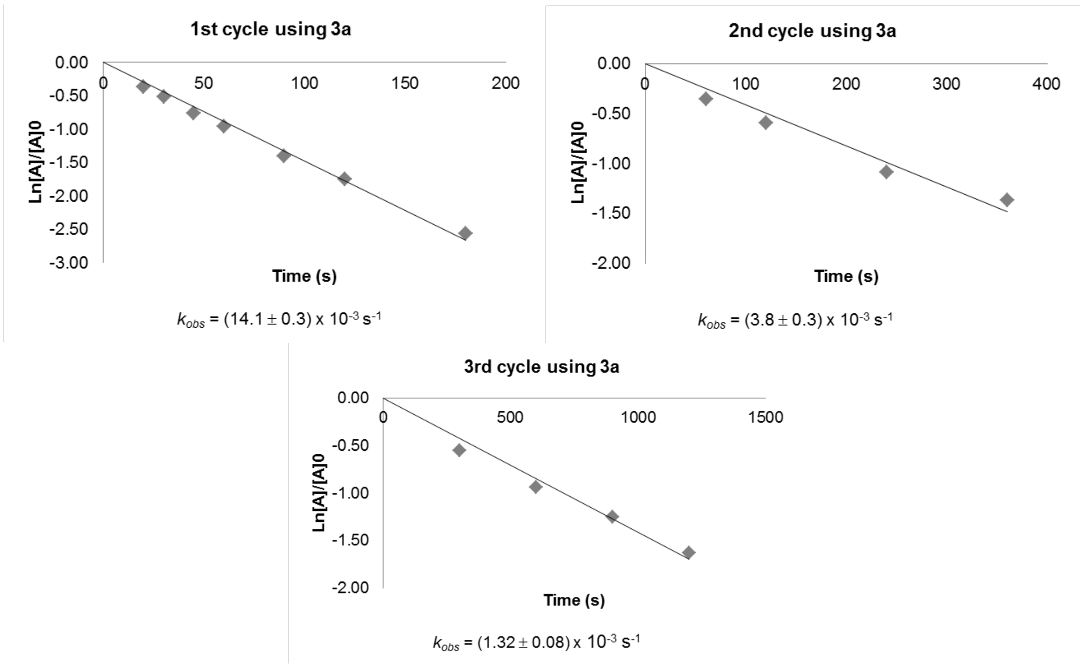

Figure S 8. Plot of Ln[A]/$[A]_0$ ([A], aldehyde concentration) during time reaction (until 95 % of conversion) using **3a**.

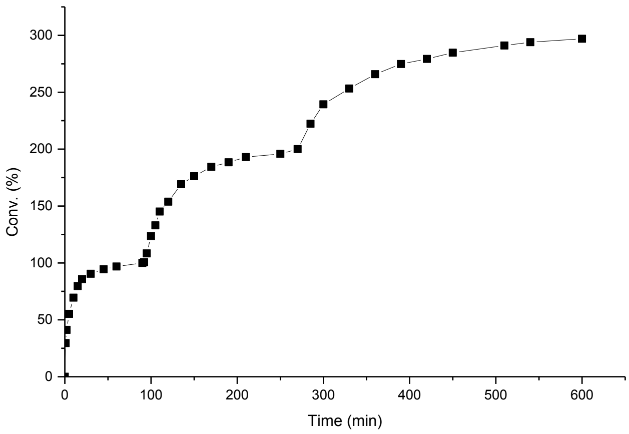

Figure S 9. Reaction profile for the hydroacylation reaction recycling **3b**. First cycle: 1.5 M aldehyde, 4.0 M alkene, 0.02 M of catalyst (1.3 mol %) and acetone as solvent.

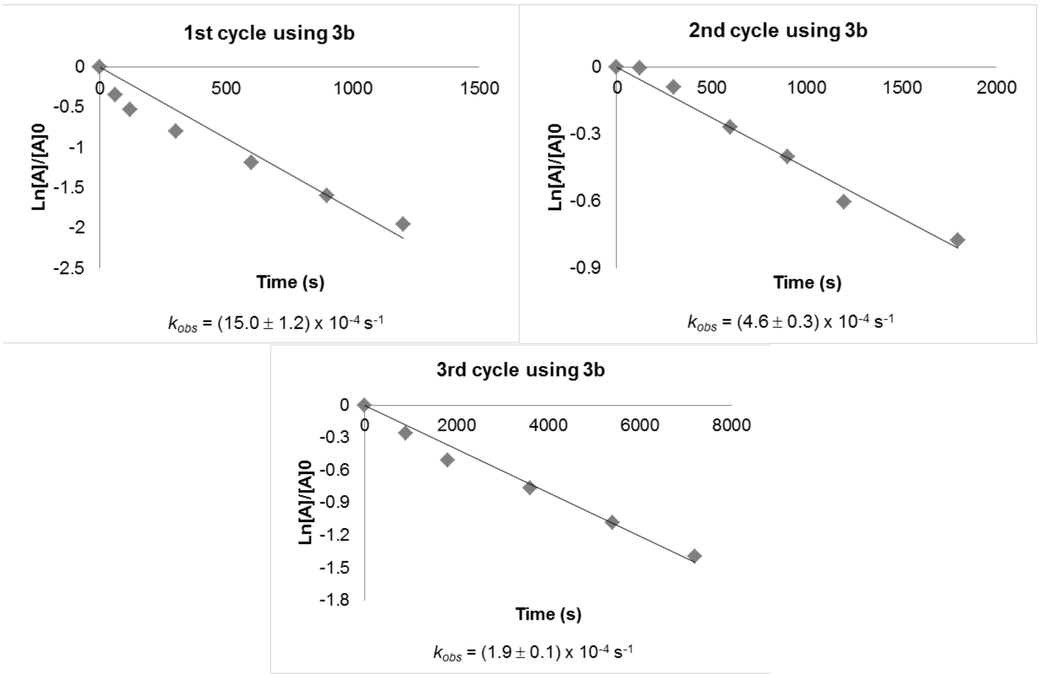

Figure S 10. Plot of Ln[A]/[A]$_0$ ([A], aldehyde concentration) during time reaction (until 95 % of conversion) using **3b**.

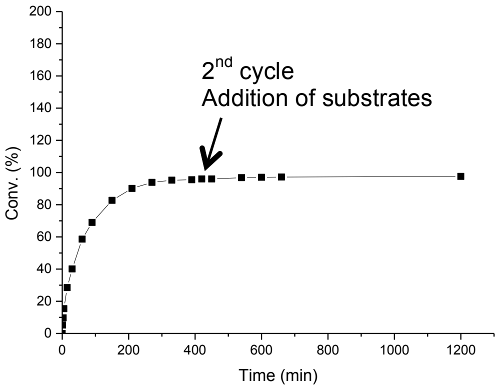

Figure S 11. Reaction profile for the hydroacylation reaction recycling **3c**. First cycle1.5 M aldehyde, 4.0 M alkene, 0.02 M of catalyst (1.3 mol %) and acetone as solvent.

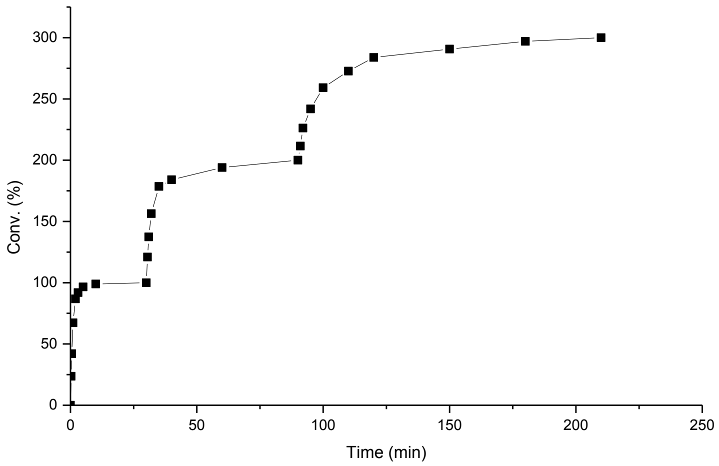

Figure S 12. Reaction profile for the hydroacylation reaction recycling **1**. First cycle: 1.5 M aldehyde, 4.0 M alkene, 0.02 M of catalyst (1.3 mol %) and acetone as solvent.

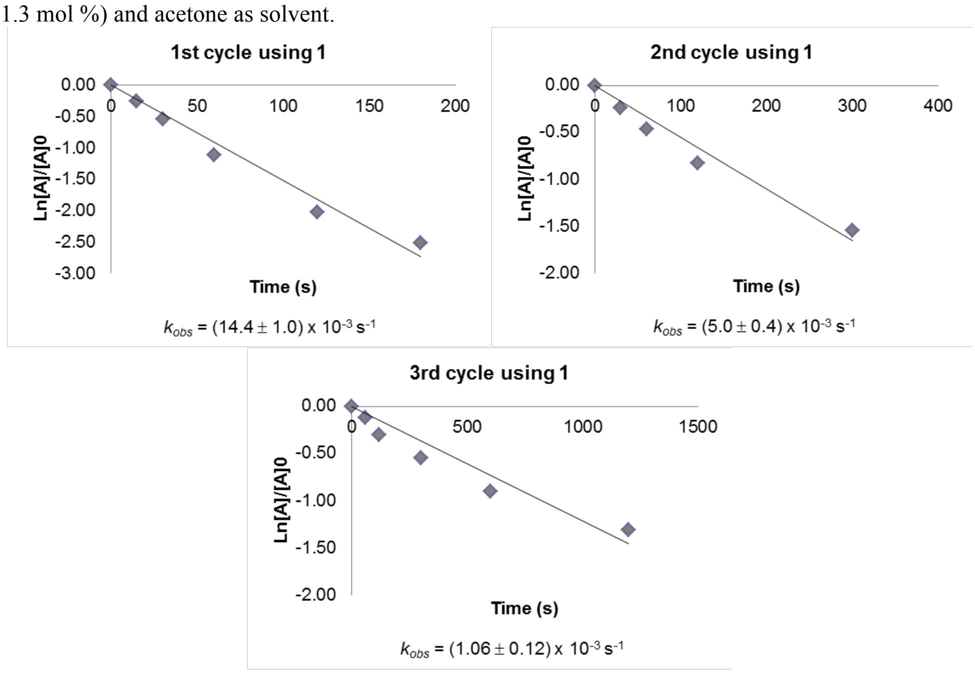

Figure S 13. Plot of Ln[A]/[A]$_0$ ([A], aldehyde concentration) during time reaction (until 95 % of conversion) using **1**.

The recycled of **3a**, **3b** and **1** was tested under the previously reported conditions (2 M aldehyde, 3 M alkene and 0.02 M catalyst in acetone, aldehyde:alkene 1:1.5). Catalysis was followed by HPLC, once the aldehyde is consumed the mixture of reagents in acetone was added again. Only **3a** was able to be recycled.

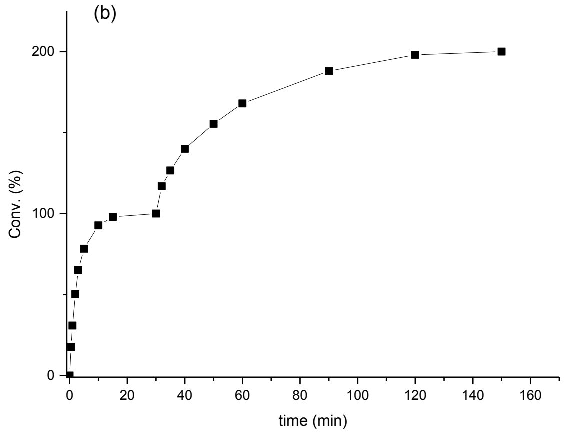

Figure S 14. Reaction profile for the hydroacylation reaction recycling **3a**. First cycle: 2.0 M aldehyde, 3.0 M alkene, 0.02 M of catalyst (1 mol %) and acetone as solvent.

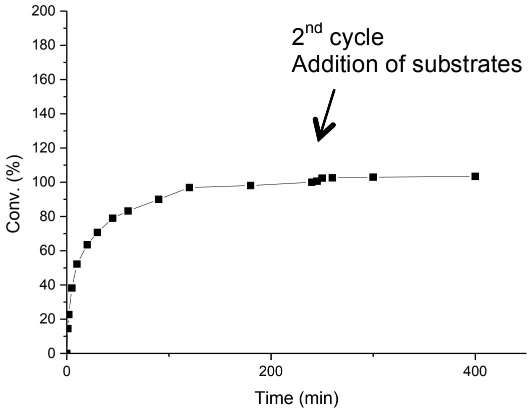

Figure S 15. Reaction profile for the hydroacylation reaction recycling **3b**. First cycle: 2.0 M aldehyde, 3.0 M alkene, 0.02 M of catalyst (1 mol %) and acetone as solvent.

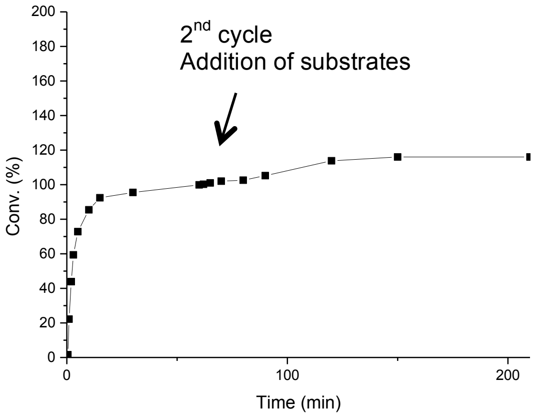

Figure S 16. Reaction profile for the hydroacylation reaction recycling **1**. First cycle: 2.0 M aldehyde, 3.0 M alkene, 0.02 M of catalyst (1 mol %) and acetone as solvent.

Hydroacylation reaction of 1-octene with 2-(methylthio)benzaldehyde was carried out adding 1 equivalent of product.

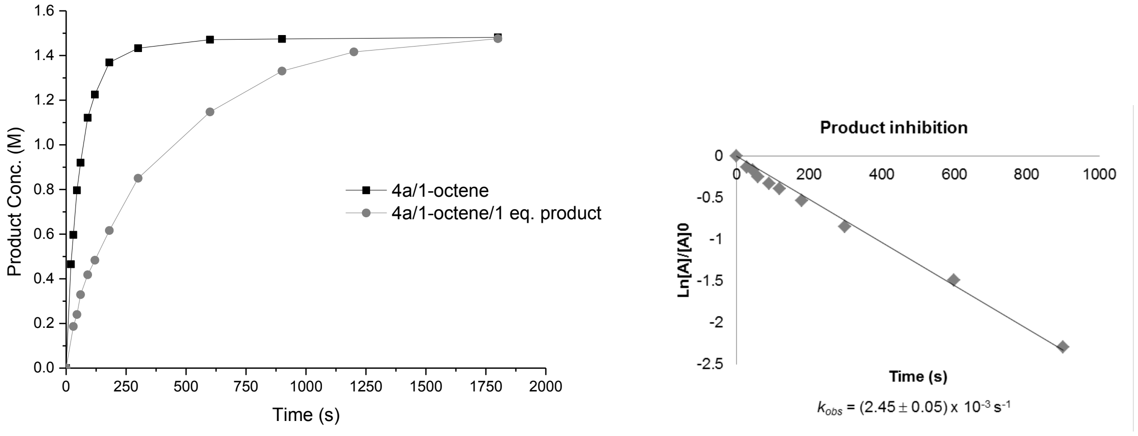

Figure S 17 Comparison of formation of product using control reaction conditions and adding 1 equivalent of product. Plot of ln[A]/$[A]_0$ ([A], aldehyde concentration) during time reaction (until 95 % of conversion) using **3a** with 1 equivalent pf product.

Kinetic studies

Hydroacylation reaction of 1-octene with 2-(methylthio)benzaldehyde was carried out fixing aldehyde concentration (1.5 M) and alkene concentration (4 M) (aldehyde:alkene 1:2.7). Different concentrations of catalyst (**3a**) were used.

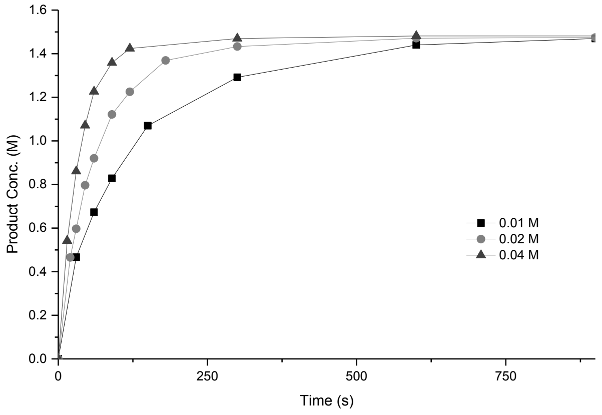

Figure S 18. Plot of Product concentrations during the catalytic hydroacylation reactions with different **3a** catalyst concentrations.

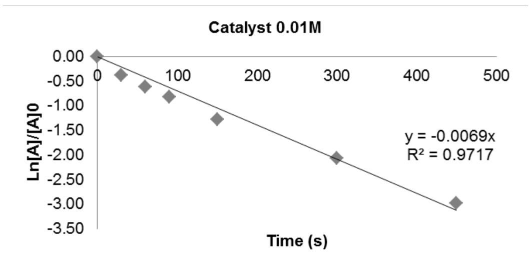

(a)

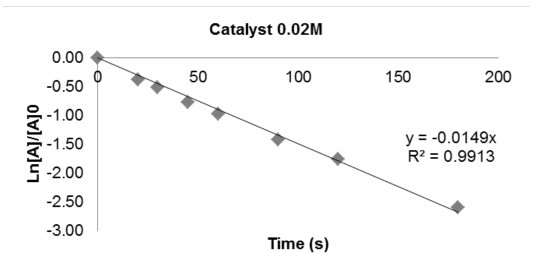

(b)

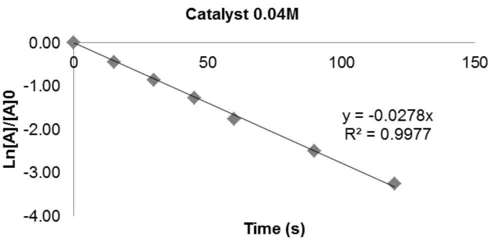

(c)

Figure S 19. Plot of Ln[A]/[A]$_0$ ([A], aldehyde concentration) during time reaction (until 95 % of conversion) with different catalyst concentrations. (a) 0.01M, (b) 0.02M and (c) 0.04M catalyst concentration.

Hydroacylation reaction of 1-octene with 2-(methylthio)benzaldehyde was carried out fixing aldehyde concentration (1.5 M) and catalyst concentration (0.02 M of **3a**). Different concentrations of alkene were used.

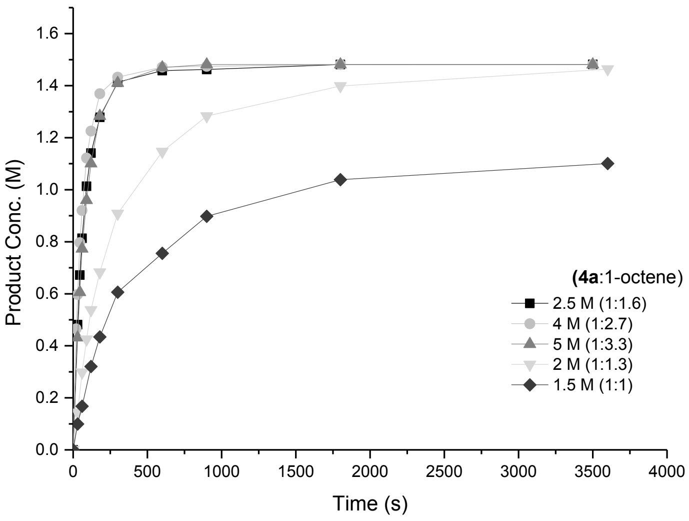

Figure S 20 Plot of Product concentration during the catalytic hydroacylation reaction with different alkene concentration.

(a)
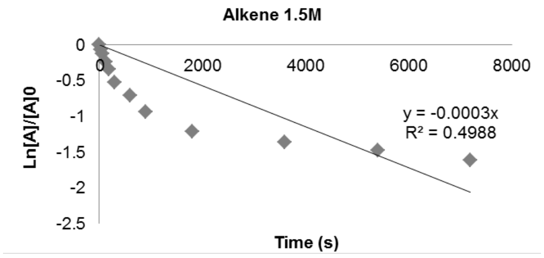

(b)
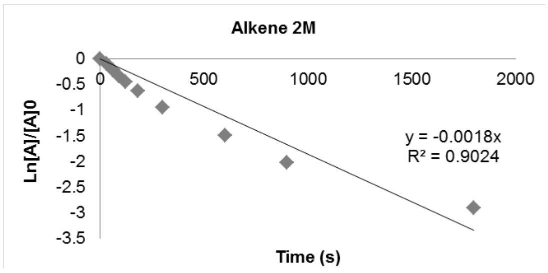

(c)
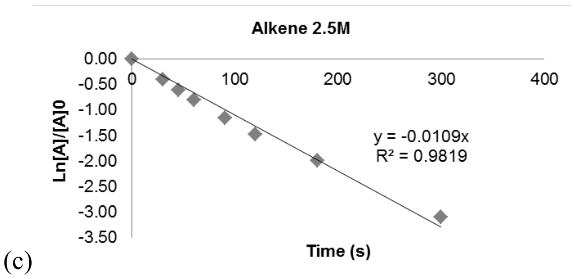

(d)
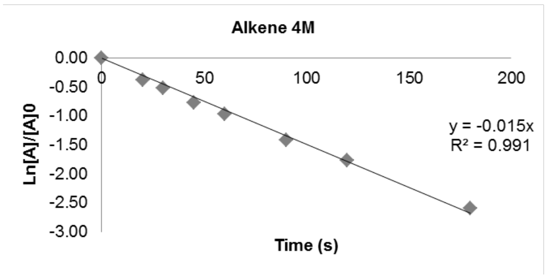

(e)
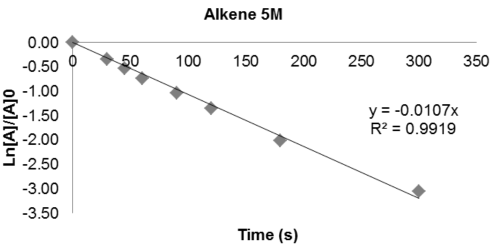

Figure S 21. Plot of Ln[A]/$[A]_0$ ([A], aldehyde concentration) during time reaction (until 95 % of conversion) with diferent alkene concentrations. (a) 1.5 M (aldehyde:alkene 1:1), (b) 2 M (aldehyde:alkene 1:1.3), (c) 2.5 M (aldehyde:alkene 1:1.6), (d) 4 M (aldehyde:alkene 1:2.7), and (e) 5 M (aldehyde:alkene 1:3.3).

Hydroacylation reaction of 1-octene with 2-(methylthio)benzaldehyde was carried out fixing alkene concentration (3 M) and catalyst concentration (0.02 M of **3a**). Different concentrations of aldehyde were used.

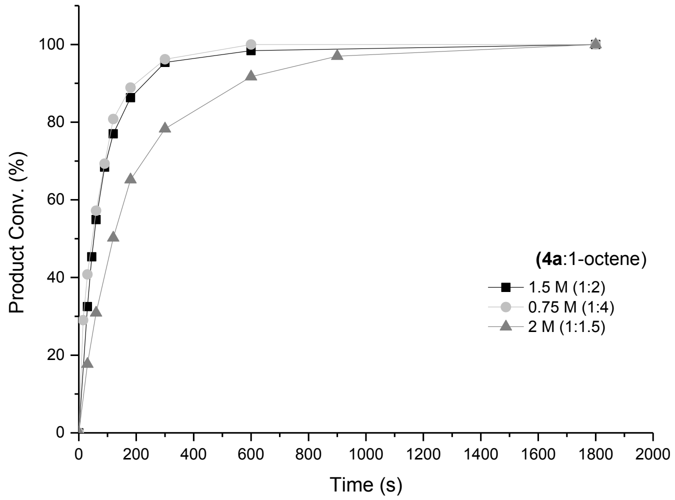

Figure S 22. Plot of Product conversions during the catalytic hydroacylation reaction with different aldehyde concentration.

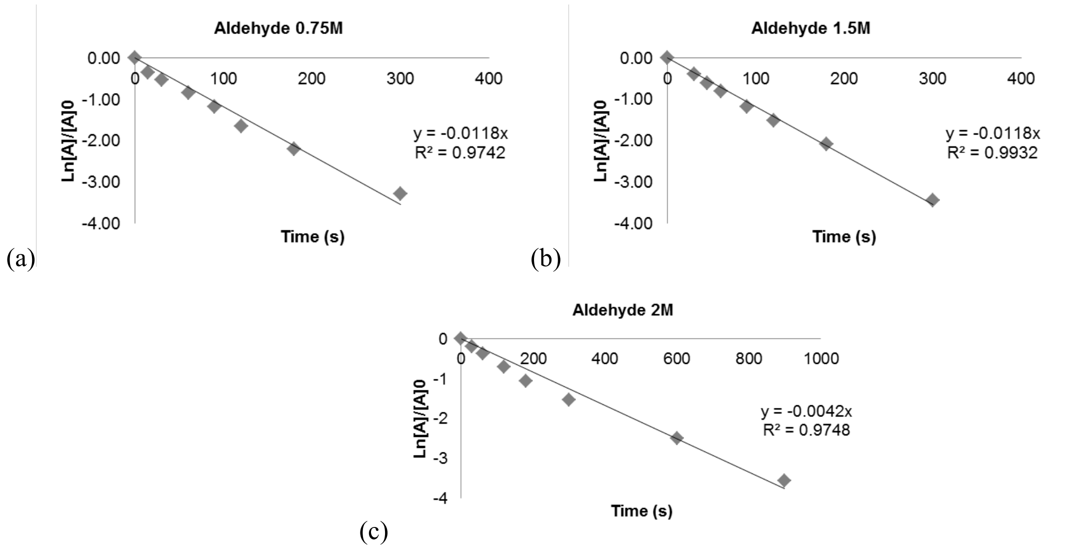

Figure S 23. Plot of Ln[A]/$[A]_0$ ([A], aldehyde concentration) during time reaction (until 95 % of conversion) with different aldehyde concentrations. (a) 0.75 M (aldehyde:alkene 1:4), (b) 1.5 M (aldehyde:alkene 1:2) and (c) 2 M (aldehyde:alkene 1:1.5).

NMR scale reactions

In a J. Youngs NMR tube **3a** and **6** were dissolved in acetone-d_6 (0.4 mL) and 1.1eq of 2-(methylthio)benzaldehyde was added. Immediately the yellow solution turns brown, over time the colour changes back to yellow (3-6 h). The reactions were followed by ^{1}H and ^{31}P{^{1}H} NMR spectroscopy.

A mixture of products is formed and hydride signals were detected in both.

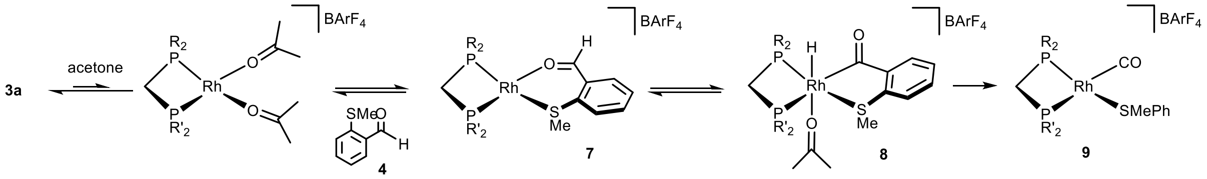

Scheme S 1. Addition of **4a** to **3a** in d_6-acetone

7: ^{1}HNMR (d_6-acetone, 500MHz, 298K, selected data): δ 9.94 ppm (br, 1H, CHO).

8: ^{1}HNMR (d_6-acetone, 500MHz, 298K, selected data): δ - 20.38 ppm (br, 1H, RhH).

8'(acetonitrile adduct): ^{1}HNMR (d_6-acetone, 500MHz, 298K, selected data): δ - 15.81 ppm (br, 1H, RhH).

9: Two different isomers are observed **9'** and **9''**.

^{1}HNMR (d_6-acetone, 400MHz, 298K, selected data): δ 4.16 (m, 2H, PCH_2P, **9'**), 4.08 (m, 2H, PCH_2P, **9''**), 3.57 (s, 6H, $CH_{3\ PhOMe}$, **9'**), 3.51 (s, 6H, $CH_{3\ PhOMe}$, **9''**), 2.69 (br, 3H, $CH_{3\ PhSMe}$, **9'**), 2.67 (br, 3H, $CH_{3\ PhSMe}$, **9''**), 1.26 (d, $^3J_{PH}$ = 16 Hz, 18H, $CH_{3\ tBu}$, **9'**) and 1.17 ppm (d, $^3J_{PH}$ = 15.5 Hz, 16H, $CH_{3\ tBu}$, **9''**).

$^{31}P\{^1H\}$ NMR (d_6-acetone, 162MHz, 298K,): δ 24.63 (br, $P(^tBu)_2$, **9'**), δ -8.1 (dd, $^1J_{RhP}$ = 77 Hz, $^2J_{PP}$ = 105 Hz, $P(^tBu)_2$, **9''**) -34.5 (br, $P(^tBu)_2$, **9''**) and -58.1 ppm (dd, $^1J_{RhP}$ = 110 Hz, $^2J_{PP}$ = 72 Hz, $P(PhOMe)_2$, **9'**).

$^{31}P\{^1H\}$ NMR (CD_2Cl_2, 202MHz, 298K,): δ 22.9 (dd, $^1J_{RhP}$ = 120 Hz, $^2J_{PP}$ = 70.5 Hz, $P(^tBu)_2$, **9'**), -8.8 (dd, $^1J_{RhP}$ = 105 Hz, $^2J_{PP}$ = 73 Hz, $P(^tBu)_2$, **9''**), -34.6 (dd, $^1J_{RhP}$ = 132 Hz, $^2J_{PP}$ = 72 Hz, $P(PhOMe)_2$, **9''**) and -30.4 ppm (dd, $^1J_{RhP}$ = 110 Hz, $^2J_{PP}$ = 70.5 Hz, $P(PhOMe)_2$, **9'**).

^{19}F NMR (CD_2Cl_2, 282 MHz, 293 K): δ **-** 62.8 ppm (s, 24F, ArF).

IR (solid): 2091.7, 2046.7 cm^{-1}

ESI-MS (CH_2Cl_2, 60°C, 4.5 kV) positive ion: m/z, 659.13 $[M]^+$ (calc. 659.14).

10:

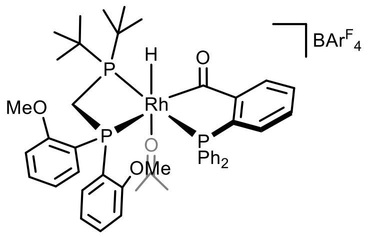

^1H NMR (CD_2Cl_2, 500MHz, 298K): δ 7.64 (br, 8H, CH_{ArF}), 7.64 (m, 2H, CH_{PhOMe}), 7.51 (m, 5H, $CH_{PhP(Ph)2}$), 7.47 (br, 4H, CH_{ArF}), 7.43 (m, 2H, CH_{PhOMe}), 7.30 (m, 5H, $CH_{PhP(Ph)2}$), 7.04 (m, 2H, $CH_{PhP(Ph)2}$), 7.01 (m, 2H, CH_{PhOMe}), 6.91 (m, 1H, $CH_{PhP(Ph)2}$), 6.80 (m, 2H, CH_{PhOMe}), 6.51 (m, 1H, $CH_{PhP(Ph)2}$), 3.84 (m, 1H, PCH_2P), 3.23 (m, 1H, PCH_2P), 3.18 (s, 3H, $CH_{3\ PhOMe}$), 3.05 (s, 3H, $CH_{3\ PhOMe}$), 2.03 (s, 6H, $CH_{3\ (CH3)2CO}$), 1.36 ppm (d, $^3J_{PH}$ = 15 Hz, 9H, $CH_{3\ tBu}$), 1.23 (d, $^3J_{PH}$ = 15 Hz, 9H, $CH_{3\ tBu}$) and -19.59 ppm (app. dq, $^1J_{RhH}$ = 34 Hz, $^2J_{PH}$ = 8 Hz, 1H, RhH).

^{31}P NMR (CD_2Cl_2, 202MHz, 298K): δ 64.0 (ddd, $^1J_{RhP}$ = 127 Hz, $^2J_{PP}$ = 312 Hz, $^2J_{PP}$ = 20 Hz, $P(Ph)_2$), 30.1 (ddd, $^1J_{RhP}$ = 108 Hz, $^2J_{PP}$ = 312 Hz, $^2J_{PP}$ = 13, $P(^tBu)_2$) and -30.0 ppm (d, $^1J_{RhP}$ = 64 Hz, $P(PhOMe)_2$).

^{19}F NMR (CD_2Cl_2, 282 MHz, 293 K): δ **-** 62.8 ppm (s, 24F, ArF).

ESI-MS (CH_2Cl_2, 60°C, 4.5 kV) positive ion: m/z, 797.67 $[M-C_3H_6O]^+$ (calc. 797.68).

11:

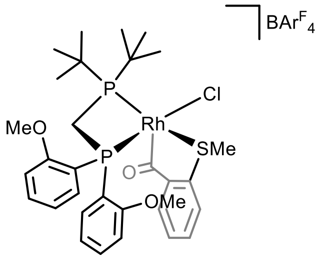

^1H NMR (CD_2Cl_2, 500MHz, 298K): δ 7.73-7.68 (m, 2H, CH_{PhOMe}), 7.63 (br, 8H, CH_{ArF}), 7.55 (m, 2H, CH_{PhOMe}), 7.47 (br, 4H, CH_{ArF}), 7.41-7.37 (m, 1H, CH_{PhSMe}), 7.25 (m, 1H, CH_{PhSMe}), 7.17 (m, 2H,

CH_{PhOMe}), 7.03-6.68 (m, 2H, CH_{PhOMe}), 6.84 (m, 1H, CH_{PhSMe}), 6.25 (m, 1H, CH_{PhSMe}), 4.54 (m, 1H, PCH_2P), 3.66 (m, 1H, PCH_2P), 3.56 (s, 3H, $CH_{3\ PhOMe}$), 3.00 (d, 3H, $^3J_{RhH}$ = 5 Hz, $CH_{3\ PhSMe}$), 2.87 (s, 3H, $CH_{3\ PhOMe}$), 1.52 ppm (d, $^3J_{PH}$ = 20 Hz, 9H, $CH_{3\ tBu}$) and 1.34 ppm (d, $^3J_{PH}$ = 20 Hz, 9H, $CH_{3\ tBu}$).
^{31}P NMR (CD_2Cl_2, 202MHz, 298K): δ -7.2 (dd, $^1J_{RhP}$ = 120 Hz, $^2J_{PP}$ = 54 Hz, $P(^tBu)_2$) and -47.1 ppm (dd, $^1J_{RhP}$ = 117 Hz, $^2J_{PP}$ = 56 Hz, $P(PhOMe)_2$).
^{19}F NMR (CD_2Cl_2, 282 MHz, 293 K): δ **-** 62.8 ppm (s, 24F, ArF).
ESI-MS (CH_2Cl_2, 60°C, 4.5 kV) positive ion: m/z, 693.1001 $[M]^+$ (calc. 693.0990).

12:

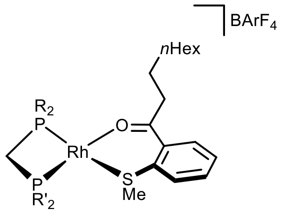

Two different isomers are observed **12'** (32%) and **12''**(68%).
^{1}HNMR (d_6-acetone, 202MHz, 220K, selected data): δ 3.79 br, 2H, PCH_2P, **12''**), 3.70 (s, 6H, $CH_{3\ PhOMe}$, **12'**), 3.64 (s, 3H, $CH_{3\ PhOMe}$, **12''**), 3.45 (m, 2H, PCH_2P, **12'**), 3.37 (s, 3H, $CH_{3\ PhOMe}$, **12''**), 2.62 (br, 3H, $CH_{3\ PhSMe}$, **12'**) and 2.49 ppm (br, 3H, $CH_{3\ PhSMe}$, **12''**).
^{31}P{^{1}H} NMR (d_6-acetone, 202MHz, 298K,): δ 28.5 (br, $P(^tBu)_2$, **12'**), δ 9.6 (br, $P(^tBu)_2$, **12''**) -28.3 (br, $P(PhOMe)_2$, **12''**) and -47.5 ppm (br, $P(PhOMe)_2$, **12'**),
^{31}P{^{1}H} NMR (d_6-acetone, 202MHz, 220K): δ 27.8 (dd, $^1J_{RhP}$ = 156 Hz, $^2J_{PP}$ = 101 Hz, $P(^tBu)_2$, **12'**), 9.1 (dd, $^1J_{RhP}$ = 143.4 Hz, $^2J_{PP}$ = 103 Hz, $P(^tBu)_2$, **12''**), -49.5 (dd, $^1J_{RhP}$ = 153 Hz, $^2J_{PP}$ = 103 Hz, $P(PhOMe)_2$, **12'**) and -30.4 ppm (dd, $^1J_{RhP}$ = 170 Hz, $^2J_{PP}$ = 103 Hz, $P(PhOMe)_2$, **12''**).
ESI-MS (CH_2Cl_2, 60°C, 4.5 kV) positive ion: m/z, 771.267 $[M]^+$ (calc. 771.26).

Labelling Studies

Determination of the kinetic isotopic effect (KIE) for the hydroacylation reaction.

Two sets of reactions were carried out on side by side samples, 2-(methylthio)benzaldehyde (**4a**) and d1-2-(methylthio)benzaldehyde (d-**4a**) were reacted with 1-octene under the optimized conditions (1.5 M aldehyde, 4 M alkene and 0.02 M catalyst, aldehyde:alkene 1:2.7). At the desired times, aliquots were extracted from the reaction, quenched by dilution with 1.5 mL of acetonitrile and analysed by HPLC.

The reaction of d1-2-(methylthio)benzaldehyde with 1-octene was also followed by NMR detecting an small signal of non deuterated 2-(methylthio)benzaldehyde.

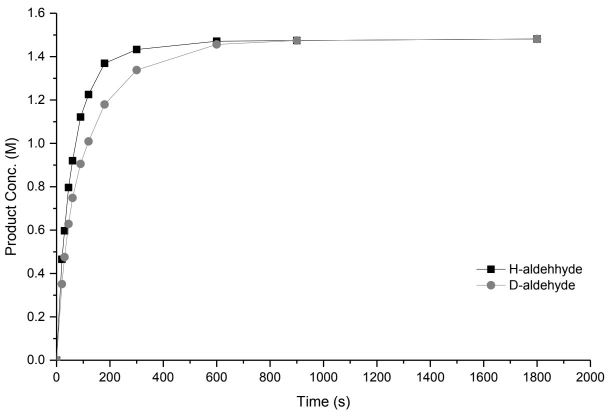

Figure S 24 Comparison of conversions using deuterated aldehyde with respect to the non-deuterated control reaction.

The products were isolated by column chromatography eluted in 5% Et_2O/petrol, to give the products as colourless oil (**Figure S 25** and **Figure S 26**). Analysis of the ^1H NMR and ^2H NMR spectra indicates a 40% incorporation of deuterium at the α position and 60% in the β position. (**Figure S 26** and **Figure S 27**).

The catalytic reaction was carried out using deuterated 2-(methylthio)benzaldehyde (d-**4a**) and 2,3-dihydrofurane under the same conditions. The product was isolated by column chromatography eluted in 5% Et_2O/petrol, to give the product as colourless oil. Analysis of the ^2H NMR spectra indicates deuterium incorporation only in the β position. (**Figure S 28** and **Figure S 29**). The reactions was also followed by NMR, at 60% of conversion incorporation of H was observed into the aldehyde (**Figure S 30**).

The catalytic reaction was slower using deuterated 2-(methylthio)benzaldehyde (d-**4a**) and methylcrotonate under the same conditions. The reaction was stopped after 60% of conversion and the mixture of aldehyde and product isolated, an small signal of non deuterated 2-(methylthio)benzaldehyde is detected by ^1H NMR (**Figure S 31**) and the ^2H NMR spectra indicates deuterium incorporation only in the β position of the product. (**Figure S 32**).

The catalytic reaction using 2-(methylthio)benzaldehyde and 1-octene under the same conditions did not work however the ^2H NMR spectra indicates deuterium incorporation to the free octane (**Figure S 33**) and non-deuterated aldehyde was isolated.

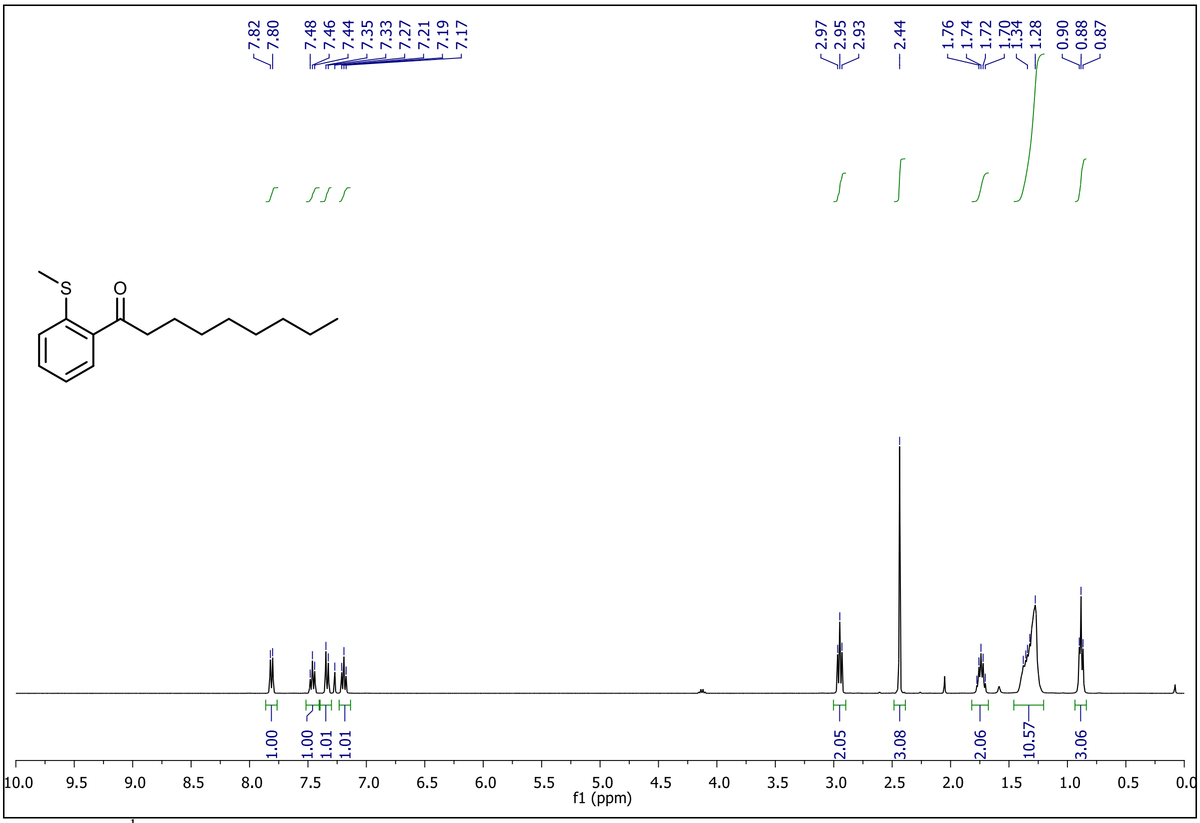

Figure S 25. ^{1}H NMR (500 MHz, $CDCl_3$) of HA product using 2-(methylthio)benzaldehyde and 1-octene.

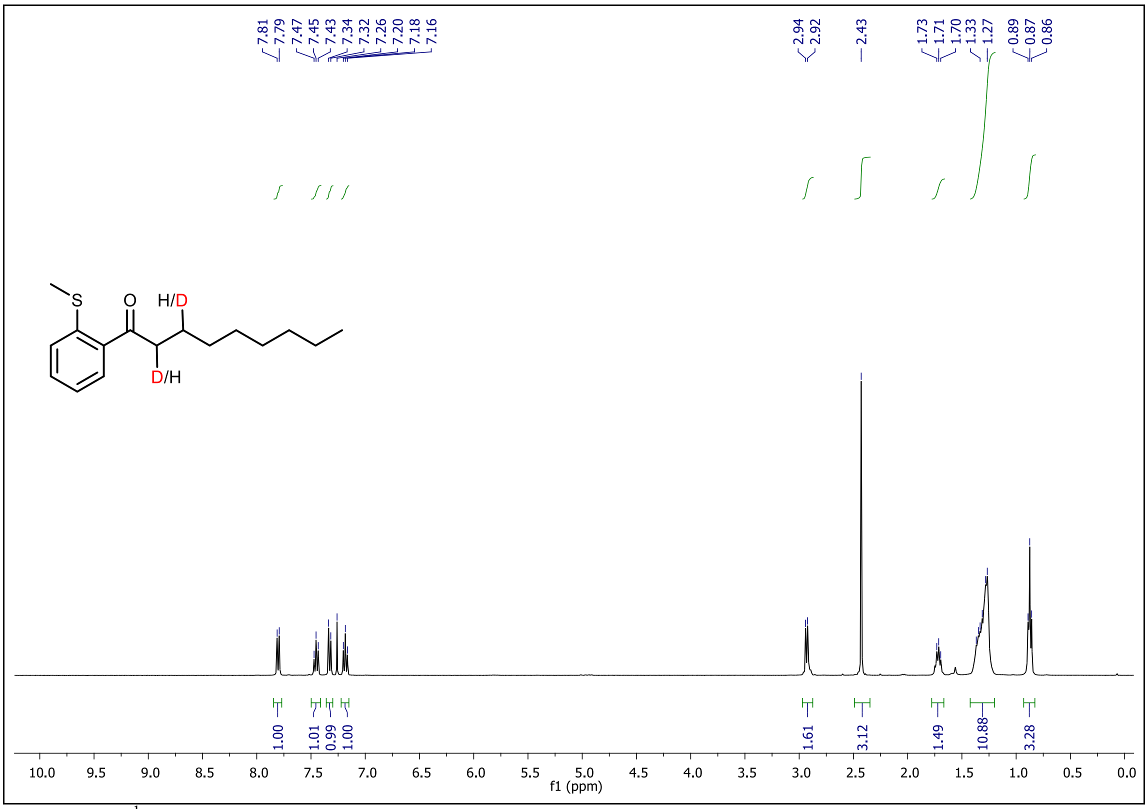

Figure S 26. ^{1}H NMR (500 MHz, $CDCl_3$) of HA product using d1-2-(methylthio)benzaldehyde and 1-octene.

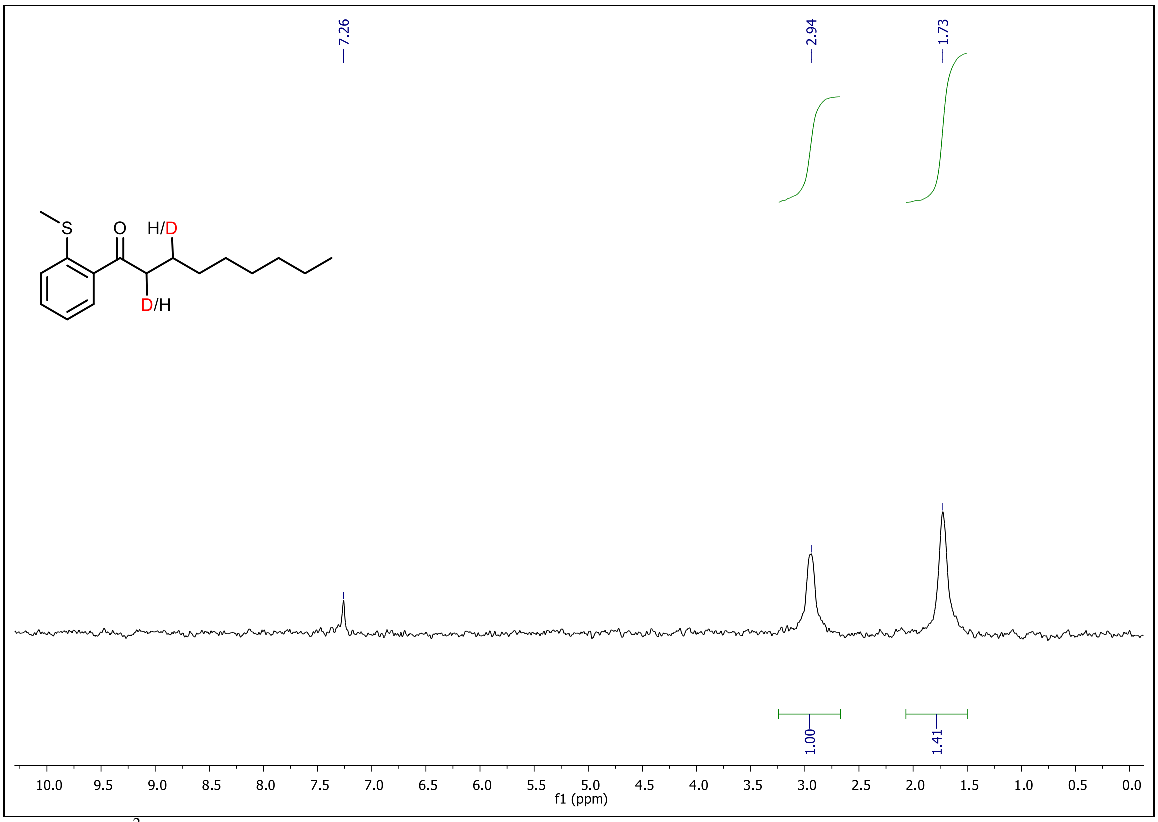

Figure S 27. ^{2}H NMR (500 MHz, $CDCl_3$/$CHCl_3$) of HA product using d1-2-(methylthio)benzaldehyde and 1-octene.

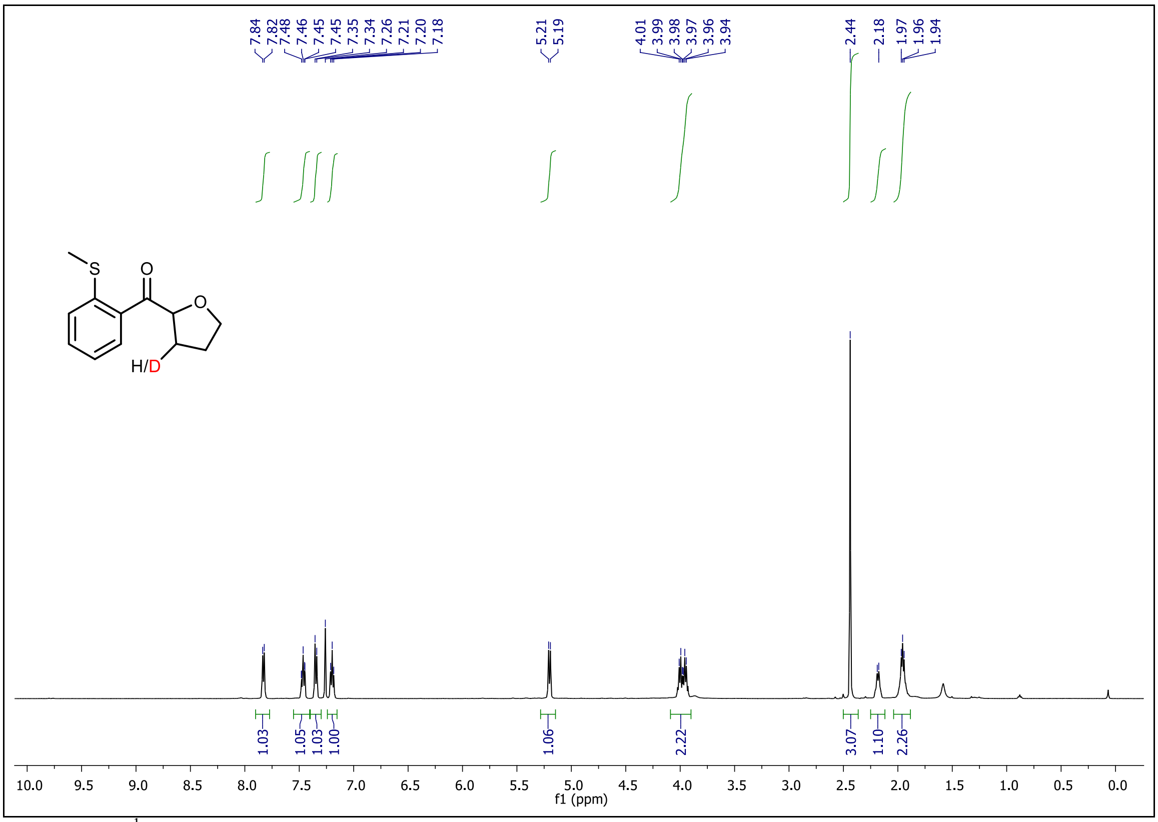

Figure S 28. ^{1}H NMR (500 MHz, $CDCl_3$) of HA product using d1-2-(methylthio)benzaldehyde and 2,3-dihydrofurane.

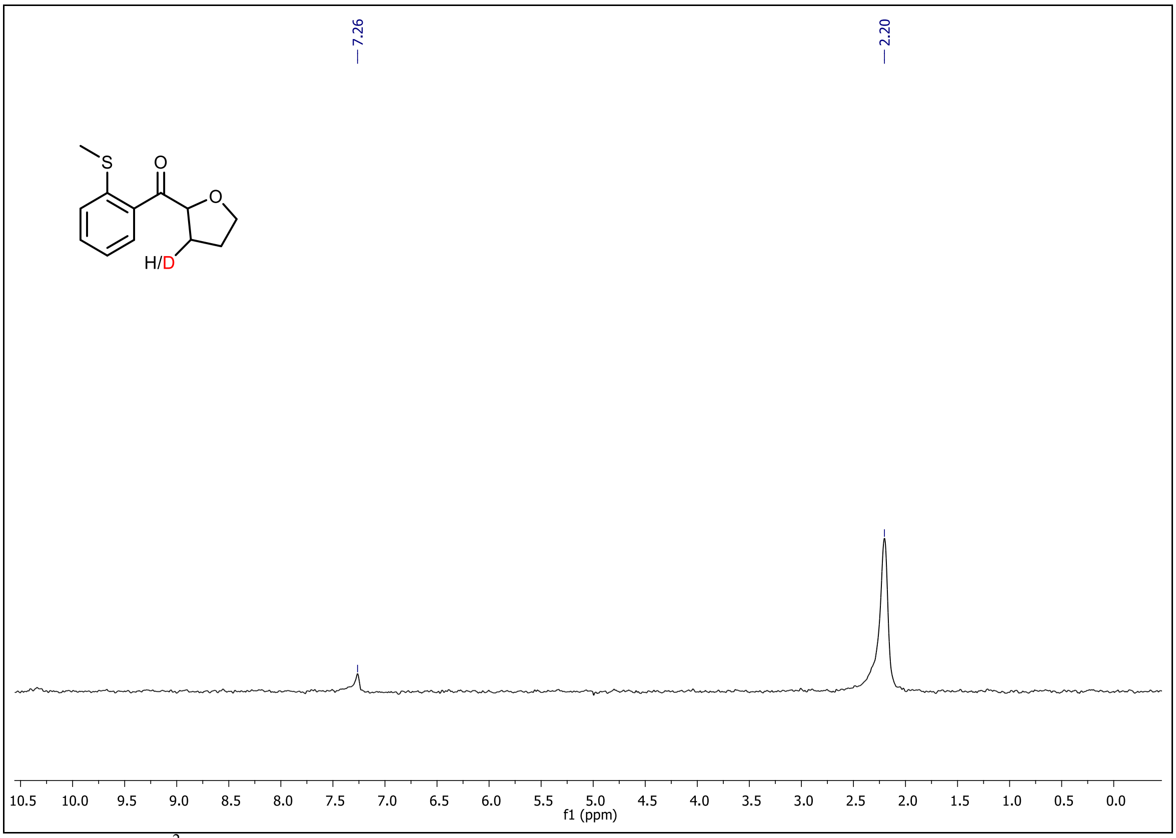

Figure S 29. ^{2}H NMR (500 MHz, $CDCl_3/CHCl_3$) of HA product using d1-2-(methylthio)benzaldehyde and 2,3-dihydrofurane.

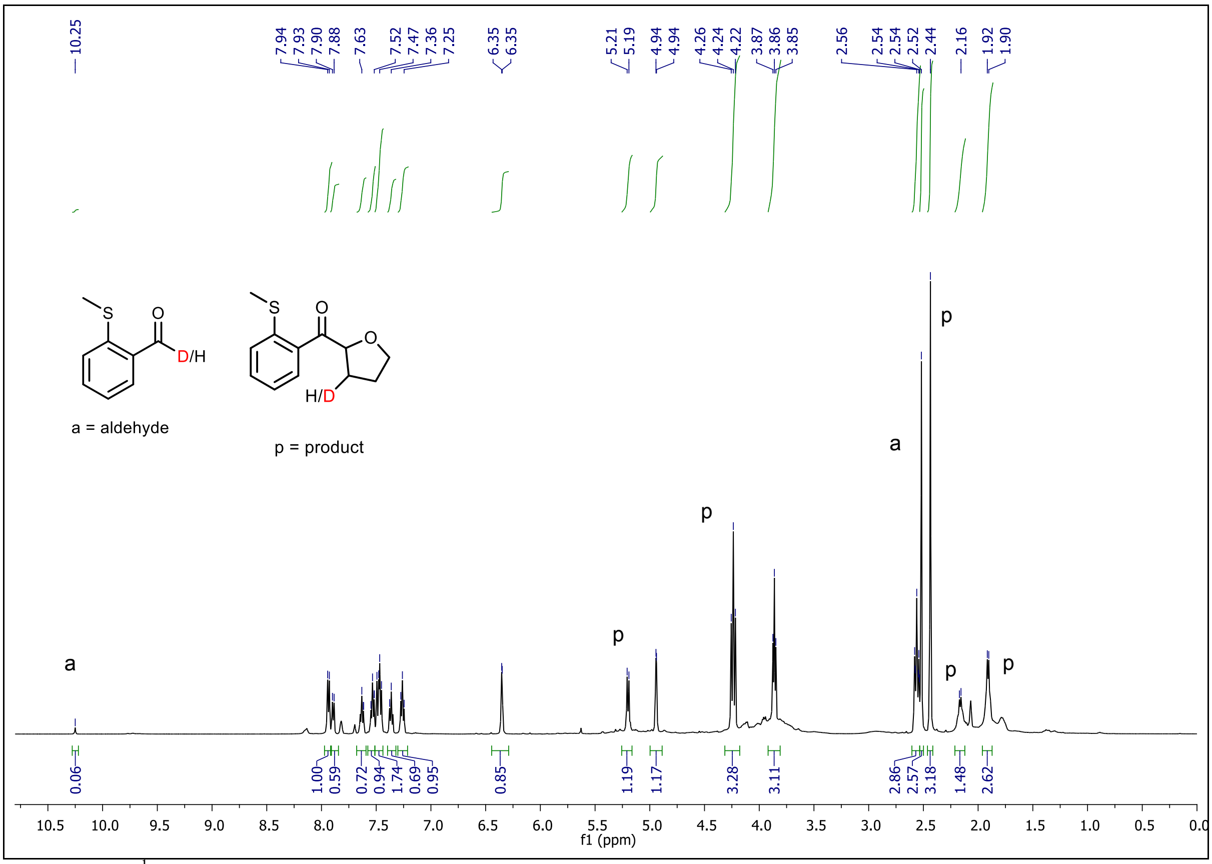

Figure S 30. ^{1}H NMR (500 MHz, $(CD_3)_2CO$) for the reaction mixture after 20 minutes (60% of conversion) using d1-2-(methylthio)benzaldehyde and methylcrotonate dihydrofurane.

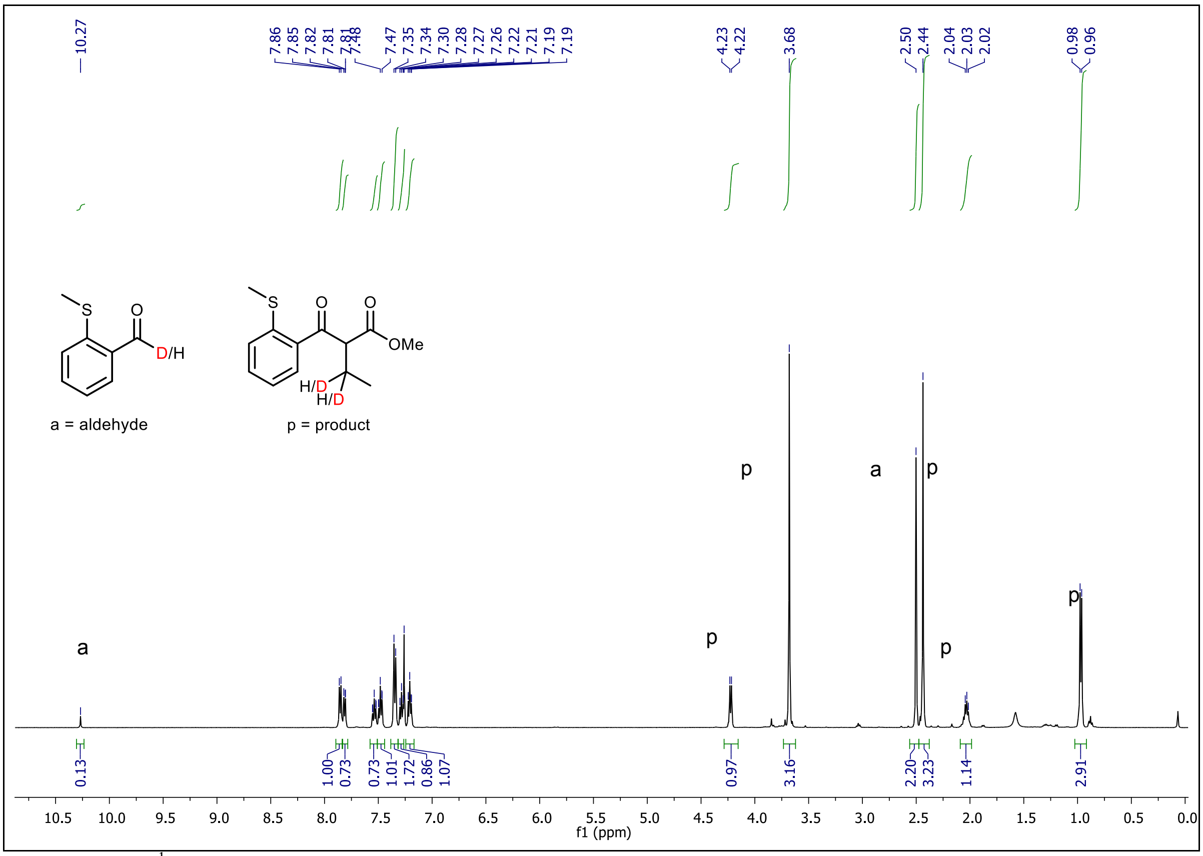

Figure S 31. ^{1}H NMR (500 MHz, $CDCl_3$) of reaction mixture after 10 hours (60% of conversion) using d1-2-(methylthio)benzaldehyde and methylcrotonate.

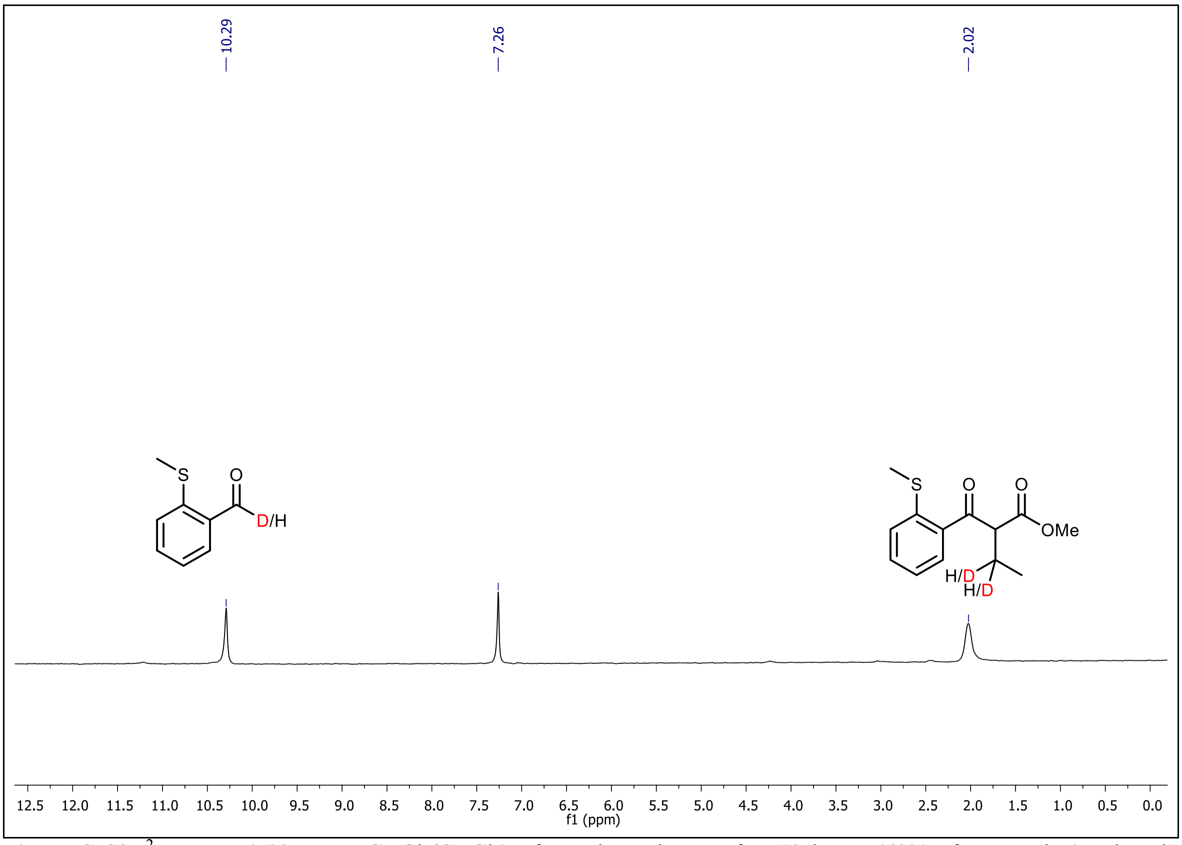

Figure S 32. ^{2}H NMR (500 MHz, $CDCl_3/CHCl_3$) of reaction mixture after 10 hours (60% of conversion) using d1-2-(methylthio)benzaldehyde and methylcrotonate.

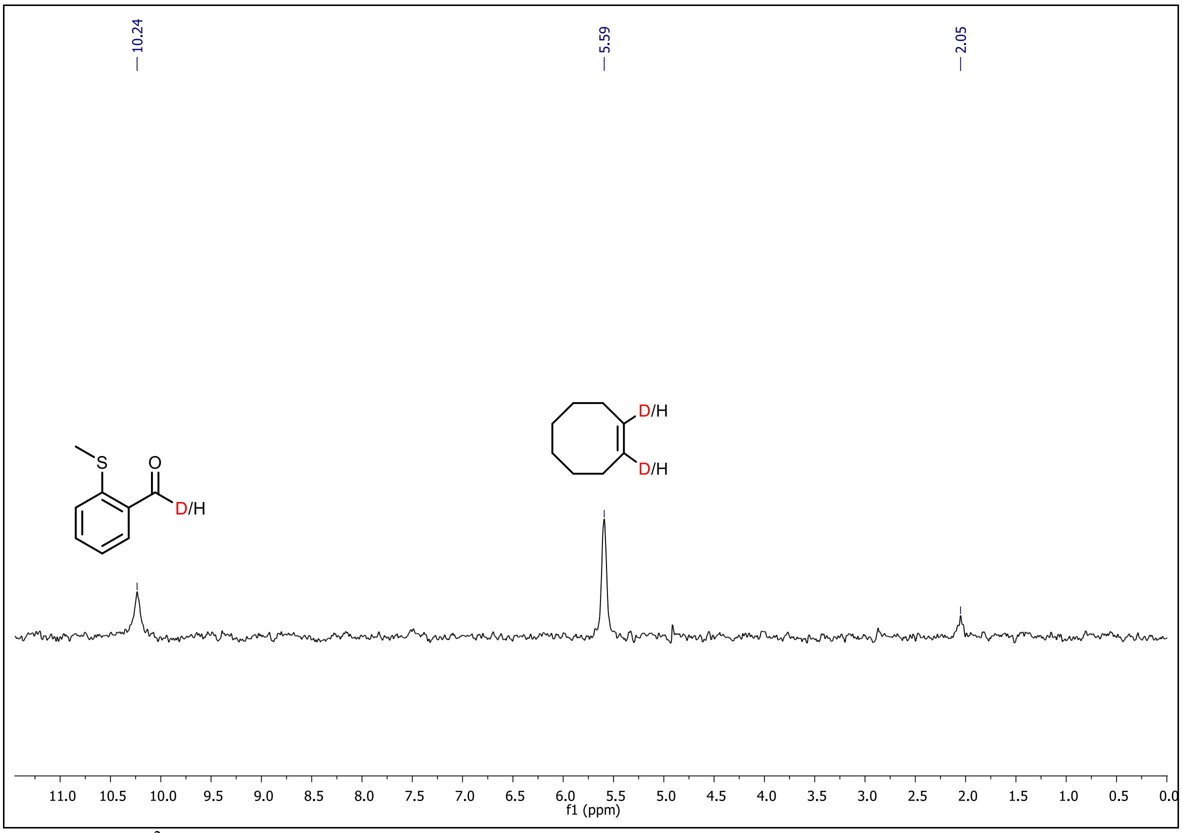

Figure S 33. ^{2}H NMR (500 MHz, $CDCl_3/CHCl_3$) of reaction mixture after 10 hours d1-2-(methylthio)benzaldehyde and cyclooctene.

Crystallographic Supporting Information

X-ray crystallography data for **3a** was collected on an Enraf Nonius Kappa CCD diffractometer using graphite monochromated Mo Kα radiation (λ = 0.71073 Å) and a low-temperature device [150(2) K];[7] data were collected using COLLECT, reduction and cell refinement was performed using DENZO/SCALEPACK.[8] The structure was solved by direct methods using SIR2004[9] and refined full-matrix least squares on F^2 using SHELXL-97.[10] X-ray crystallography data for **3d** and **11** were collected on an Agilent SuperNova diffractometer using graphite monochromated Cu Kα radiation (λ = 1.54180 Å) and a low-temperature device [150(2) K];[7] data were collected using SuperNova, reduction and cell refinement was performed using CrysAlis. The structure was solved by direct methods using Superflip and refined full-matrix least squares on F^2 using CRYSTALS. All non-hydrogen atoms were refined with anisotropic displacement parameters. All hydrogen atoms were placed in calculated positions using the riding model. Crystallographic data have been deposited with the Cambridge Crystallographic Data Centre under **CCDC** 1045333-1045335. These data can be obtained free of charge from The Cambridge Crystallographic Data Centre via www.ccdc.cam.ac.uk/data_request/cif.

Special refinement details

3d

One of the CF_3 groups upon the anion was modelled over three positions and another modelled over two. These disorder models were restrained to maintain sensible geometries. The fluorine atom of the fluorobenzene ligand is disordered over two sites around the arene ring. The occupancy of the disordered fluorines and the corresponding hydrogen atoms was refined and restraints were used to maintain symmetry within the disordered fluorobenzene unit.

3a

Disorder of the fluorobenzene ligand was treated by modelling it over two sites and restraining the 1,2 and 1,3 C-F bond distances. A rigid body constraint was applied to the arene moiety. Disorder of the solvent fluorobenzene molecule was treated similarly. Rotational disorder of the CF_3 groups of the anion was treated by modelling the fluorine atoms over two sites and restraining their geometry. Restraints to thermal parameters were applied where necessary in order to maintain sensible values.

11

Several of the CF_3 groups upon the anion were modelled over two positions and restrained to maintain sensible geometries. A disordered CH_2Cl_2 molecule was located, this was modelled over two sites and restrained to maintain sensible geometries. The minor disorder component of the CH_2Cl_2 includes fairly large displacement ellipsoids even after refinement suggesting further minor disorder of the solvent molecule may be present.

**Compound**	**3d**	**3a**	**11**
CCDC No.	1045333	1045334	1045335
Formula	$C_{59}H_{47}BF_{25}O_2P_2Rh$	$C_{61}H_{51}BF_{25}O_2P_2Rh$	$C_{63}H_{53}BF_{24}ClO_3P_2SRh$
M	1438.63	1466.21	1556.16
Crystal System	monoclinic	triclinic	triclinic
Space Group	*C* 2/c	*P* -1	*P* -1
T [K]	150(2)	150(2)	150(2)
a [Å]	18.4102(2)	13.31090(10)	12.0492(3)
b [Å]	17.59900(10)	13.84500(10)	16.1404(4)
c [Å]	37.4931(3)	18.53920(10)	19.6771(5)
α [deg]	90	90.1592(4)	71.209(2)
β [deg]	95.0512(9)	92.9715(4)	72.800(2)
γ [deg]	90	98.2490(5)	81.976(2)
V [$Å^3$]	12100.63(18)	3376.50(4)	3456.63(16)
Z	8	2	2
Radiation Type	Cu Kα	Mo Kα	Cu Kα
μ (mm^{-1})	3.867	0.416	4.770
θ range [deg]	$3.481 \leq \theta \leq 76.414$	$5.097 \leq \theta \leq 29.575$	$2.895 \leq \theta \leq 75.324$
Reflns collected	59386	27138	25181
R_{int}	0.025	0.0229	0.016
No. of data/restr/param	12511 / 834 / 901	15999 / 1086 / 1092	13737 / 980 / 1027
R_1 [I>2σ(I)	0.0413	0.0447	0.0396
wR_2 [all data]	0.1010	0.1192	0.1009
GoF	1.0835	1.027	0.9918
Largest diff. pk and hole [e/$Å^3$]	1.22, -1.04	0.692, -0.699	1.63, -1.01

Bond distances (Å) and angles (°)

**Compound**	**3d**	**3a**	**11**
Rh1-Px (R = Alkyl)	2.2228(7) (x=1)	2.2604(7) (x=1)	2.3266(7) (x=2)
Rh1-Px (R = Aryl)	2.2370(7) (x=2)	2.2318(8) (x=2)	2.2970(6) (x=1)
Rh1-(C1-6)range	2.336(2) - 2.278(3)	2.355(3) - 2.278(3)	
Rh1-C8			1.986(3)
Rh1-S1			2.4006(7)
Rh1-Cl1			2.3690(7)
P1-Rh1-P2	72.64(3)	72.83(3)	72.97(2)
S1-Rh1-C8			85.91(9)
S1-Rh1-Cl1			90.93(3)

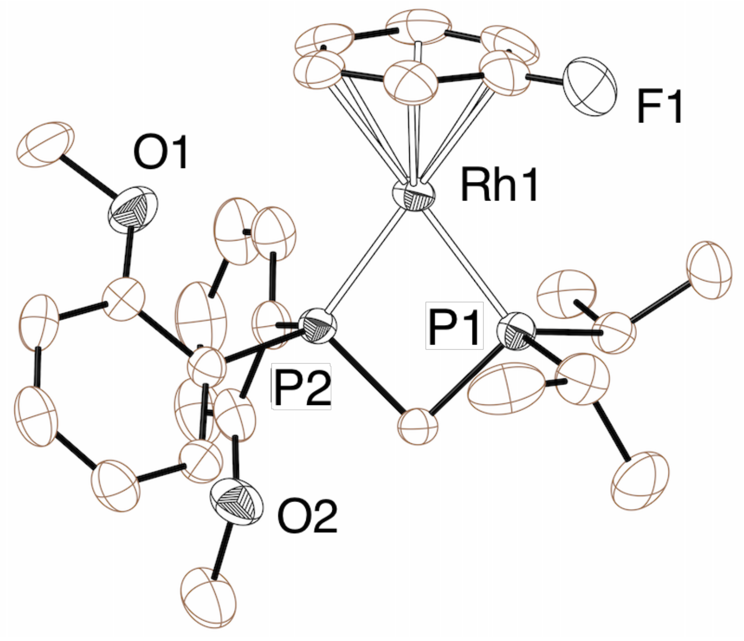

Figure S34 Solid–State structure of complex **3d**. Displacement ellipsoids are shown at the 30% probability level.

Experimental – Organic synthesis

General experimental methods

Reactions were performed under inert atmosphere of nitrogen with anhydrous solvent unless otherwise stated. All glassware was oven dried at >80 °C, and allowed to cool to room temperature under a positive nitrogen pressure. Reactions were monitored by TLC until deemed complete using aluminum backed silica plates. Plates were visualized under ultraviolet light and/or by staining with phosphomolibdic acid or Seebach's stain (Magic).

Reagents were purchased from Sigma-Aldrich Chemical Co. Ltd., Alfa Aesar, Acros Organics Ltd., Lancaster Synthesis Ltd, or Strem Chemicals Inc. and were used as supplied. 2-(Methylthio)benzaldehyde **4a** was purchased from Sigma-Aldrich and purified by flash chromatography (1:1 petrol/dicloromethane) and distilled (145 °C, 13 mmHg) prior to use. Acetone was distilled from Drierite®. Dichloroethane was distilled from calcium hydride. Petrol refers to the fractions obtained between 40 and 60 °C. Ether refers to diethyl ether. Flash chromatography was carried out using matrix 60 silica.

^{1}H NMR spectra were obtained on a Bruker AVIII400 (400 MHz) spectrometer using the residual solvent as an internal standard. ^{13}C NMR spectra were obtained on a Bruker AVIII400 (100 MHz) spectrometer using the residual solvent as an internal standard. Chemical shifts were reported in parts per million (ppm) with the multiplicities of the spectra reported as following: s, singlet; d, doublet; t, triplet; q, quartet; m, multiplet; br, broad. Low resolution ESI mass spectra were recorded on a Waters LCT Premier spectrometer. High resolution ESI mass spectrometry measurements were recorded on a Brucker Daltronics microTOF (ESI) spectrometer by the internal service at the Department of Organic Chemistry, University of Oxford. Infra-red spectra were recorded as thin films on a Bruker Tensor 27 FT-IR spectrometer. Melting points were determined using a Stuart Scientific Melting Point Apparatus SMP1.

Synthesis of new compounds

General procedure

To an oven dried reaction flask containing a magnetic stirrer was added catalyst **3a** (1-5 mol%) in the glovebox. The reaction flask was taken out of the glovebox and the catalyst was dissolved in acetone (150 µL), followed by the addition of 2-(methylthio)benzaldehyde **4a** (0.3 mmol, 1 equiv.) and the corresponding alkene (0.45 mmol, 1.5 equiv). The resulting solution was heated at 55 °C until completion (up to 3 h), and then allowed to cool to room temperature. The crude was directly charged onto silica gel and subjected to flash column chromatographical purification (FC) to afford the corresponding pure product.

Methyl 2-methyl-4-[2-(methylthio)phenyl]-4-oxobutanoate (Table 2, Entry 1)

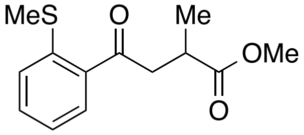

Following the general procedure the product (108 mg, 0.43 mmol) was isolated by FC (petrol/ether 8:2) in 95% yield as a clear oil, starting from 2-(methylthio)benzaldehyde (58 µL, 0.45 mmol) and methyl metacrilate (75 µL, 0.68 mmol) in the presence of **3a** (20 mg, 0.013 mmol, 3 mol%) and using acetone (225 mL) as solvent. **^{1}H-NMR** (400 MHz, $CDCl_3$) δ 7.85 (dd, J = 7.8, 1.4 Hz, 1H), 7.44 (ddd, J = 8.1, 7.3, 1.4 Hz, 1H), 7.31-7.28 (m, 1H), 7.17 (ddd, J = 7.8, 7.3, 1.1 Hz, 1H), 3.67 (s, 3H), 3.46 (dd, J = 17.5, 7.8 Hz, 1H), 3.15-3.13 (m, 1H), 2.97 (dd, J = 17.5, 5.7 Hz, 1H), 2.40 (s, 3H), 1.25 (d, J = 7.2 Hz, 3H); **^{13}C-NMR** (100 MHz, $CDCl_3$) δ 198.8, 176.3, 142.3, 134.0, 132.2, 130.1, 125.0, 123.4, 51.8, 43.2, 34.9, 17.2, 15.8; **IR** (film, cm^{-1}) 1731, 1670, 1433, 1210, 1168, 751; **MS** (ESI^+) *m/z* (%) 275 (100), 253 (53); **HRMS** (ESI^+) calc. for $C_{13}H_{17}O_3S$ $(M+H)^+$: 253.08929, found: 253.08859.

1-Methyl-3-[2-(methylthio)benzoyl]pyrrolidine-2,5-dione (Table 2, Entry 2)

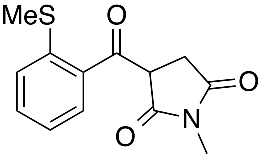

Following the general procedure the product (116 mg, 0.44 mmol) was isolated by FC (petrol/ether gradient from 1:1 to 2:8) in 98% yield as a clear oil, starting from 2-(methylthio)benzaldehyde (58 µL, 0.45 mmol) and *N*-methylmaleimide (76 µL, 0.68 mmol) in the presence of **3a** (6.6 mg, 0.0045 mmol, 1 mol%) and using acetone (225 mL) as solvent. **^{1}H-NMR** (400 MHz, $CDCl_3$) (4:1 mixture of tautomers; * denotes minor tautomer) δ 11.46* (bs, 1H), 8.12 (dd, J = 7.9, 1.4 Hz, 1H), 7.54 (ddd, J = 8.2, 7.3, 1.4 Hz, 1H), 7.40-7.38* (m, 1H), 7.37-7.33 (m, 1H), 7.31-7.27 (m, 1H + 1H*), 7.20* (dd, J = 7.3, 1.1 Hz, 1H), 4.85 (dd, J = 8.9, 4.0 Hz, 1H), 3.38 (dd, J = 18.1, 4.0 Hz, 1H), 3.19* (s, 2H), 3.05* (s, 3H), 2.97 (s, 3H), 2.84 (dd, J = 18.1, 8.9 Hz, 1H), 2.46* (s, 3H), 2.43 (s, 3H); **^{13}C-NMR** (100 MHz, $CDCl_3$) (* denotes minor tautomer) δ 192.6, 175.7, 175.0*, 174.3*, 172.8, 164.0*, 144.3, 137.2*, 133.2, 132.7*, 132.6, 131.9*, 130.7*, 128.5, 126.0*, 125.1, 124.9*, 123.6, 98.5*, 49.4, 32.1*, 31.9, 25.2, 24.3*, 15.9, 15.8*; **IR** (film, cm^{-1}) 1697, 1665, 1433, 1278, 1116, 755; **MS** (ESI^+) *m/z* (%)

549 (80), 286 (100), 264 (46); **HRMS** (ESI$^+$) calc. for $C_{13}H_{14}O_3NS$ $(M+H)^+$: 264.06833, found: 264.06889. These data are consistent with the reported values.[4]

Cyclohexyl 2-(methylthio)phenyl methanone (Table 2, Entry 3)

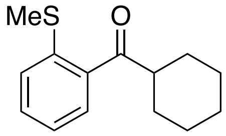

Following the general procedure the product (42 mg, 0.18 mmol) was isolated by FC (petrol/EtOAc 94:6) in 60% yield as a clear oil, starting from 2-(methylthio)benzaldehyde (39 µL, 0.30 mmol) and cyclohexene (46 µL, 0.45 mmol) in the presence of **3a** (22 mg, 0.015 mmol, 5 mol%) and using acetone (150 mL) as solvent. **^{1}H-NMR** (400 MHz, $CDCl_3$) δ 7.69 (dd, J = 7.8, 1.4 Hz, 1H), 7.43 (ddd, J = 8.1, 7.2, 1.4 Hz, 1H), 7.34-7.32 (m, 1H), 7.18 (ddd, J = 7.8, 7.2, 1.0 Hz, 1H), 3.20-3.15 (m, 1H), 2.42 (s, 3H), 1.90-1.80 (m, 4H), 1.72-1.69 (m, 1H), 1.52-1.46 (m, 2H), 1.39-1.23 (m, 3H); **^{13}C-NMR** (100 MHz, $CDCl_3$) δ 205.7, 141.3, 135.5, 131.5, 129.2, 125.8, 123.7, 47.5, 29.2, 26.0, 25.8, 16.3; **IR** (film, cm^{-1}) 2927, 2852, 1664, 1433, 1242, 1204, 972, 737; **MS** (ESI$^+$) *m/z* (%) 257 (100), 235 (19); **HRMS** (ESI$^+$) calc. for $C_{14}H_{19}OS$ $(M+H)^+$: 235.11511, found: 235.11465. These data are consistent with the reported values.[11]

Cyclopentyl 2-(methylthio)phenyl methanone (Table 2, Entry 4)

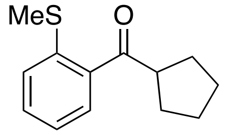

Following the general procedure the product (56 mg, 0.25 mmol) was isolated by FC (petrol/EtOAc 92:8) in 85% yield as a clear oil, starting from 2-(methylthio)benzaldehyde (39 µL, 0.30 mmol) and cyclopentene (41 µL, 0.45 mmol) in the presence of **3a** (22 mg, 0.015 mmol, 5 mol%) and using acetone (150 mL) as solvent. **^{1}H-NMR** (400 MHz, $CDCl_3$) δ 7.79 (dd, J = 7.8, 1.4 Hz, 1H), 7.43 (ddd, J = 8.2, 7.1, 1.4 Hz, 1H), 7.32-7.30 (m, 1H), 7.19-7.15 (m, 1H), 3.68-3.64 (m, 1H), 2.41 (s, 3H), 1.93-1.86 (m, 4H), 1.75-1.59 (m, 4H); **^{13}C-NMR** (100 MHz, $CDCl_3$) δ 204.4, 142.2, 135.1, 131.8, 130.0, 125.3, 123.6, 47.9, 30.1, 26.3, 16.2; **IR** (film, cm^{-1}) 2952, 2867, 1665, 1433, 1212, 738; **MS** (ESI$^+$) *m/z* (%) 463 (92), 243 (100), 221 (43); **HRMS** (ESI$^+$) calc. for $C_{13}H_{17}OS$ $(M+H)^+$: 221.09946, found: 221.09898.

2-(Methylthio)phenyl tetrahydro-2*H*-pyran-2-yl methanone (Table 2, Entry 5)

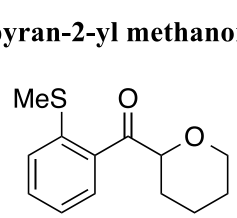

Following the general procedure the product (67 mg, 0.28 mmol) was isolated by FC (petrol/ether 8:2) in 94% yield as a clear oil, starting from 2-(methylthio)benzaldehyde (39 µL, 0.30 mmol) and 3,4-dihydro-2*H*-pyran (42 µL, 0.45 mmol) in the presence of **3a** (13 mg, 0.009 mmol, 3 mol%) and using acetone (150 mL) as solvent. **^{1}H-NMR** (400 MHz, $CDCl_3$) δ 7.71 (dd, J = 7.8, 1.4 Hz, 1H), 7.42 (ddd, J = 8.1, 7.3, 1.4 Hz, 1H), 7.33-7.31 (m, 1H), 7.18-7.14 (m, 1H), 4.61 (dd, J = 10.3, 2.5 Hz, 1H), 4.09-

4.05 (m, 1H), 3.60-3.54 (m, 1H), 2.42 (s, 3H), 1.93-1.84 (m, 2H), 1.74-1.52 (m, 4H); **^{13}C-NMR** (100 MHz, $CDCl_3$) δ 200.1, 141.5, 134.2, 131.7, 129.8, 126.0, 123.6, 80.3, 68.5, 28.0, 25.4, 22.9, 16.3; **IR** (film, cm^{-1}) 2938, 2849, 1677, 1433, 1089, 1045, 972, 905, 735; **MS** (ESI^+) *m/z* (%) 511 (31), 275 (18), 259 (100), 237 (19); **HRMS** (ESI^+) calc. for $C_{13}H_{17}O_2S$ $(M+H)^+$: 237.09384, found: 237.09438.

2-(Methylthio)phenyl tetrahydrofuran-2-yl methanone (Table 2, Entry 6)

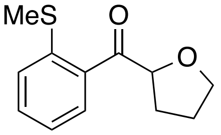

Following the general procedure the product (63 mg, 0.28 mmol) was isolated by FC (petrol/ether 7:3) in 94% yield as a clear oil, starting from 2-(methylthio)benzaldehyde (39 µL, 0.30 mmol) and 2,3-dihydrofuran (35 µL, 0.45 mmol) in the presence of **3a** (4.4 mg, 0.003 mmol, 1 mol%) and using acetone (150 mL) as solvent. **^{1}H-NMR** (400 MHz, $CDCl_3$) δ 7.80 (dd, J = 7.8, 1.4 Hz, 1H), 7.44 (ddd, J = 8.2, 7.2, 1.4 Hz, 1H), 7.33-7.30 (m, 1H), 7.17 (ddd, J = 7.8, 7.2, 1.0 Hz, 1H), 5.18 (t, J = 6.9 Hz, 1H), 4.01-3.89 (m, 2H), 2.41 (s, 3H), 2.20-2.14 (m, 2H), 1.97-1.89 (m, 2H); **^{13}C-NMR** (100 MHz, $CDCl_3$) δ 200.5, 142.5, 133.5, 132.2, 130.3, 125.6, 123.6, 80.6, 69.5, 28.9, 25.6, 16.2; **IR** (film, cm^{-1}) 2977, 2872, 1676, 1433, 1219, 1077, 738; **MS** (ESI^+) *m/z* (%) 467 (100), 223 (16); **HRMS** (ESI^+) calc. for $C_{12}H_{15}O_2S$ $(M+H)^+$: 223.07873, found: 223.07829.

[(2*R*,4*R*,5*S*,6*R*)-4,5-dihydroxy-6-(hydroxymethyl)tetrahydro-2*H*-pyran-2-yl]-2-(methylthio)phenyl methanone (Table 2, Entry 7)

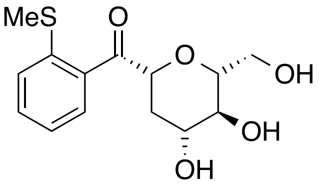

Following the general procedure the product (76 mg, 0.26 mmol) was isolated by FC (gradient from pure DCM to DCM/MeOH 85:15) in 85% yield as an off-white solid, starting from 2-(methylthio)benzaldehyde (39 µL, 0.30 mmol) and D-Glucal (68 mg, 0.45 mmol) in the presence of **3a** (22 mg, 0.015 mmol, 5 mol%) and using acetone (150 mL) as solvent. **^{1}H-NMR** (400 MHz, MeOD) (4:1 mixture of diastereoisomers; * denotes minor isomer) δ 7.93* (dd, J = 7.8, 1.4 Hz, 1H), 7.88 (dd, J = 7.9, 1.4 Hz, 1H), 7.62-7.59* (m, 2H), 7.56-7.44 (m, 2H), 7.27-7.19 (m, 1H), 5.25* (dd, J = 6.2, 1.5 Hz, 1H), 5.01 (dd, J = 12.0, 2.1 Hz, 1H), 3.88 (dd, J = 12.2, 2.3 Hz, 1H), 3.76-3.70 (m, 2H), 3.60-3.58* (m, 2H), 3.44-3.38 (m, 1H), 3.32-3.26 (m, 1H), 3.22-3.17* (m, 1H), 2.49* (ddd, J = 13.2, 4.9, 1.6 Hz, 1H), 2.43 (s, 3H), 2.42* (s, 3H), 2.14 (ddd, J = 12.9, 5.0, 2.1 Hz, 3H), 1.78* (ddd, J = 13.2, 11.4, 6.3 Hz, 1H), 1.53 (dt, J = 12.9, 11.7 Hz, 1H); **^{13}C-NMR** (100 MHz, MeOD) (* denotes minor isomer) δ 204.0*, 200.2, 143.9, 135.8*, 134.1, 133.8, 133.3*, 131.66*, 131.52, 127.0, 124.9, 124.7*, 82.0, 78.7, 78.4*, 77.3*, 73.5, 72.9*, 72.6, 70.6*, 62.8, 62.6*, 37.2, 34.0*, 16.1; **IR** (film, cm^{-1}) 3384, 3355, 1661, 1434, 1226, 1066, 736; **MS** (ESI^+) *m/z* (%) 619 (100), 321 (46); **HRMS** (ESI^+) calc. for $C_{14}H_{18}O_5NaS$ $(M+Na)^+$: 321.07672, found: 321.07578; **M.p.** (MeOH/DCM): 156-158 ºC.

2-ethoxy-1-[2-(methylthio)phenyl]butan-1-one (Table 3, Entry 8)

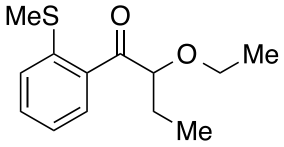

Following the general procedure the product (65 mg, 0.27 mmol) was isolated by FC (petrol/DCM 1:1) in 91% yield as a clear oil, starting from 2-(methylthio)benzaldehyde (39 µL, 0.30 mmol) and ethyl propenyl ether (89 µL, 0.80 mmol) in the presence of **3a** (22 mg, 0.015 mmol, 5 mol%) and using acetone (38 mL) as solvent. **^{1}H-NMR** (400 MHz, $CDCl_3$) δ 7.96 (dd, J = 7.8, 1.4 Hz, 1H), 7.46 (ddd, J = 8.1, 7.3, 1.4 Hz, 1H), 7.35-7.33 (m, 1H), 7.18 (ddd, J = 7.8, 7.3, 1.1 Hz, 1H), 4.41 (dd, J = 7.6, 5.3 Hz, 1H), 3.58 (dq, J = 9.1, 7.0 Hz, 1H), 3.45 (dq, J = 9.1, 7.0 Hz, 1H), 2.43 (s, 3H), 1.87-1.75 (m, 2H), 1.19 (t, J = 7.0 Hz, 3H), 0.99 (t, J = 7.4 Hz, 3H); **^{13}C-NMR** (100 MHz, $CDCl_3$) δ 202.6, 142.3, 134.0, 132.1, 130.1, 125.8, 123.6, 85.4, 65.7, 26.4, 16.3, 15.4, 10.3; **IR** (film, cm^{-1}) 2974, 1677, 1434, 1216, 1123, 1078, 739; **MS** (ESI$^+$) *m/z* (%) 499 (100), 239 (37); **HRMS** (ESI$^+$) calc. for $C_{13}H_{19}O_2S$ $(M+H)^+$: 239.11003, found: 239.10957.

Methyl 2-[2-(methylthio)benzoyl]butanoate (Table 2, Entry 9)

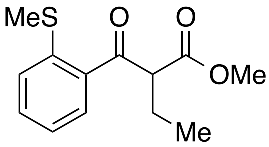

Following the general procedure the product (68 mg, 0.27 mmol) was isolated by FC (petrol/ether 8:2) in 90% yield as a clear oil, starting from 2-(methylthio)benzaldehyde (39 µL, 0.30 mmol) and methyl crotonate (48 µL, 0.45 mmol) in the presence of **3a** (22 mg, 0.015 mmol, 5 mol%) and using acetone (150 mL) as solvent. **^{1}H-NMR** (400 MHz, $CDCl_3$) (5:1 mixture of tautomers; * denotes minor tautomer) δ 12.63* (s, 1H), 7.85 (dd, J = 7.9, 1.5 Hz, 1H), 7.47 (ddd, J = 8.1, 7.3, 1.4 Hz, 1H), 7.38-7.33 (m, 1H), 7.30-7.28* (m, 1H), 7.22-7.17 (m, 1H), 4.23 (t, J = 7.2 Hz, 1H), 3.85* (s, 3H), 3.67 (s, 3H), 2.45* (s, 3H), 2.43 (s, 3H), 2.03 (m, 2H), 0.97 (t, J = 7.3 Hz, 3H), 0.89* (t, J = 7.3 Hz, 3H); **^{13}C-NMR** (100 MHz, $CDCl_3$) (* denotes minor tautomer) δ 196.0, 173.7*, 170.4, 169.3*, 143.3, 137.0*, 134.2*, 133.9, 132.5, 130.4, 129.7*, 128.5*, 126.1*, 125.4, 124.8*, 123.6, 104.7*, 56.8, 52.4, 51.7*, 22.7, 20.2*, 16.1, 14.6*, 12.1; **IR** (film, cm^{-1}) 2969, 1737, 1670, 1433, 1208, 980, 744; **MS** (ESI$^+$) *m/z* (%) 527 (100), 253 (17); **HRMS** (ESI$^+$) calc. for $C_{13}H_{17}O_3S$ $(M+H)^+$: 253.08929, found: 253.08859.

Ethyl 2-[2-(methylthio)benzoyl]butanoate (Table 2, Entry 10)

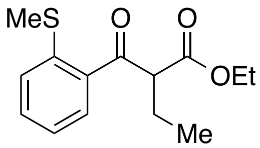

Following the general procedure the product (67 mg, 0.25 mmol) was isolated by FC (petrol/ether 8:2) in 84% yield as a clear oil, starting from 2-(methylthio)benzaldehyde (39 µL, 0.30 mmol) and ethyl crotonate (58 µL, 0.45 mmol) in the presence of **3a** (22 mg, 0.015 mmol, 5 mol%) and using acetone (150 mL) as solvent. **^{1}H-NMR** (400 MHz, $CDCl_3$) (15:1 mixture of tautomers; * denotes minor tautomer) δ 12.71* (s, 1H), 7.85 (dd, J = 7.8, 1.5 Hz, 1H), 7.46 (ddd, J = 8.1, 7.3, 1.5 Hz, 1H), 7.34-

7.32 (m, 1H), 7.19 (ddd, *J* = 7.8, 7.3, 1.1 Hz, 1H), 4.30* (q, *J* = 7.1 Hz, 1H), 4.20-4.09 (m, 3H), 2.45* (s, 3H), 2.42 (s, 3H), 2.03 (app. quintet, *J* = 7.3 Hz, 2H), 1.34* (t, *J* = 7.1 Hz, 3H), 1.16 (t, *J* = 7.1 Hz, 3H), 0.97 (t, *J* = 7.4 Hz, 3H), 0.90* (t, *J* = 7.4 Hz, 3H); **^{13}C-NMR** (100 MHz, $CDCl_3$) (* denotes minor tautomer) δ 196.2, 170.0, 143.1, 134.3, 132.4, 130.4, 129.7*, 128.6*, 126.3*, 125.5, 124.9*, 123.7, 61.3, 60.7*, 57.2, 22.6, 20.3*, 16.2, 14.4*, 14.1, 12.2; **IR** (film, cm^{-1}) 2975, 1733, 1672, 1433, 1253, 1179, 744; **MS** (ESI^+) *m/z* (%) 555 (100), 289 (20), 267 (13); **HRMS** (ESI^+) calc. for $C_{14}H_{19}O_3S$ $(M+H)^+$: 267.10494, found: 267.10415.

Methyl 2-[2-(methylthio)benzoyl]hexanoate (Table 2, Entry 11)

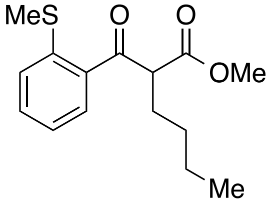

Following the general procedure the product (56 mg, 0.20 mmol) was isolated by FC (petrol/ether 85:15) in 67% yield as a clear oil, starting from 2-(methylthio)benzaldehyde (39 μL, 0.30 mmol) and methyl 2-hexenoate (64 μL, 0.45 mmol) in the presence of **3a** (22 mg, 0.015 mmol, 5 mol%) and using acetone (150 mL) as solvent. **^{1}H-NMR** (400 MHz, $CDCl_3$) (8:1 mixture of tautomers; * denotes minor tautomer) δ 12.67* (s, 1H), 7.85 (dd, *J* = 7.9, 1.4 Hz, 1H), 7.48 (ddd, *J* = 8.3, 7.1, 1.4 Hz, 1H), 7.34 (d, *J* = 8.3 Hz, 1H), 7.22-7.18 (m, 1H), 4.29 (t, *J* = 7.2 Hz, 1H), 3.83* (s, 3H), 3.67 (s, 3H), 2.46* (s, 3H), 2.43 (s, 3H), 2.02-1.97 (m, 2H), 1.36-1.29 (m, 4H), 1.16-1.05* (m, 4H), 0.87 (t, *J* = 7.3 Hz, 3H), 0.71* (t, *J* = 7.3 Hz, 3H); **^{13}C-NMR** (100 MHz, $CDCl_3$) (* denotes minor tautomer) δ 196.1, 174.1*, 170.6, 143.5, 137.2*, 133.96, 133.90*, 132.6, 130.5, 129.7*, 128.7*, 126.3*, 125.6, 124.9*, 123.7, 55.4, 52.5, 51.9*, 32.2*, 29.9, 29.1, 26.5*, 22.7, 22.5*, 16.2, 14.0, 13.8*; **IR** (film, cm^{-1}) 2955, 1738, 1672, 1433, 1227, 1194, 747; **MS** (ESI^+) *m/z* (%) 303 (100), 282 (79); **HRMS** (ESI^+) calc. for $C_{15}H_{21}O_3S$ $(M+H)^+$: 281.12059, found: 281.11972.

2-(4-methylthio-5,6-dihydro-2*H*-pyran-3-carbonyl)succinate (Table 2, Entry 12)

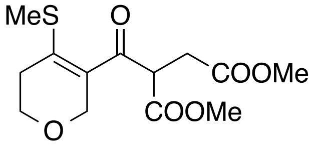

Following the general procedure the product (65 mg, 0.21 mmol) was isolated by FC (petrol/EtOAc 6:4) in 72% yield as a clear oil, starting from 4-(methylthio)-5,6-dihydro-2*H*-pyran-3-carbaldehyde (48 mg, 0.30 mmol) and dimethyl maleate (58 μL, 0.45 mmol) in the presence of **3a** (22 mg, 0.015 mmol, 5 mol%), using dichloroethane (150 mL) as solvent and heating at 80 ºC. **^{1}H-NMR** (400 MHz, $CDCl_3$) δ 4.72 (dt, *J* = 14.4, 2.1 Hz, 1H), 4.62 (dt, *J* = 14.4, 2.2 Hz, 1H), 4.08 (dd, *J* = 8.4, 6.0 Hz, 1H), 3.92-3.81 (m, 2H), 3.67 (s, 3H), 3.07 (dd, *J* = 17.6, 8.4 Hz, 1H), 2.93 (dd, *J* = 17.6, 6.0 Hz, 1H), 2.63-2.60 (m, 2H); **^{13}C-NMR** (100 MHz, $CDCl_3$) δ 190.4, 171.9, 168.8, 154.3, 125.9, 67.0, 63.9, 52.8, 52.1, 49.3, 32.9, 29.6, 14.2; **IR** (film, cm^{-1}) 2954, 2851, 1731, 1680, 1436, 1259, 1217, 1163, 1004; **MS** (ESI^+) *m/z* (%) 325 (100), 303 (73); **HRMS** (ESI^+) calc. for $C_{13}H_{19}O_6S$ $(M+H)^+$: 303.08969, found: 303.08899.

NMR spectra for organic compounds:

^{1}H NMR, 400 MHz, $CDCl_3$

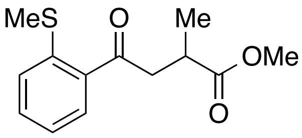

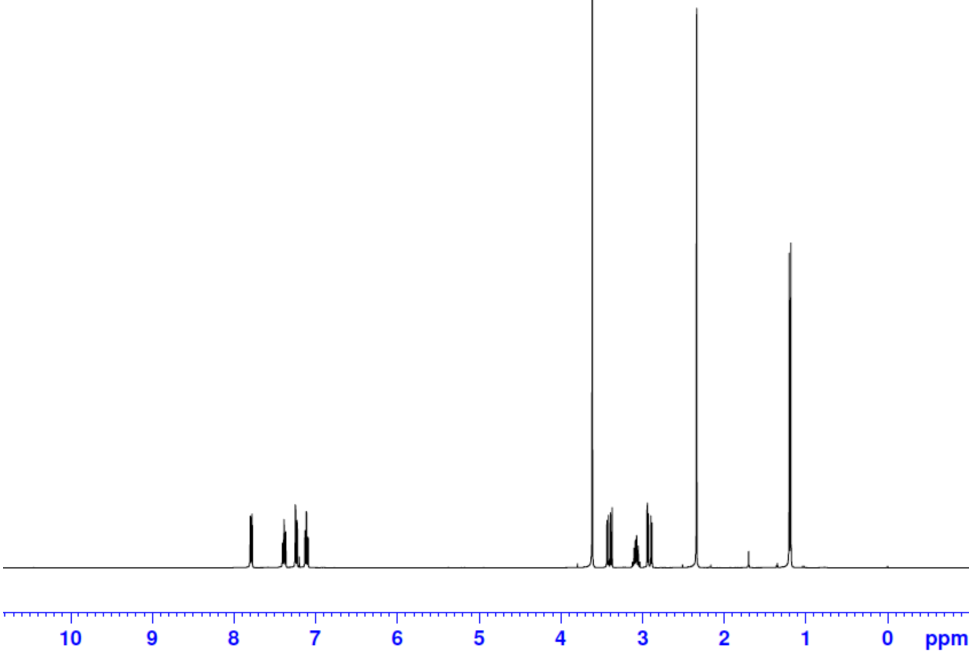

^{13}C NMR, 100 MHz, $CDCl_3$

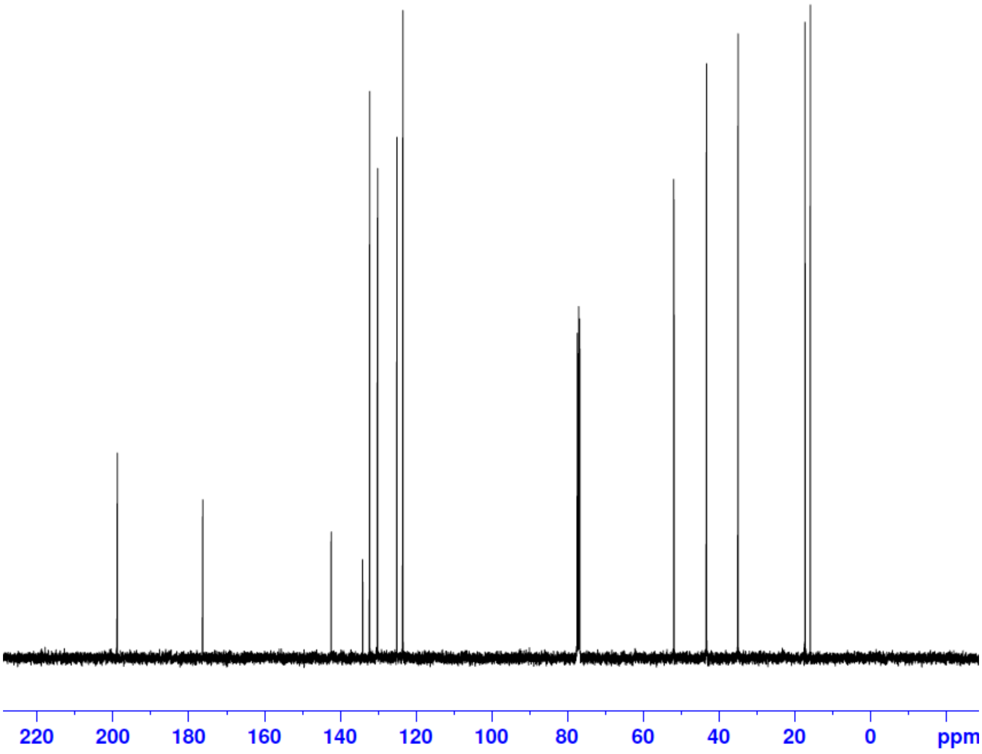

^{1}H NMR, 400 MHz, $CDCl_3$

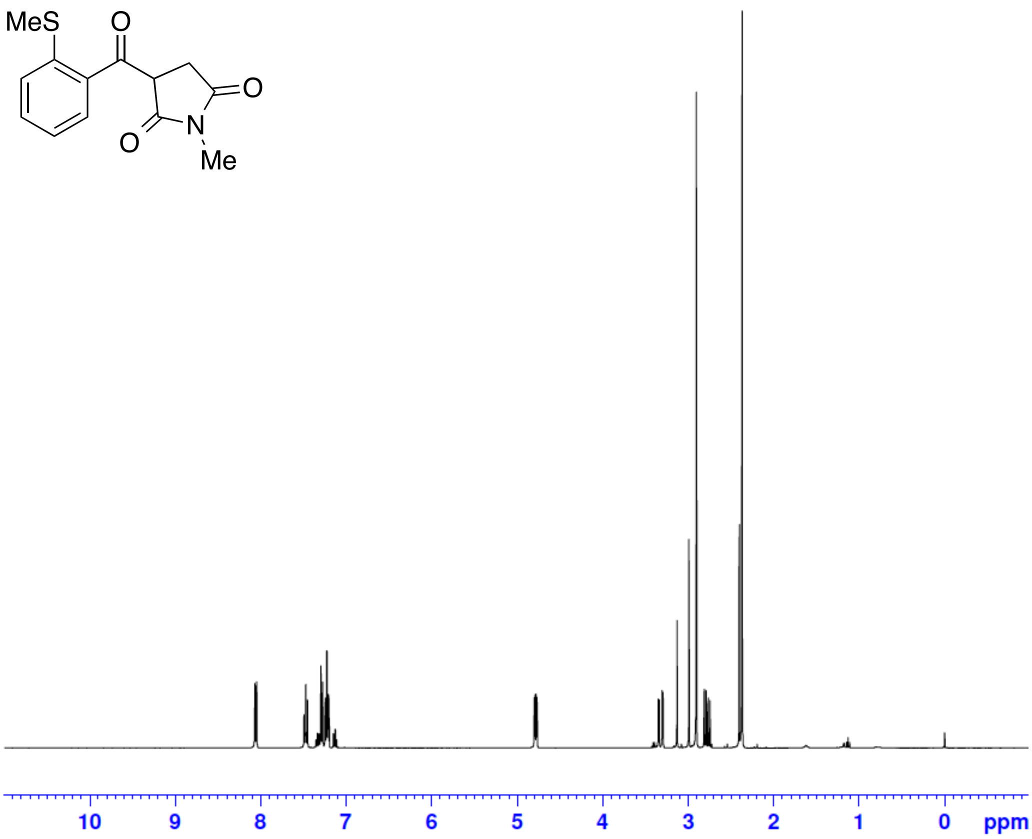

^{13}C NMR, 100 MHz, $CDCl_3$

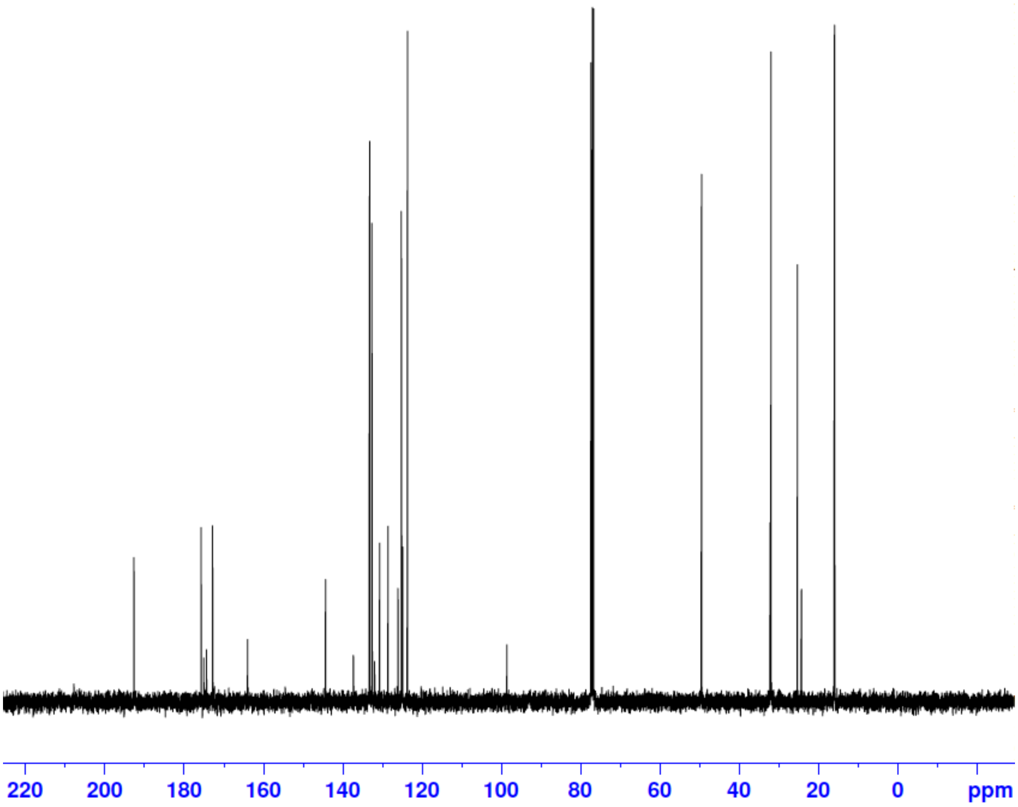

^{1}H NMR, 400 MHz, $CDCl_3$

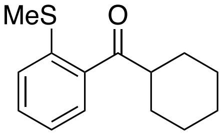

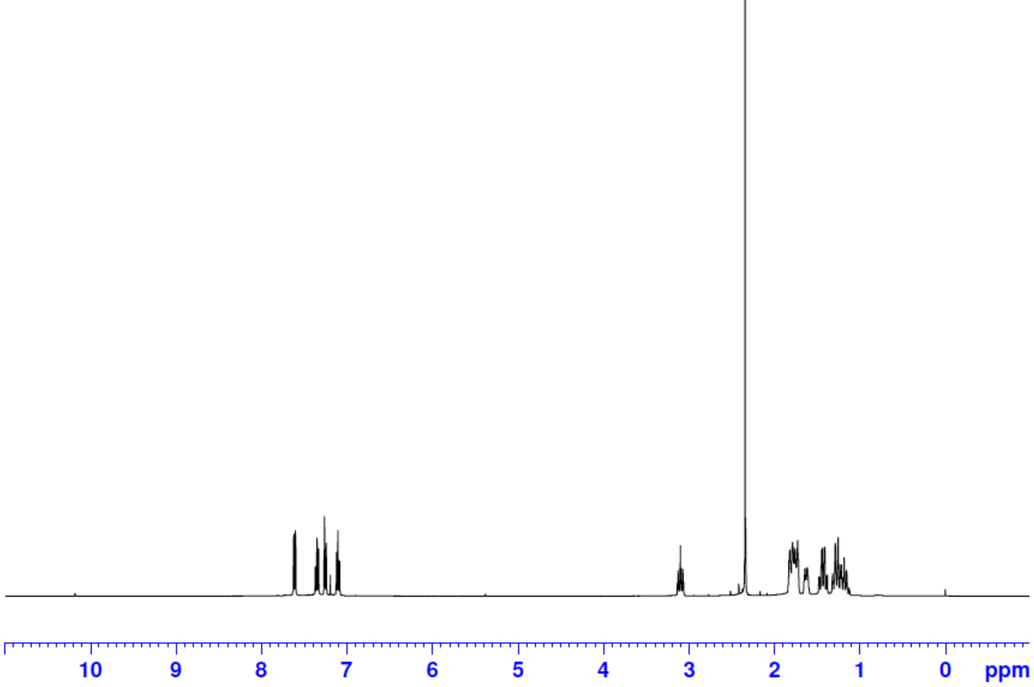

^{13}C NMR, 100 MHz, $CDCl_3$

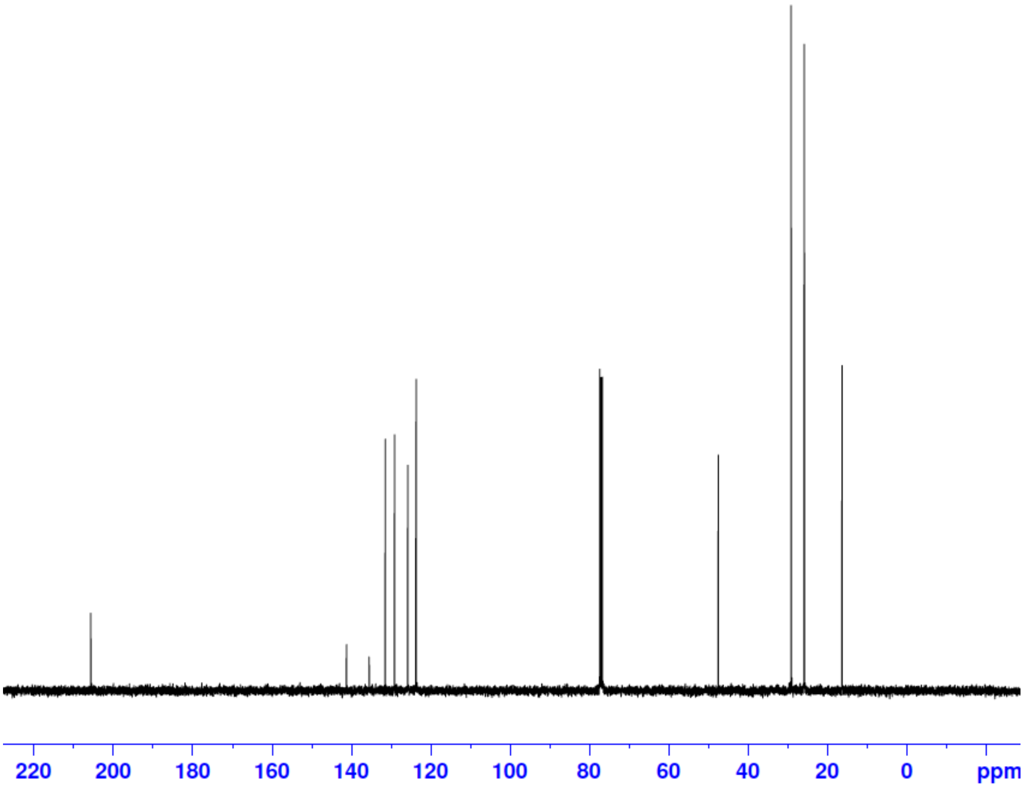

^{1}H NMR, 400 MHz, $CDCl_3$

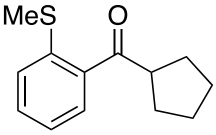

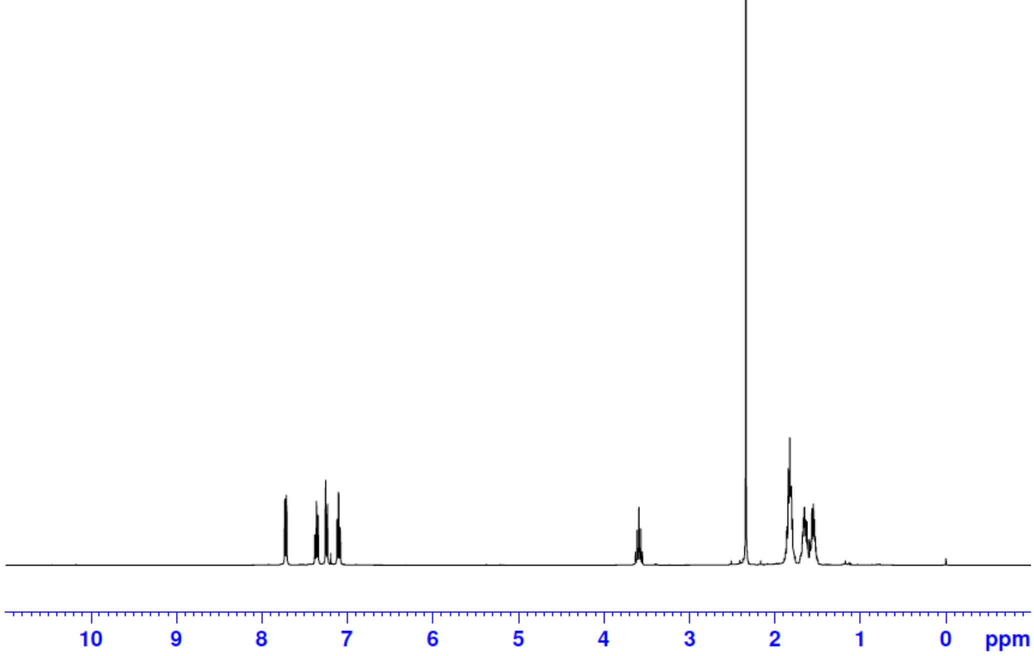

^{13}C NMR, 100 MHz, $CDCl_3$

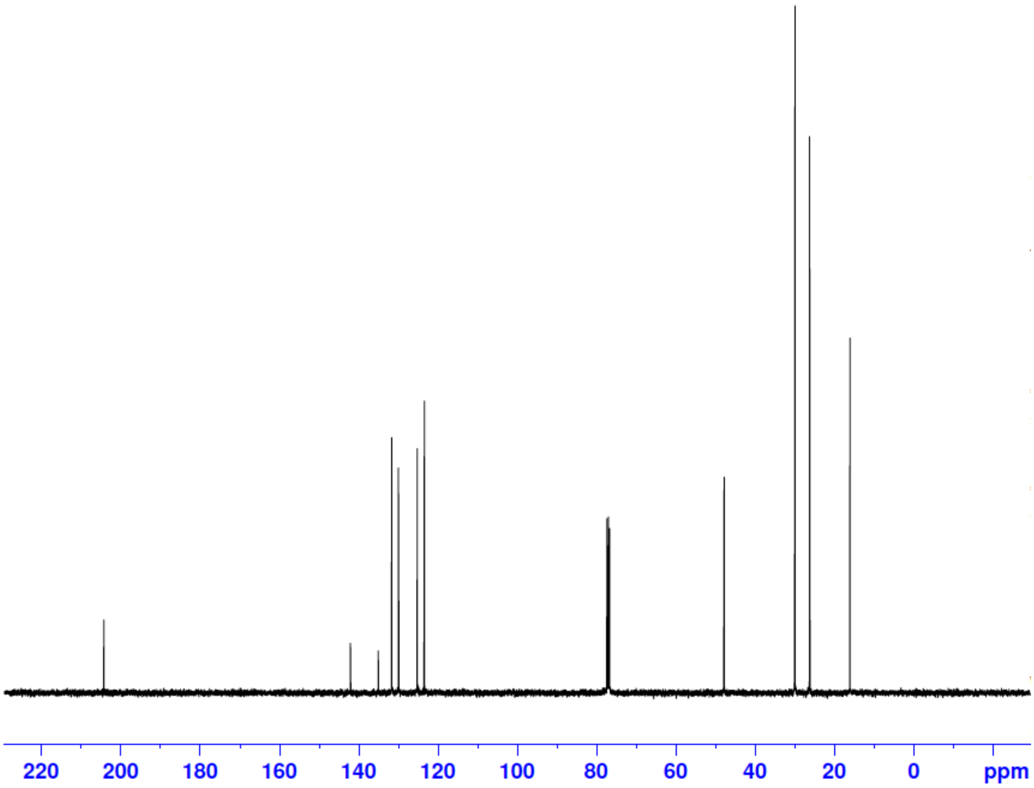

^{1}H NMR, 400 MHz, $CDCl_3$

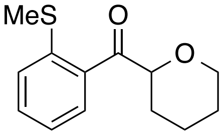

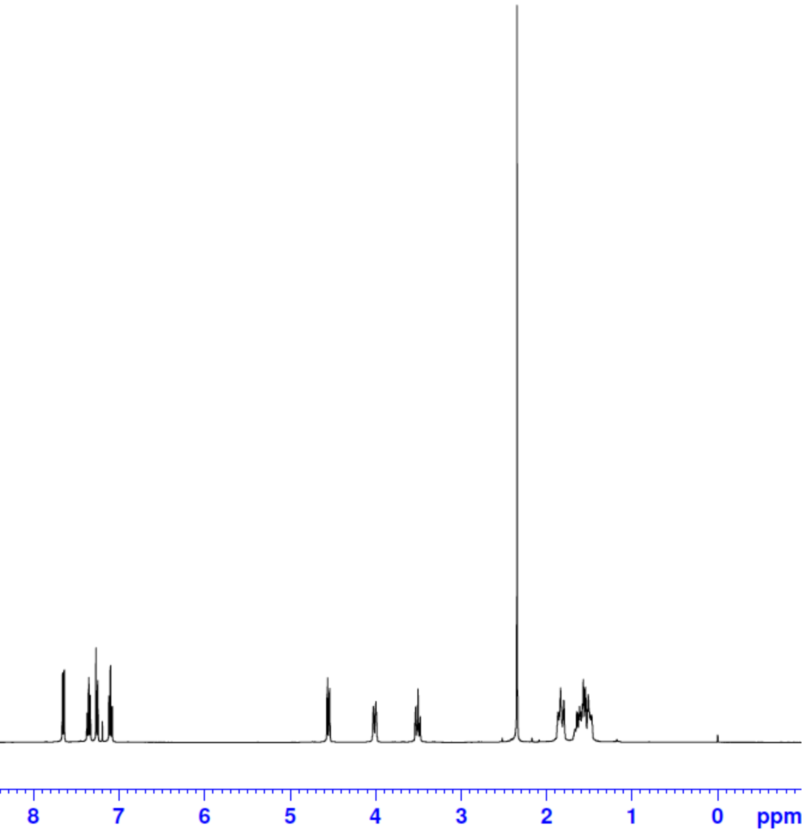

^{13}C NMR, 100 MHz, $CDCl_3$

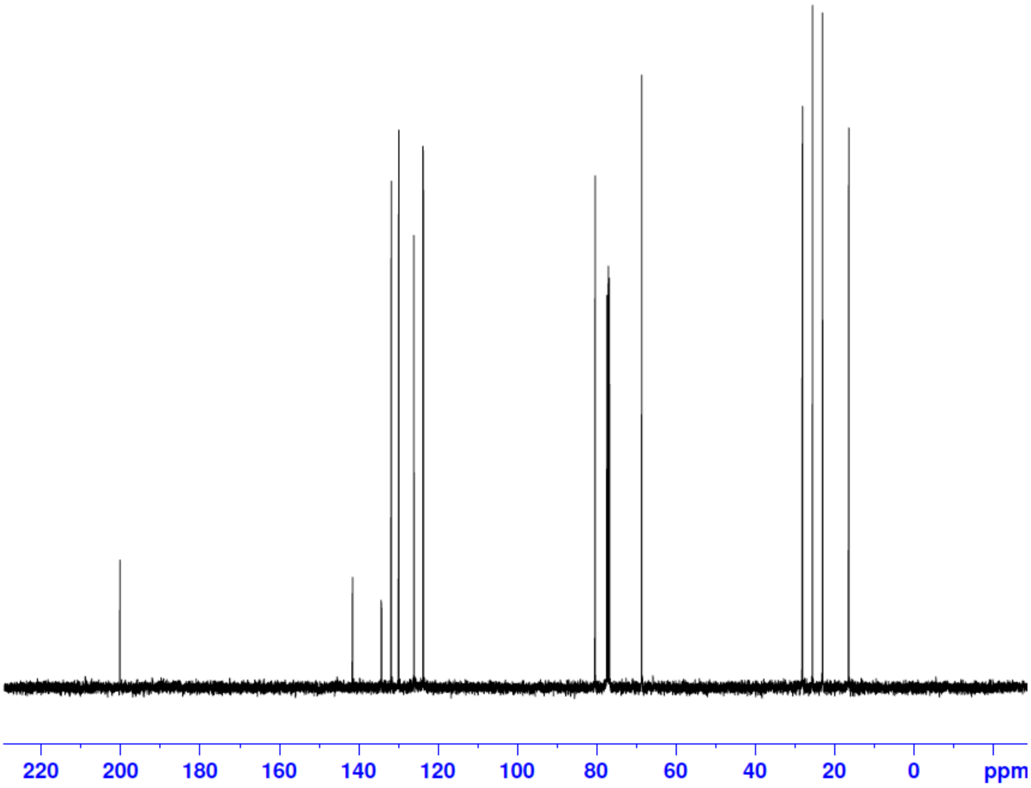

^{1}H NMR, 400 MHz, $CDCl_3$

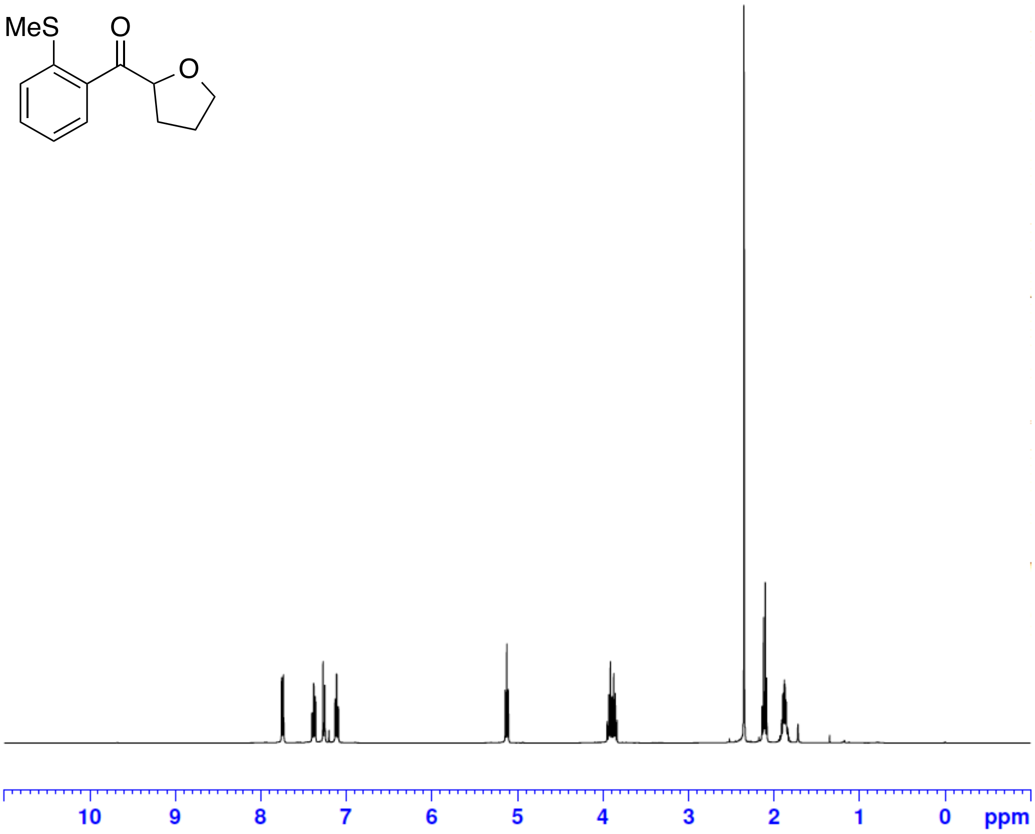

^{13}C NMR, 100 MHz, $CDCl_3$

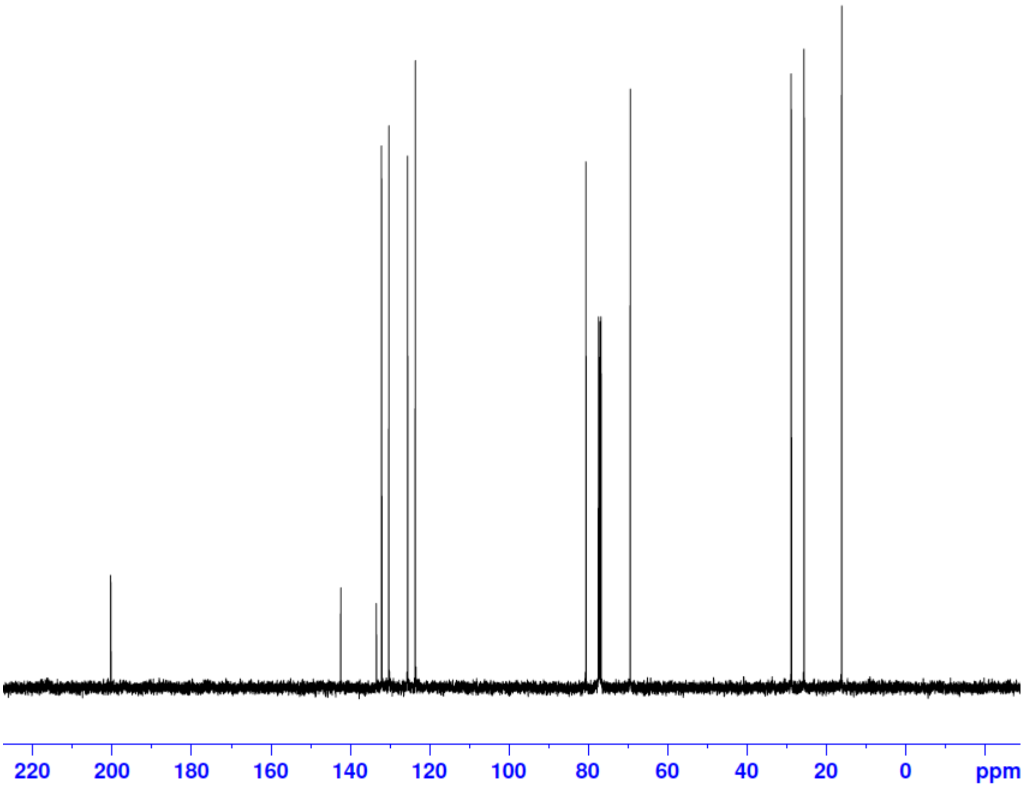

^{1}H NMR, 400 MHz, MeOD

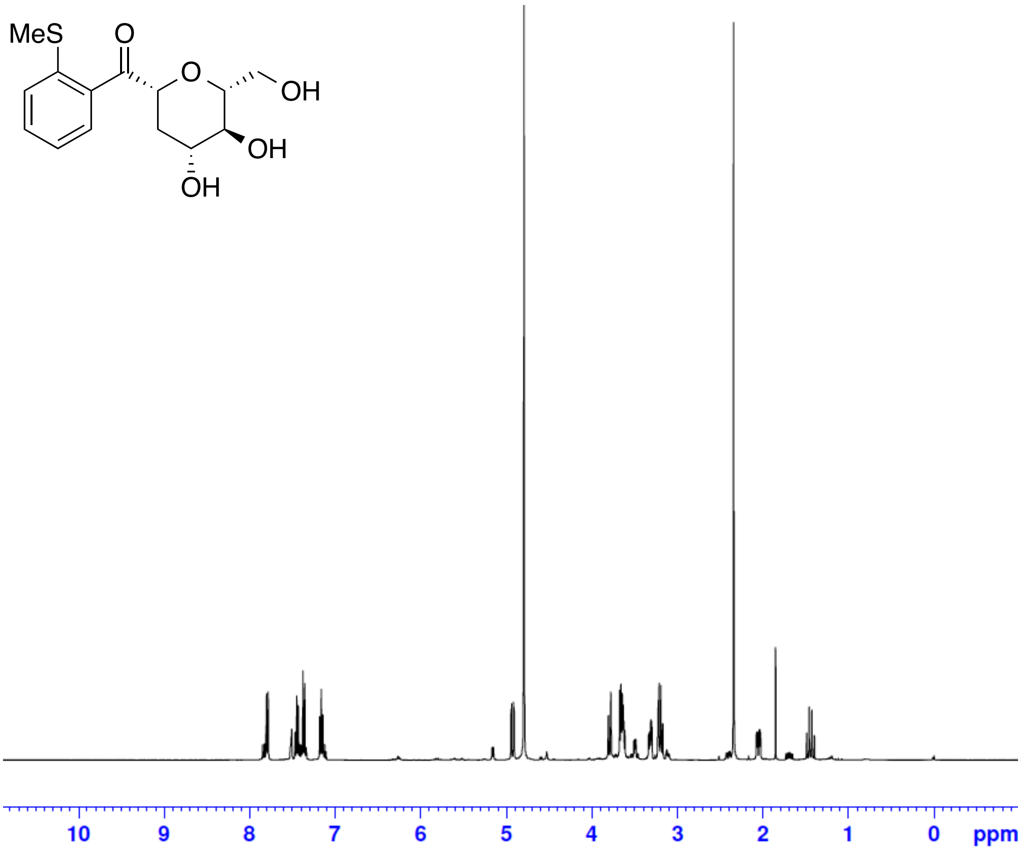

^{13}C NMR, 100 MHz, MeOD

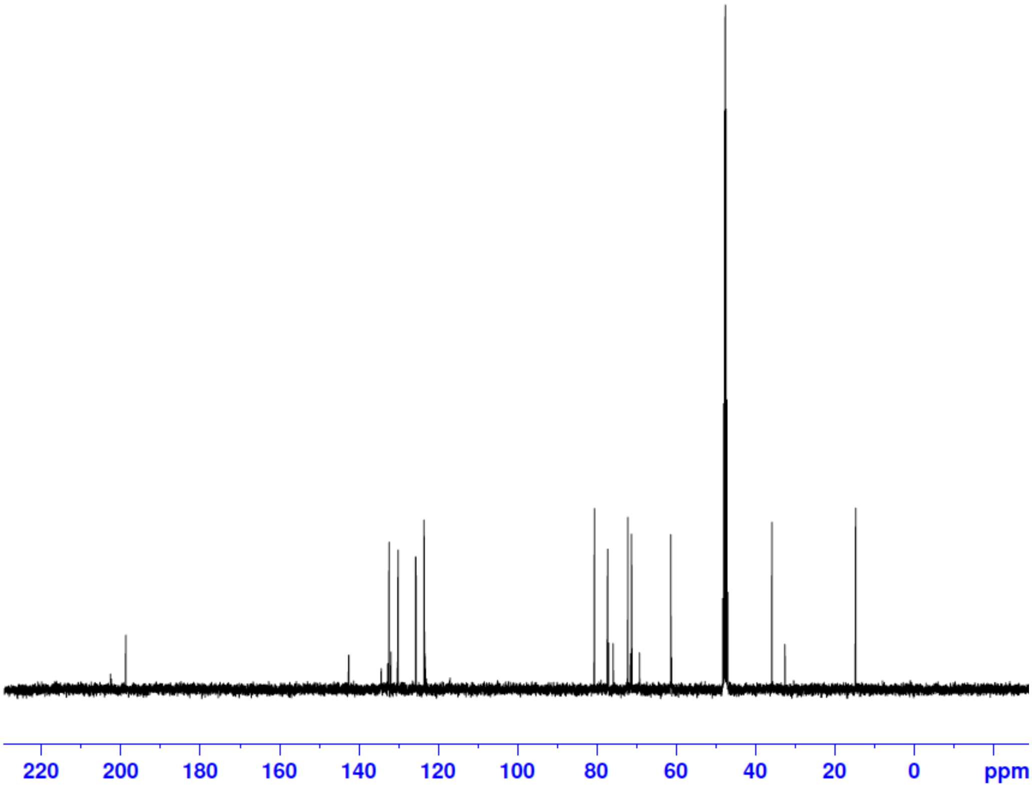

^{1}H NMR, 400 MHz, $CDCl_3$

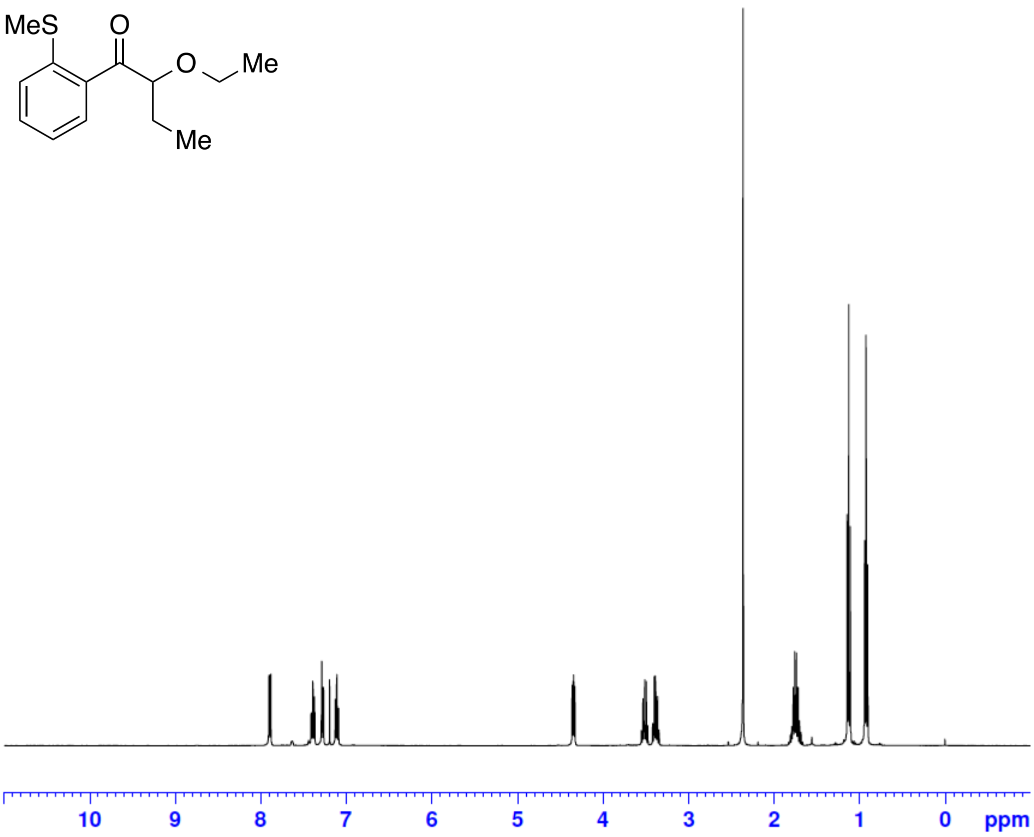

^{13}C NMR, 100 MHz, $CDCl_3$

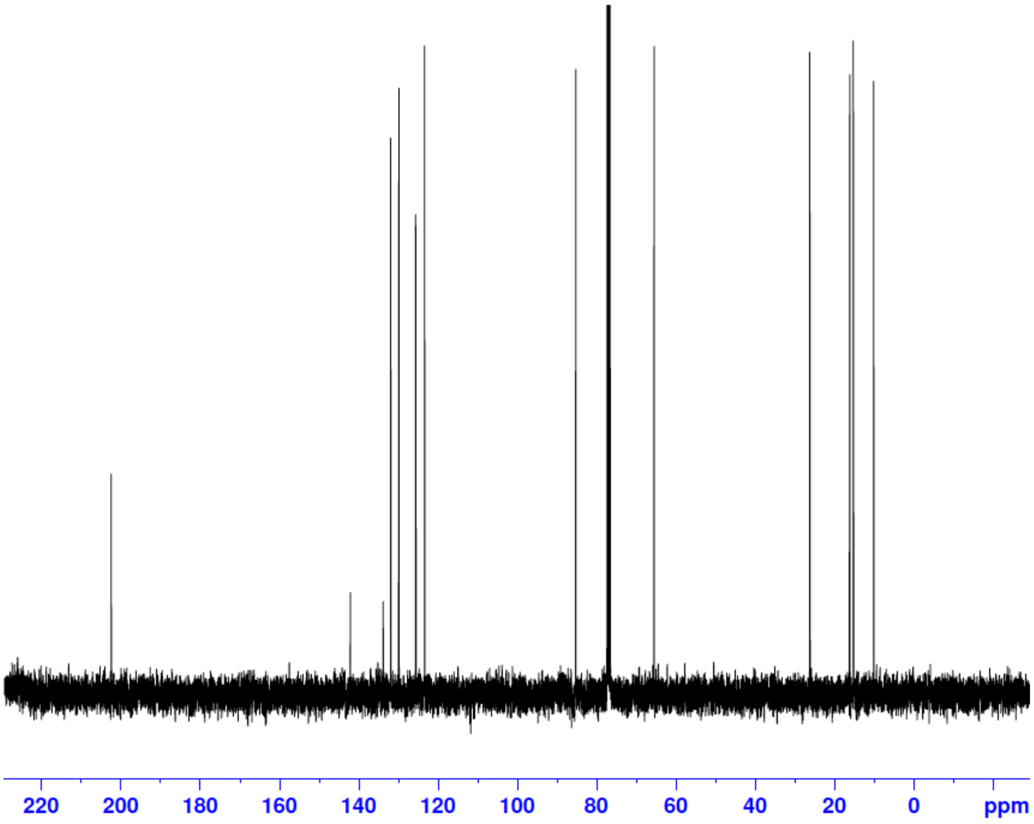

^{1}H NMR, 400 MHz, $CDCl_3$

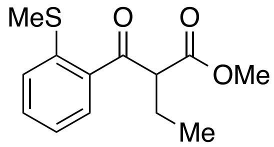

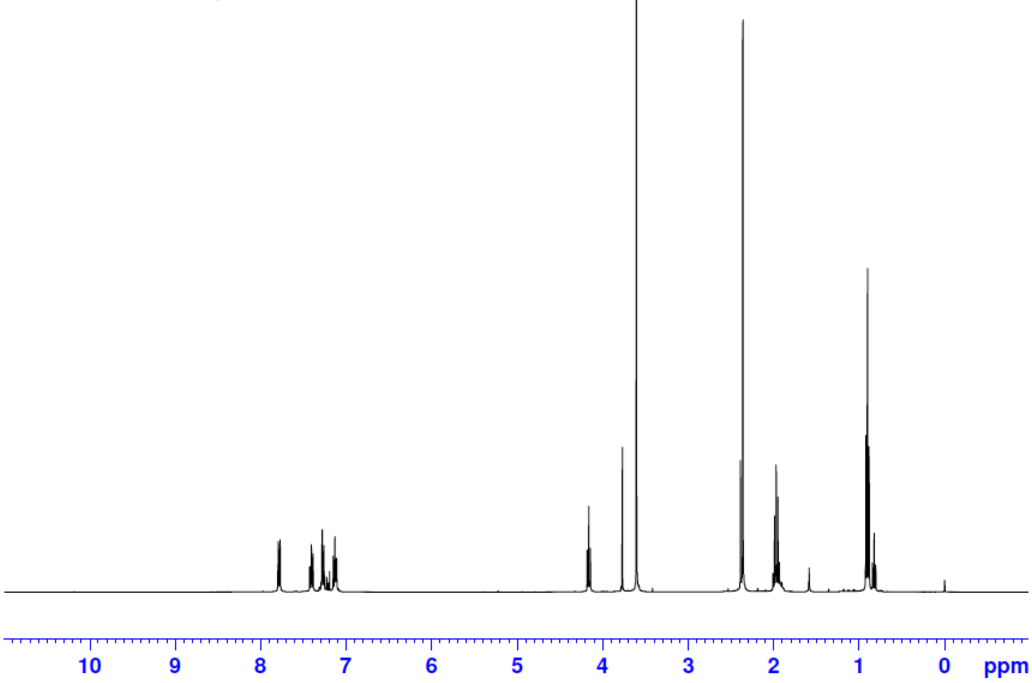

^{13}C NMR, 100 MHz, $CDCl_3$

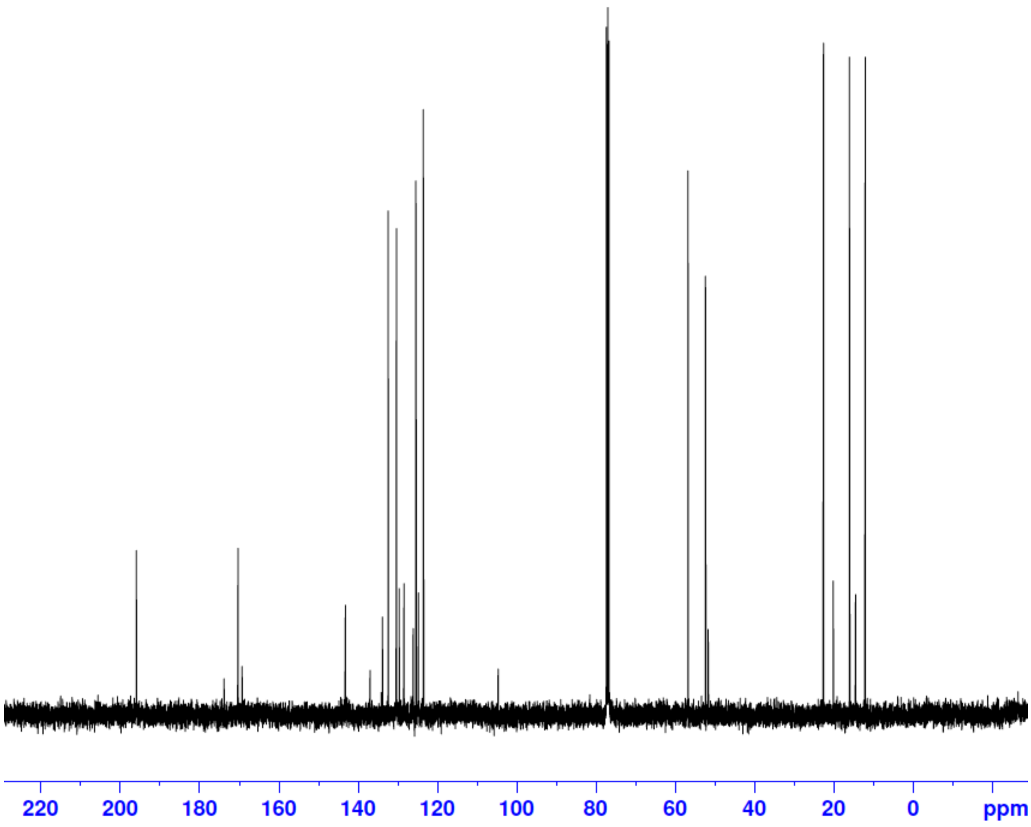

^{1}H NMR, 400 MHz, $CDCl_3$

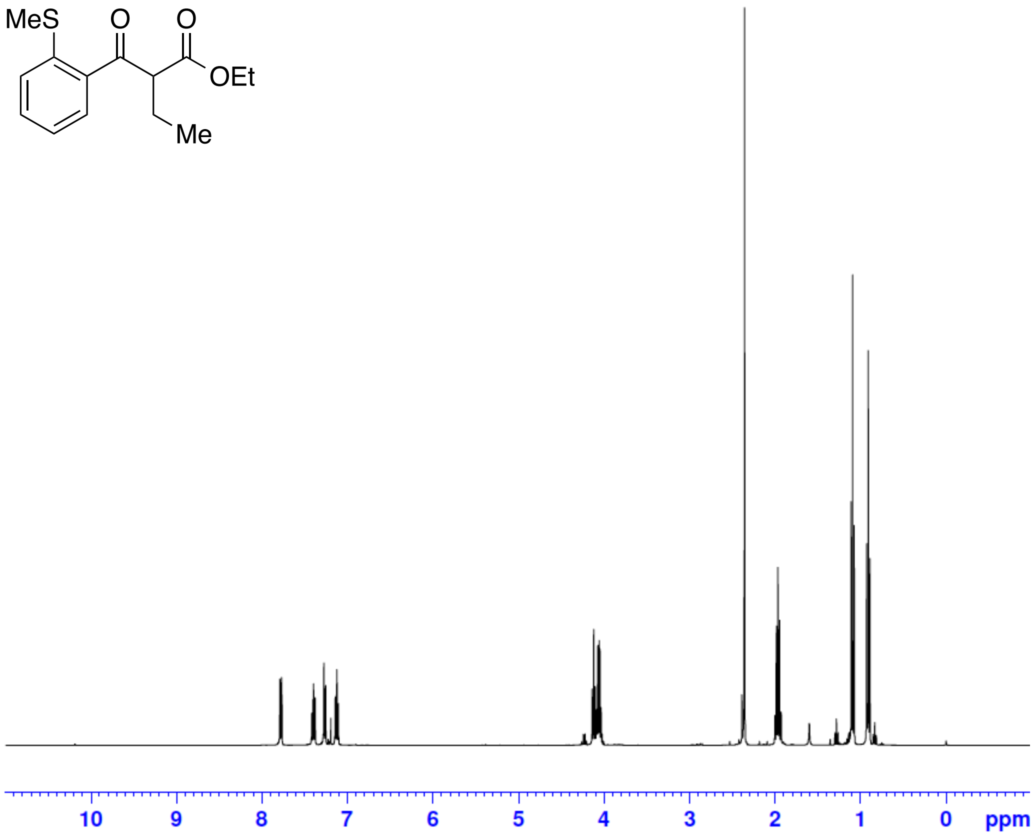

^{13}C NMR, 100 MHz, $CDCl_3$

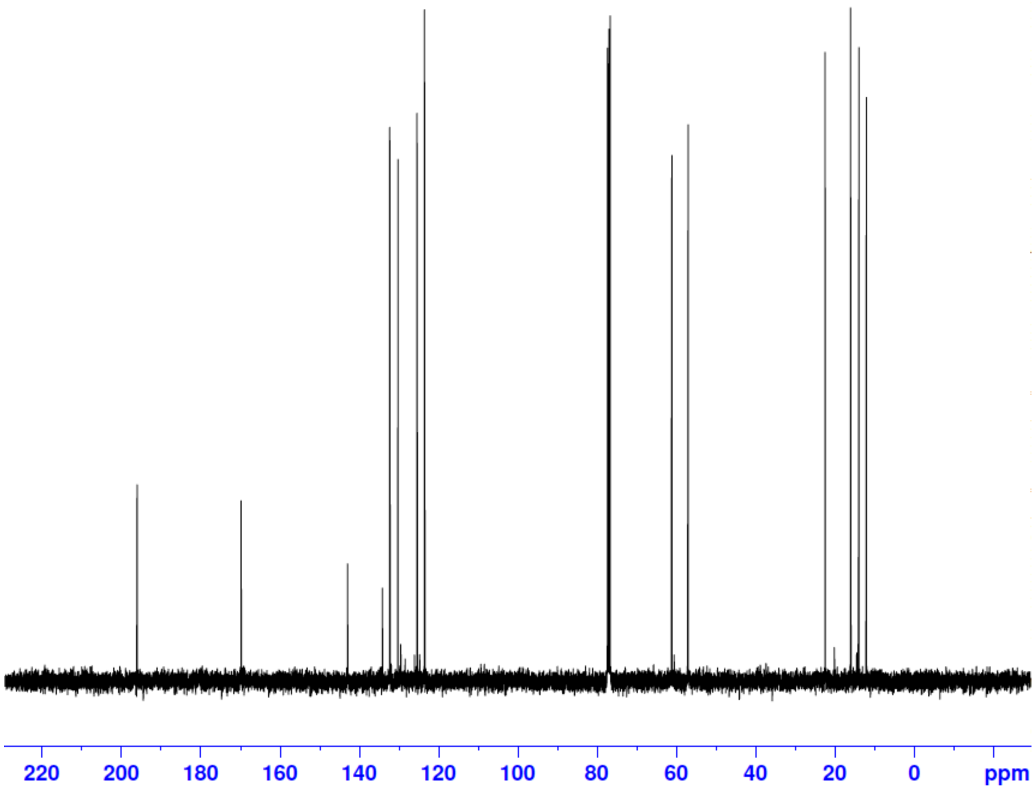

^{1}H NMR, 400 MHz, $CDCl_3$

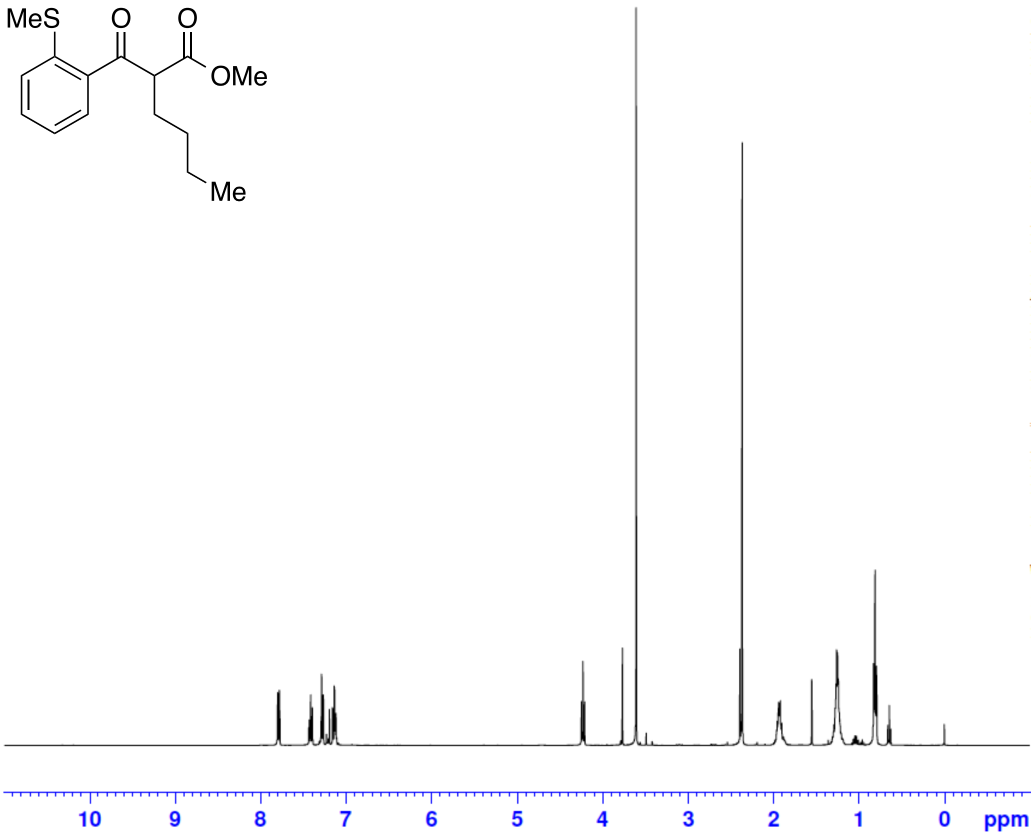

^{13}C NMR, 100 MHz, $CDCl_3$

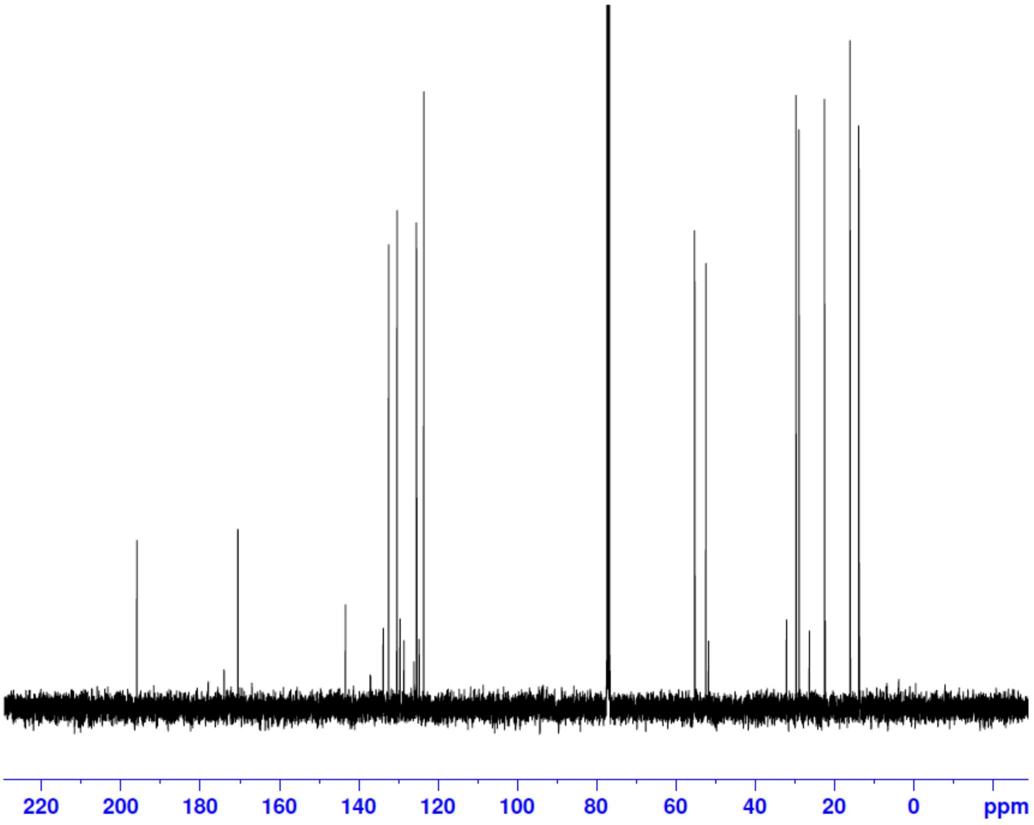

^{1}H NMR, 400 MHz, $CDCl_3$

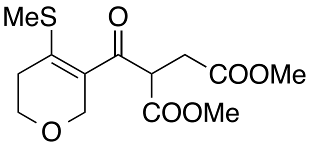

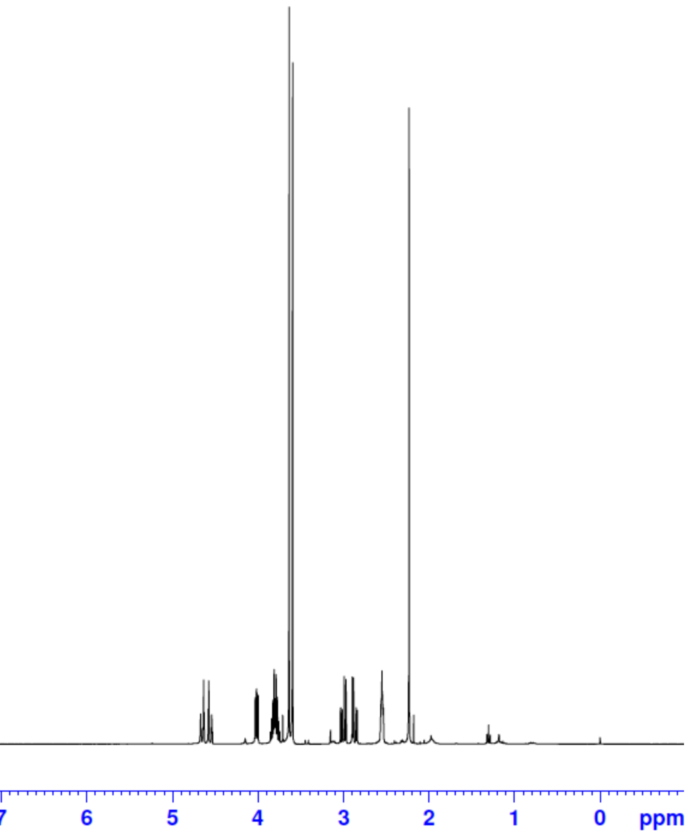

^{13}C NMR, 100 MHz, $CDCl_3$

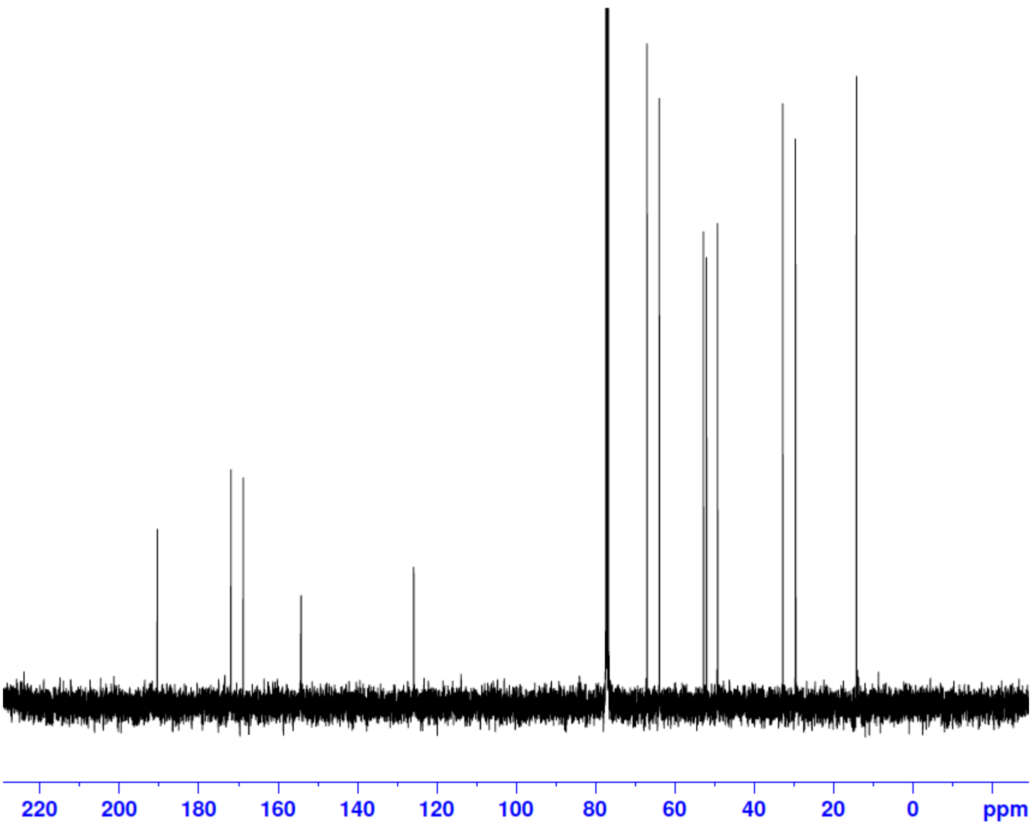

References

[1] A. B. Pangborn, M. A. Giardello, R. H. Grubbs, R. K. Rosen, F. J. Timmers, *Organometallics* **1996**, *15*, 1518-1520.

[2] B. Guzel, M. A. Omary, J. P. Fackler, A. Akgerman, *Inorg. Chim. Acta* **2001**, *325*, 45-50.

[3] S. Schweizer, J.-M. Becht, C. Le Drian, *Adv. Synth. Cat.* **2007**, *349*, 1150-1158.

[4] A. B. Chaplin, J. F. Hooper, A. S. Weller, M. C. Willis, *J. Am. Chem. Soc.* **2012**, *134*, 4885-4897.

[5] J. J. E. Donleavy, J., *J. Am. Chem. Soc.* **1940**, *62*, 220-221.

[6] F. Eisentrager, A. Gothlich, I. Gruber, H. Heiss, C. A. Kiener, C. Kruger, J. U. Notheis, F. Rominger, G. Scherhag, M. Schultz, B. F. Straub, M. A. O. Volland, P. Hofmann, *New J. Chem.* **2003**, *27*, 540-550.

[7] J. Cosier, A. M. Glazer, *J.App. Cryst.* **1986**, *19*, 105-107.

[8] Z. Otwinowski, W. Minor, *Macromolecular Cryst., Pt A* **1997**, *276*, 307-326.

[9] M. C. Burla, R. Caliandro, M. Camalli, B. Carrozzini, G. L. Cascarano, L. De Caro, C. Giacovazzo, G. Polidori, R. Spagna, *J. App.Cryst.* **2005**, *38*, 381-388.

[10] G. M. Sheldrick, *Acta Crystallographica Section A* **2008**, *64*, 112-122.

[11] M. Arambasic, J. F. Hooper, M. C. Willis, *Org. Lett.* **2013**, *15*, 5162-5165.
